# Supplementary material for: Sequential Regulation of Maternal mRNAs through a Conserved cis-Acting Element in Their 3′ UTRs
Source: Cell Rep. Author manuscript; Available in PMC 2019 Jan 10. (PMC6328254; doi:10.1016/j.celrep.2018.12.007)
Supplement: 1 [file NIHMS1517597-supplement-1.pdf]

Cell Reports, Volume *25*

Supplemental Information

Sequential Regulation of Maternal mRNAs through a Conserved *cis*-Acting Element in Their 3′ UTRs

Pooja Flora, Siu Wah Wong-Deyrup, Elliot Todd Martin, Ryan J. Palumbo, Mohamad Nasrallah, Andrew Oligney, Patrick Blatt, Dhruv Patel, Gabriele Fuchs, and Prashanth Rangan

Flora_Figure S1

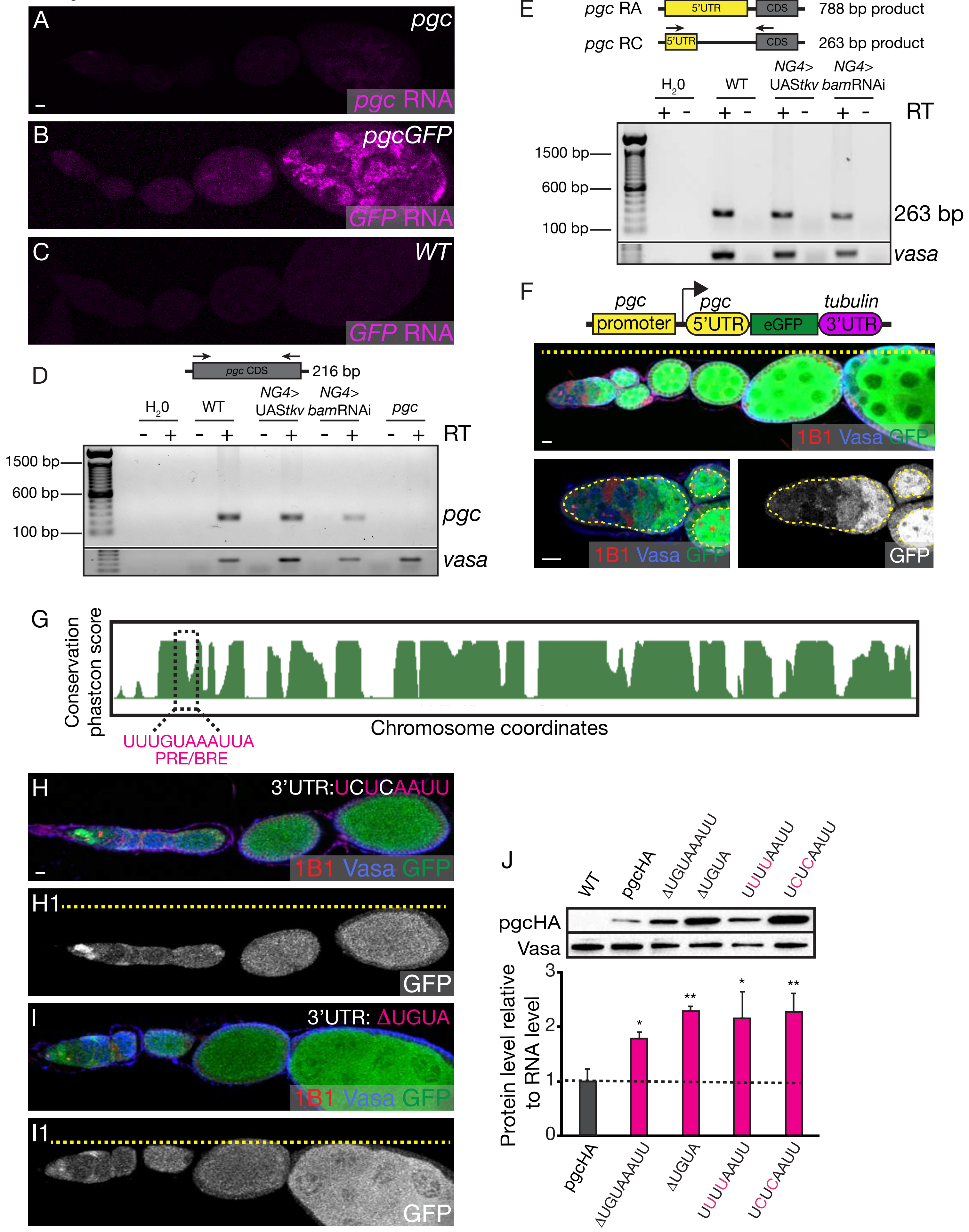

Supplemental Figure 1. Pgc is translationally regulated via its UTRs (Related to Figures 1 and 2) (A) The ovariole of a *pgc* mutant fly probed for *pgc* RNA (magenta) using FISH, show no signal for *pgc* RNA. (B) The ovariole of a *pgcGFP* transgenic fly probed for *GFP* RNA (magenta) using FISH, show similar expression pattern when compared to endogenous *pgc* RNA. (C) The ovariole of a wild-type fly probed for *GFP* RNA (magenta) using FISH, show no signal for *GFP* RNA. (D) RT-PCR of *pgc* CDS was carried out on RNA samples extracted from wild-type, *nosGAL4*>UAS*tkv* and *nosGAL4*>*bam*RNAi show *pgc RNA* is not only present in whole adult ovaries, but also transcribed in GSC and CB enriched tumors. RNA null *pgc* mutant was used as a negative control. RT-PCR of Vasa was carried out as a positive control. (E) RT-PCR of *pgc* 5'UTR was carried out on RNA samples extracted from wild-type, *nosGAL4*>UAS*tkv* and *nosGAL4*>*bam*RNAi. Primers were designed as to show either a 788bp or a 263bp product to confirm what 5'UTR length of *pgc RNA* was being expressed during oogenesis. Results showed presence of short version of *pgc 5'* UTR in whole adult ovaries, GSC and CB enriched tumors. RNA null *pgc* mutant was used as a negative control. RT-PCR of Vasa was carried out as a positive control. (F) The ovariole of a transgenic fly created by fusing GFP to the *pgc* 5' and *tub* 3'UTR and under the control of the *pgc* promoter was stained with 1B1 (red) which marks the spectrosomes and fusomes, Vasa (blue) which marks the germline and GFP (green) which marks Pgc expressing cells. There is a loss of GFP regulation throughout oogenesis, including at the earliest stages (yellow dashed line). (G) A phylogenetic analysis of *pgc* 3'UTR of all Drosophilids identified a conserved sequence that can potentially bind both RBPs, Pum and Bru. (H) The ovariole of a transgenic fly created by fusing GFP to *pgc* 5' and *pgc* 3'UTR where the UGUA sequence was mutated to UCUC (3'UTR: UCUCAAUU) and driven under *pgc* promoter stained with 1B1 (red), Vasa (blue) and GFP (green) shows loss of GFP regulation throughout oogenesis. GFP channel shown in H1. (I) Ovariole of a transgenic fly created by fusing GFP to *pgc* 5' and *pgc* 3'UTR where the UGUA sequence was deleted (3'UTR: ΔUGUA) and driven under *pgc* promoter stained with 1B1 (red), Vasa (blue) and GFP (green) shows loss of GFP regulation throughout oogenesis. GFP channel shown in I1. (J) Normalized protein expression to RNA levels shows that either deletions or mutations in the PRE/BRE sequence of the 3'UTR of *pgc* results in a significant upregulation of Pgc reporter protein when compared to FL 3'UTR. The graph represents an average generated from three independent biological replicates. The error bars are the standard error calculated from these replicates. A student t-test statistical analysis was performed. * indicates p-value <0.05 and ** indicates p-value <0.005. Scale bars: 10µm.

Flora_Figure S2

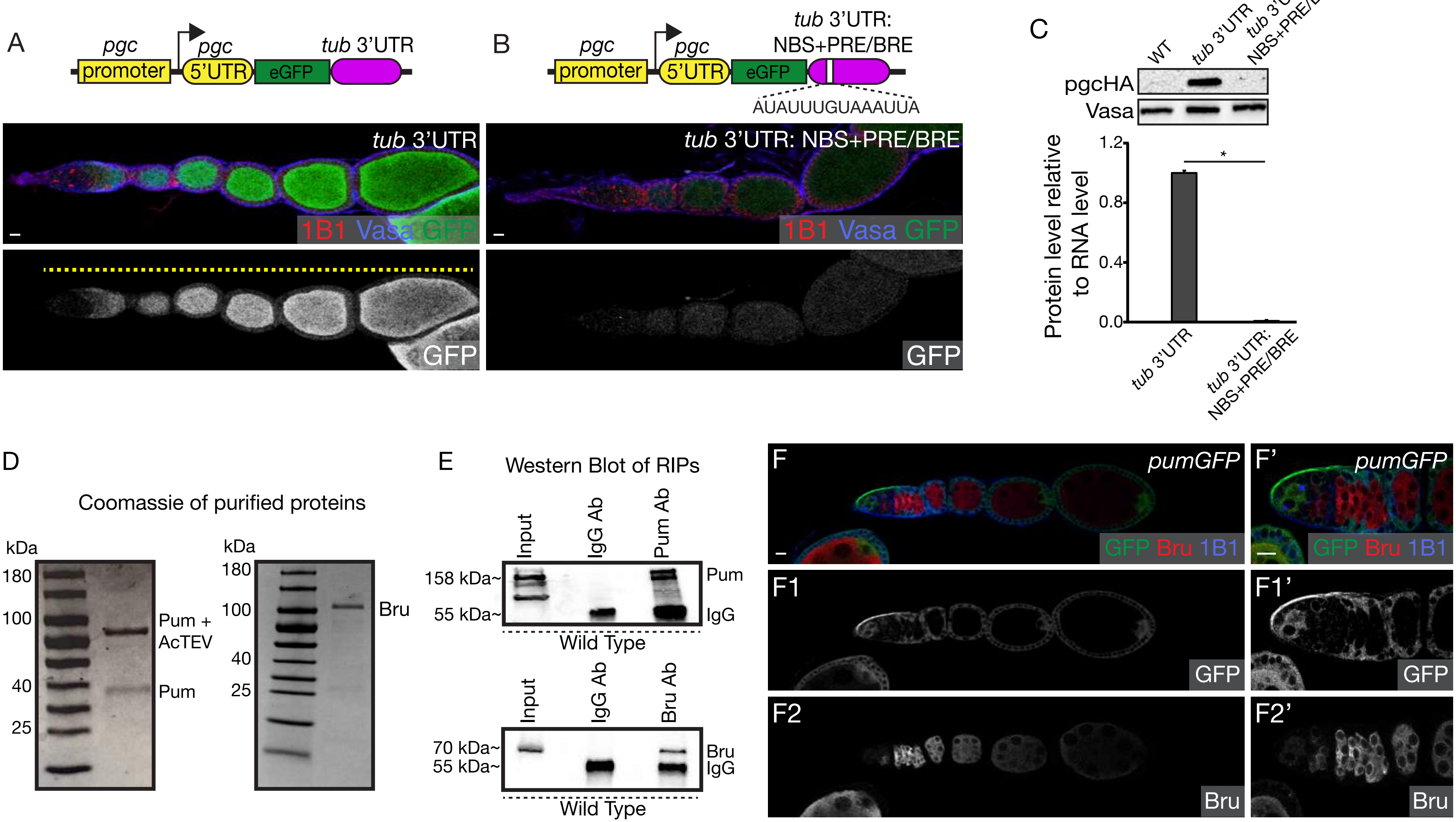

Supplemental Figure 2. A cis-element in the *pgc* 3'UTR that binds both Pum and Bru is required for translational control throughout oogenesis (Related to Figure 2) (A) The ovariole of a transgenic fly created by fusing GFP to the *pgc* 5' and *tub* 3'UTR and under the control of the *pgc* promoter stained with 1B1 (red) which marks the spectrosomes and fusomes, Vasa (blue) which marks the germline and GFP (green) shows a loss of GFP regulation throughout oogenesis (yellow dashed line). GFP channel is shown in A1. (B) The ovariole of a transgenic fly created by fusing GFP to the *pgc* 5' and *tub* 3'UTR that contains the NBS and PRE sequences and under the control of the *pgc* promoter stained with 1B1 (red), Vasa (blue) and GFP (green). Insertion of the sequence is sufficient translation repression. (C) Normalized protein expression to RNA levels shows that insertion of the NBS+PRE/BRE sequence in the *tub* 3'UTR results in significant repression of reporter protein when compared to control. The graph represents an average generated from three independent biological replicates. The error bars are the standard error calculated from these replicates. A student t-test statistical analysis was performed. * indicates p-value <0.05. (D) Commasie stained SDS-PAGE gels shows successful purification of recombinant Pum (left) and recombinant Bru protein (right). (E) Western Blot shows successful pull-down of Pum (top) and Bru (bottom) from wild-type ovary lysates using anti-Pum and anti-Bru antibody, respectively. (F-F2') *pumGFP* transgene fly stained with Bru (red), 1B1 (blue) and GFP (green) which marks Pum expressing cells shows that Pum protein is expressed in high levels in the earliest stages of oogenesis and lowers in later differentiating stages while Bru protein levels are low in early stages and increases from the 8-cell cyst stages and onwards. F1-F1' and F2-F2' shows GFP and Bru channels in gray. Scale bars: 10μm.

Flora_Figure S3

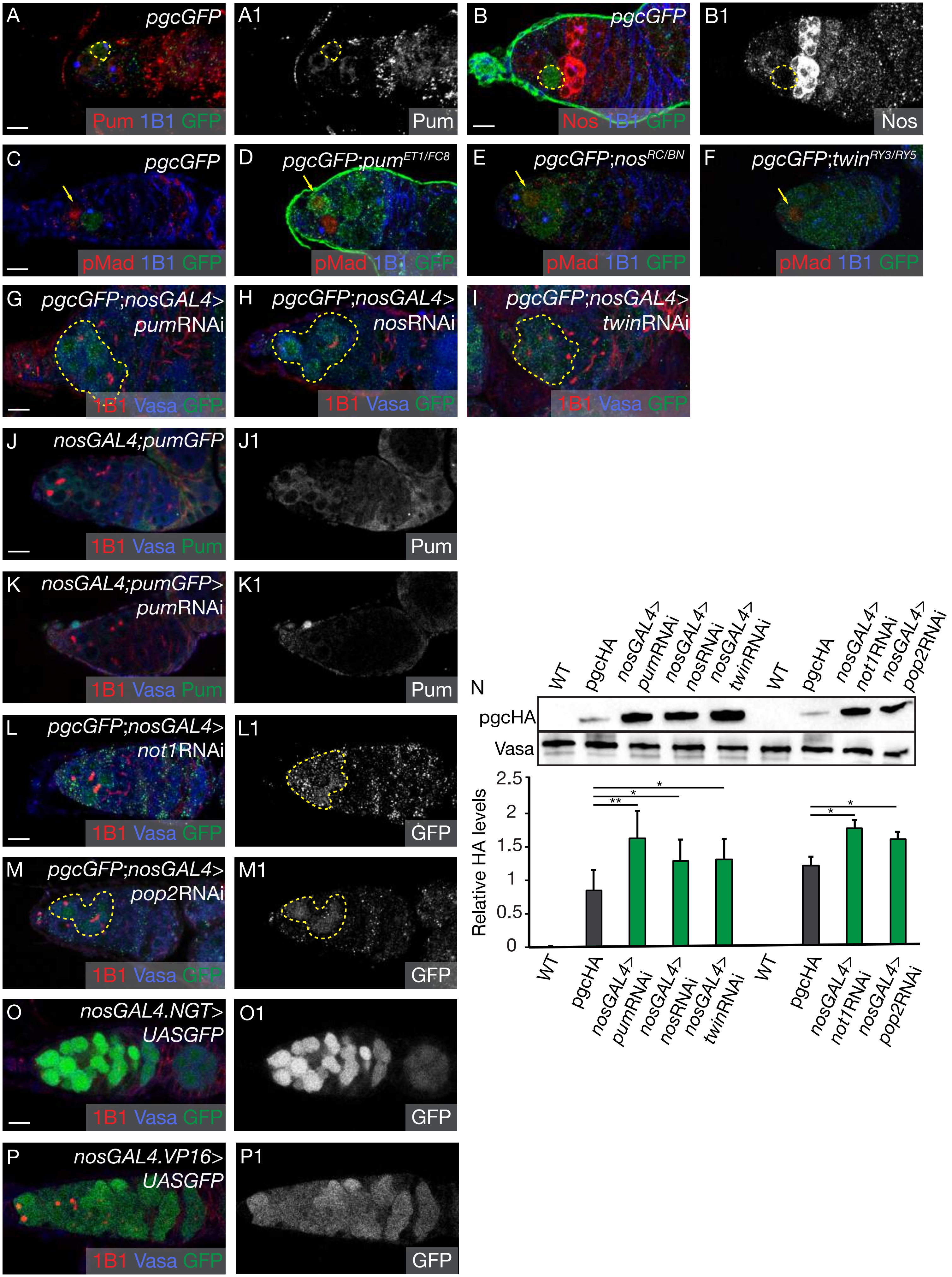

Supplemental Figure 3. Pum and its co-factor Nos regulate Pgc translation in the GSCs (Related to Figure 3) (A, A1) The germarium of *pgcGFP* fly stained with Pum (red), 1B1 (blue) which marks fusomes and spectrsomes and GFP (green) which marks Pgc expressing cells shows high levels of Pum protein in the GSC, pre-CB (yellow dashed circle) 2- to 4-cell cysts. Pum staining is shown in gray in A1. (B, B1) The germarium of *pgcGFP* fly stained with Nos (red), 1B1 (blue) and GFP (green) which marks Pgc expressing feels shows Nos protein is present throughout the germarium except for the GFP expressing pre-CB cell (yellow dashed circle). Nos staining is shown in gray in B1. (C) The germarium of *pgcGFP* fly stained with pMad (red) which marks GSCs, 1B1 (blue) and GFP (green) shows GSCs do not express GFP (yellow arrow). (D-F) The germaria of *pgcGFP, pgcGFP; pum, pgcGFP; nos* and *pgcGFP; twin* stained with pMad (red), 1B1 (blue) and GFP (green) show that in absence of Pum and its co-factors, there is a loss of GFP regulation in the GSCs (yellow arrow). (G-I) The germaria of *pgcGFP; nosGAL4>pum*RNAi, *pgcGFP; nosGAL4>nos*RNAi and *pgcGFP; nosGAL4>twin*RNAi flies stained with 1B1 (red), Vasa (blue) and GFP (green) show aberrant expression of GFP in the earliest stages of oogenesis, including the GSCs (outlines in yellow dashed line). (J, J1) The germarium of *nosGAL4* flies stained with 1B1 (red), Vasa (blue) and Pum (green) shows Pum being expressed in high levels in somatic cells and in the earliest stages of oogenesis. Pum channel shown in J1. (K, K1) The germarium of *nosGAL4; pum*RNAi flies stained with 1B1 (red), Vasa (blue) and Pum (green) shows Pum is significantly downregulated in the ovaries that contain germline. Pum channel shown in K1 (L, L1) The germarium of germline depleted *not1* ovary stained with 1B1 (red), Vasa (blue) and GFP (green) shows aberrant expression of GFP in the GSCs and 4-cell cysts (100%, n= 25 germaria) (outlined in yellow dashed line). GFP channel showed in gray scale in A1. (M, M1) The germarium of germline depleted *pop2* ovary stained with 1B1 (red), Vasa (blue) and GFP (green) shows aberrant expression of GFP in the GSCs to the 4-cell cyst stages (100%, n= 25 germaria) (outlined in yellow dashed line). GFP channel showed in gray scale in B1. (N) A western blot analysis shows a significant upregulation of Pgc reporter protein in the germline depletion of *pum*, *nos*, *twin*, *not1,* and *pop2* ovaries when compared to *pgcGFP.* The graph represents an average generated from three independent biological replicates. The error bars are the standard error calculated from these replicates. A student t-test statistical analysis was performed. * indicates p-value <0.05 and ** indicates p-value <0.005. (O, O1) The germarium of *nosGAL4.NGT>UAS-GFP* stained with 1B1 (red), Vasa (blue) and GFP (green) shows no difference in GFP expression levels in the germarium. GFP channels shown in O1. (P, P1) The ovariole of *nosGAL4.VP16>UAS-GFP* stained with 1B1 (red), Vasa (blue) and GFP (green) shows no difference in GFP expression levels in the germarium. GFP channels shown in P1. Scale bars: 10μm.

Flora_Figure S4

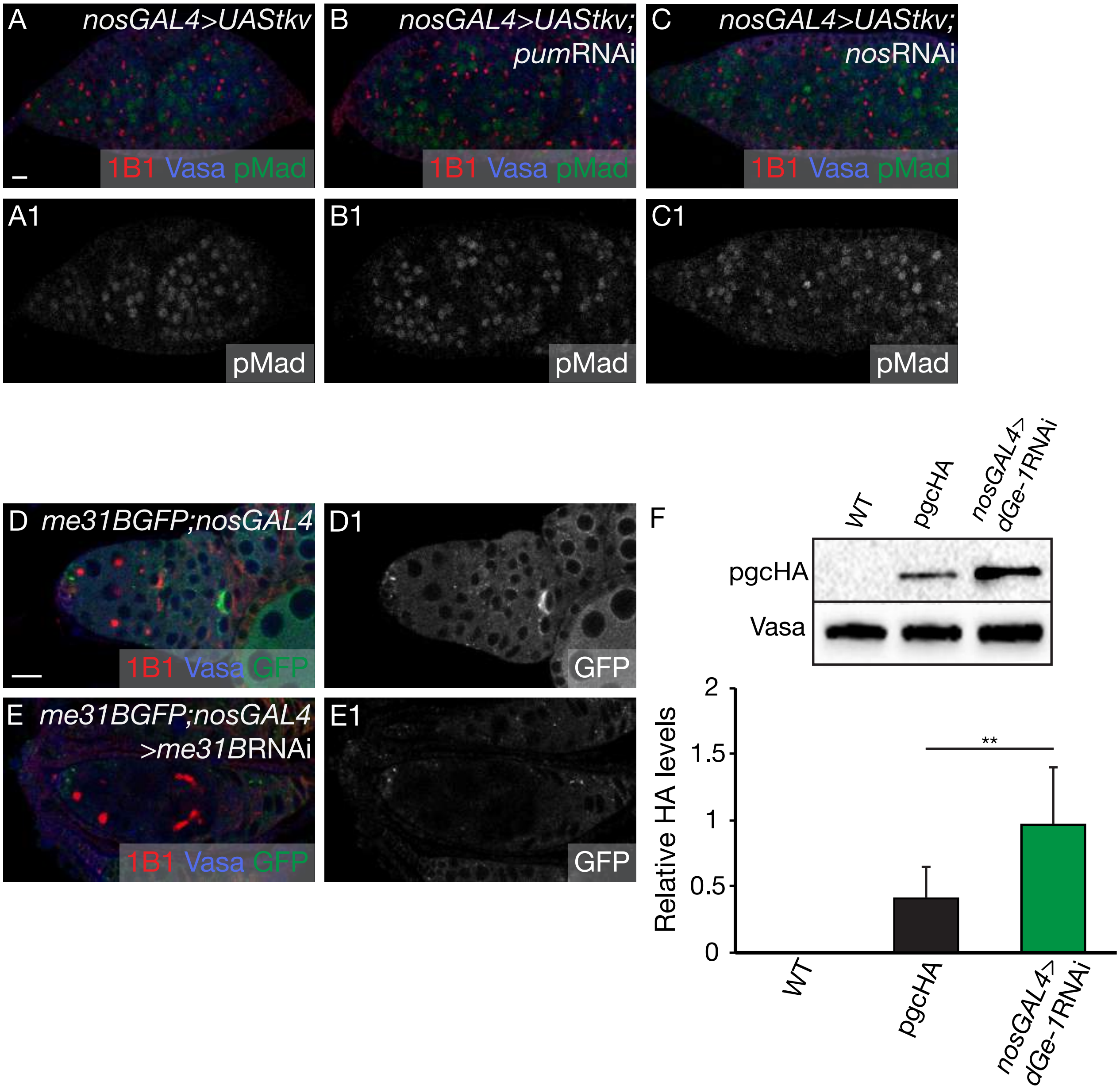

Supplemental Figure 4. Me31B cooperates with the decapping protein dGe-1 and *pgc* 5'UTR to mediate repression in the GSCs and early differentiating cysts (Related to Figures 3 and 4) (A, A1) The germarium of *nosGAL4*>UAS-*tkv* ovary stained with 1B1 (red) which marks the spectrosomes and fusomes, Vasa (blue) which marks the germline and pMad (green) which marks GSCs shows a tumor enriched with GSCs. pMad channel shown in A1. (B-C1) The germaria of *nosGAL4*>UAS-*tkv; pum*RNAi, *nosGAL4*>UAS-*tkv; nos*RNAi ovary stained with 1B1 (red), Vasa (blue) and pMad (green) shows a tumor of enriched with GSCs. pMad channel shown in B1 and C1. (D, D1) The germarium of *me31BGFP*-trap; *nosGAL4* ovary stained with 1B1 (red), Vasa (blue) and GFP (green) which marks Me31B expressing cells shows Me31B being expressed in both the germline and somatic cells of the germarium. GFP channel shown in D1. (E, E1) The germarium of *me31BGFP*-trap; *nosGAL4* depleted of *me31B* via RNAi stained with 1B1 (red), Vasa (blue GFP (green) Me31B being expressed only in the somatic cells of the germarium confirming germline knockdown of Me31B via RNAi. GFP channel shown in E1. (F) A western blot analysis shows a significant upregulation of Pgc reporter protein in the germline depletion of *dGe-1* ovaries when compared to *pgcGFP.* The graph represents an average generated from three independent biological replicates. The error bars are the standard error calculated from these replicates. A student t-test statistical analysis was performed. * indicates p-value <0.05 and ** indicates p-value <0.005. We were unsuccessful in isolating stable lysates from Me31B depleted ovaries to carry out a WB analysis. Scale bars: 10μm.

Flora_Figure S5

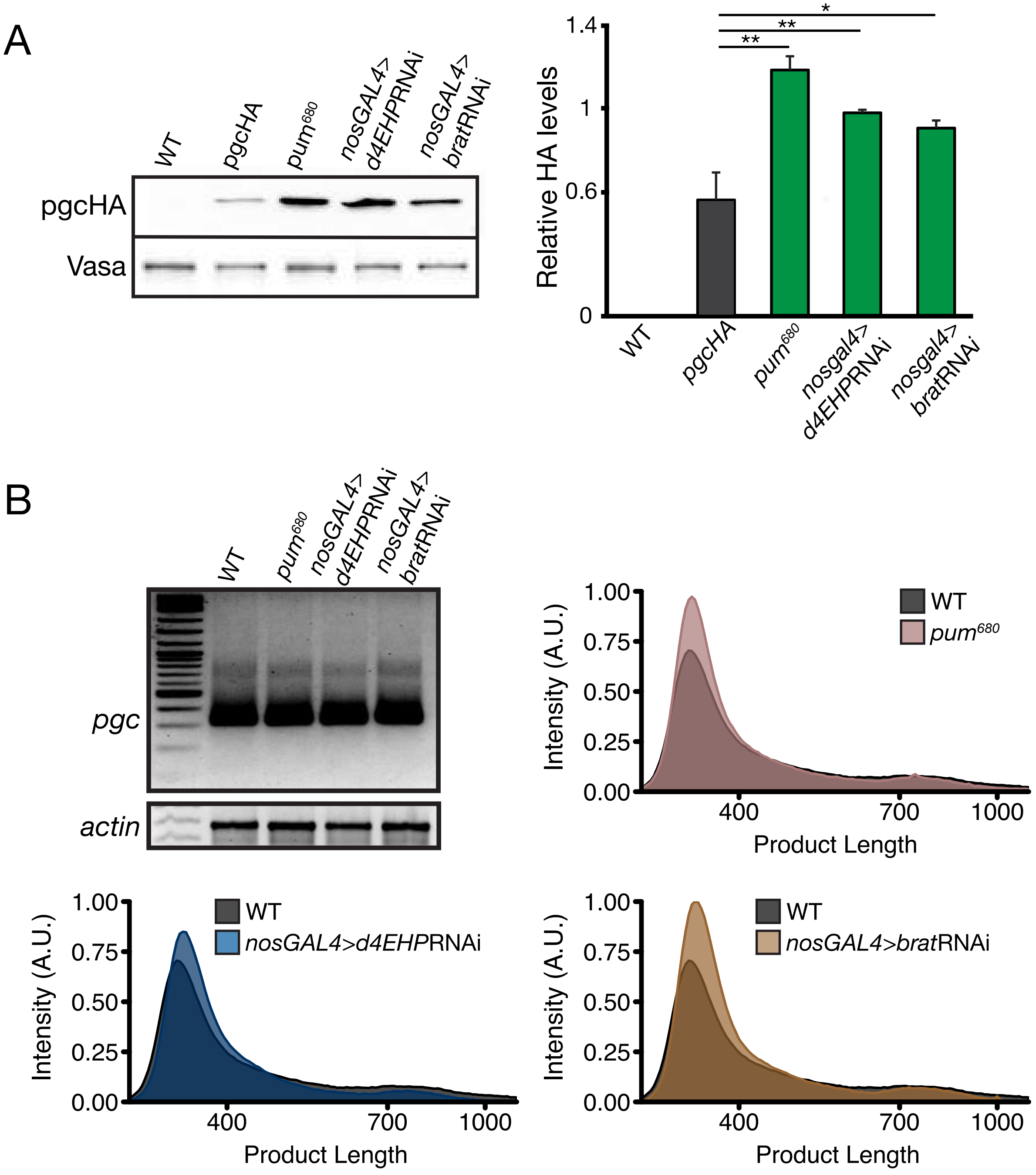

Supplemental Figure 5. Pum and its co-factor Brat regulate Pgc translation in the 4- to 16-cell cysts (Related to Figure 5) (A) A western blot analysis shows a significant upregulation of Pgc reporter protein in *pum*^{680} and the germline depletion of *brat* and *d4EHP* ovaries when compared to *pgcGFP.* The graph represents an average generated from three independent biological replicates. The error bars are the standard error calculated from these replicates. A student t-test statistical analysis was performed. ** indicates p-value <0.005. (B) PAT assay analysis of *pgc* poly(A)-tail length in wild-type, *pum*^{680} and germline depletions of d4EHP and Brat show that loss of these factors do not result in any change of poly(A)-tail length of *pgc*.

Flora_Figure S6

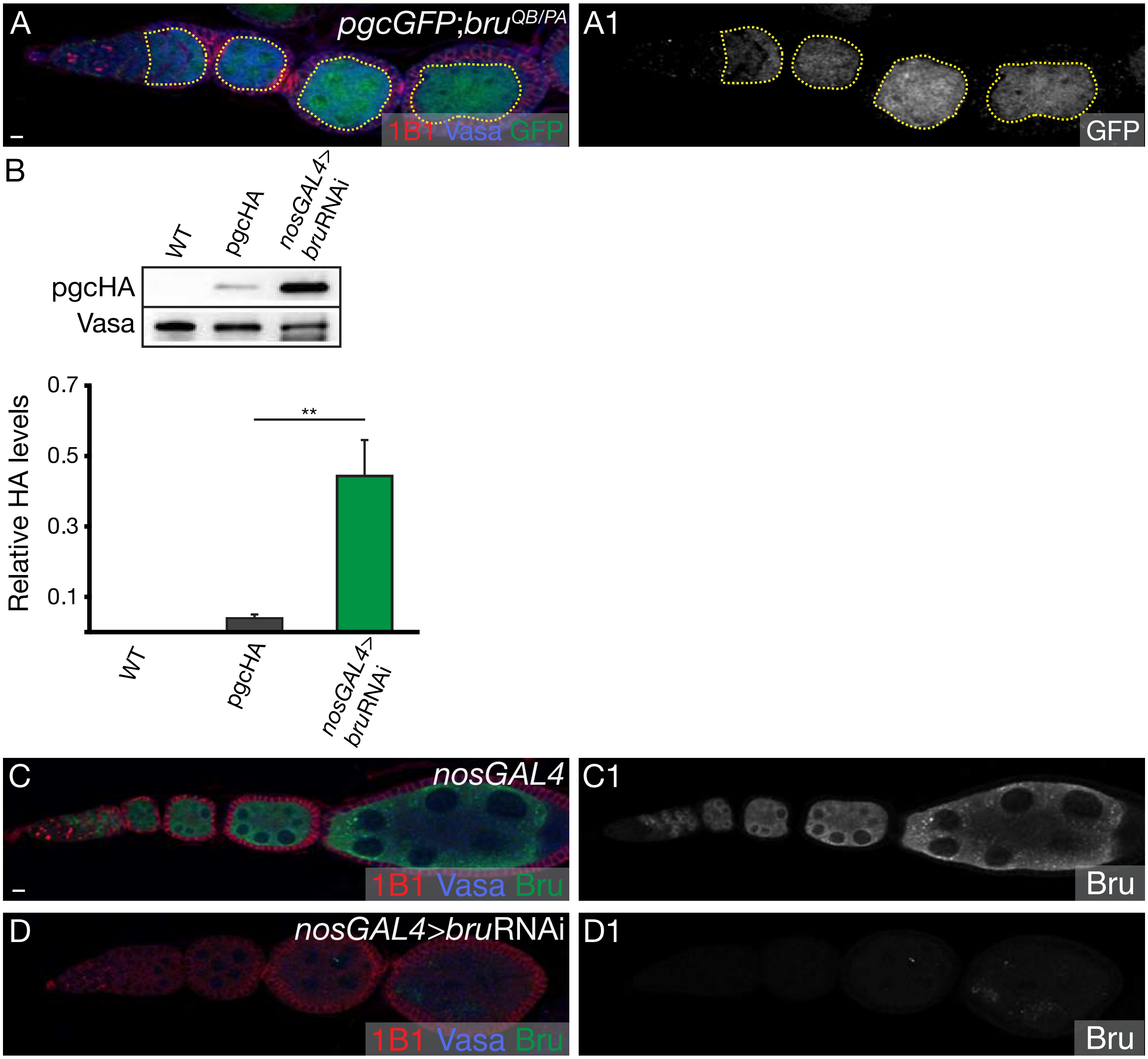

Supplemental Figure 6. Bru and Cup regulate Pgc translation in the later stages of oogenesis (Related to Figure 6) (A, A1) The ovariole of *pgcGFP; bru*RNAi$^{QB/PA}$ stained with 1B1 (red) which marks the spectrosomes and fusomes, Vasa (blue) which marks the germline and GFP (green) which marks Pgc expressing cells shows upregulation of reporter expression from 16-cell cyst onwards (outlined in yellow dashed line). GFP channel shown in A1. (B) A western blot analysis shows a significant upregulation of Pgc reporter protein in the germline depletion of Bru ovaries when compared to *pgcGFP.* We were unsuccessful in isolating stable lysates from Cup depleted ovaries to carry out a WB analysis. The graph represents an average generated from three independent biological replicates. The error bars are the standard error calculated from these replicates. A student t-test statistical analysis was performed. ** indicates p-value <0.005. (C, C1) The ovariole of control *nosGAL4* ovary stained with 1B1 (red), Vasa (blue) and Bru (green) shows Bru being expressed from 16-cell cyst and onwards. Bru channel shown in C1. (D, D1) The ovariole of *nosGAL4>bru*RNAi stained with 1B1 (red), Vasa (blue) and Bru (green) shows little or no Bru expression in the ovariole. GFP channel shown in D1. Scale bars: 10μm.

Flora_Figure S7

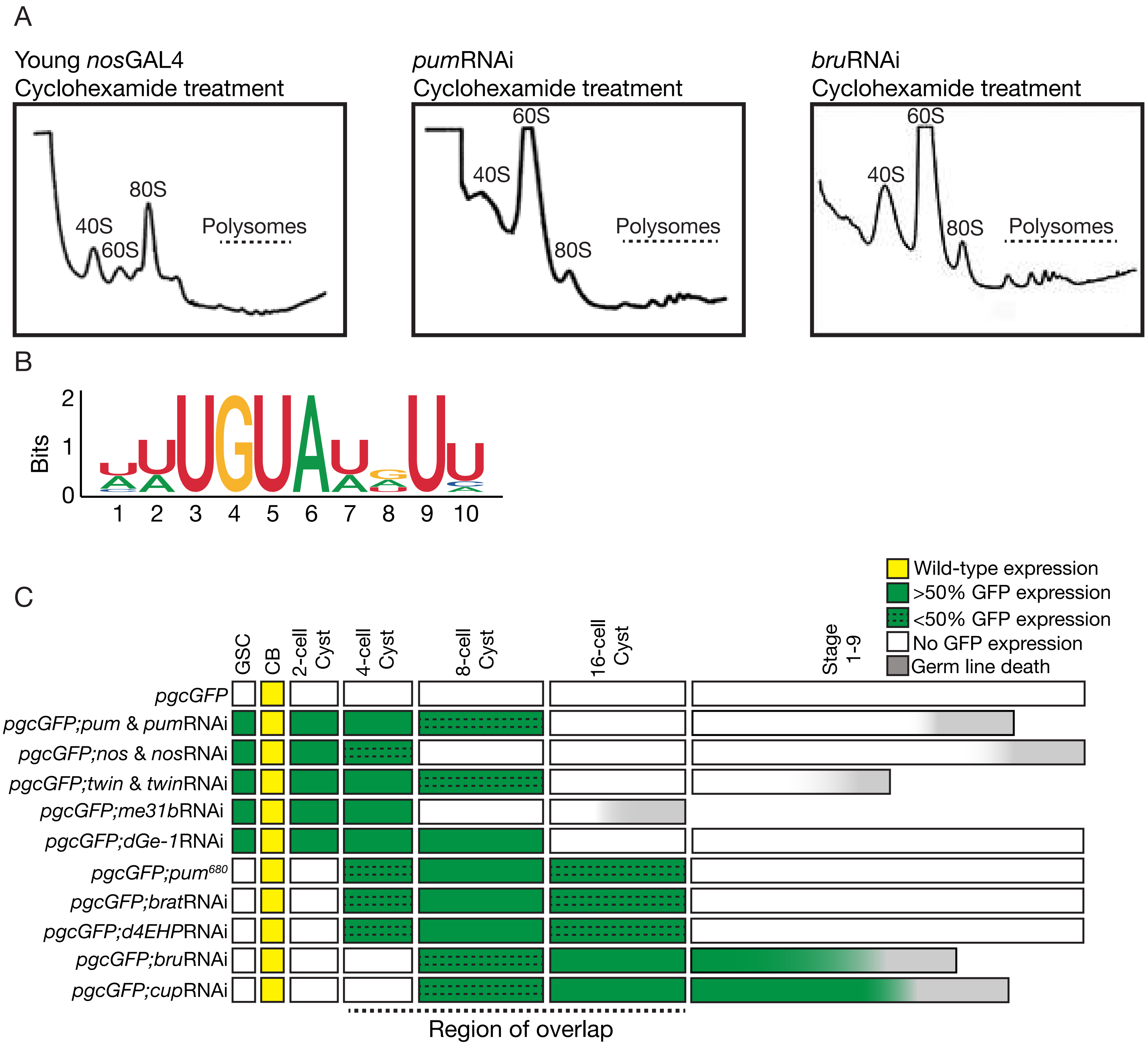

Supplemental Figure 7. A class of germline RNAs are similarly regulated by both Pum and Bru (Related to Figure 7) (A) Polysome profile traces of young wild-type, *pgcGFP; nosGAL4>pum*RNAi, and *pgcGFP; nosGAL4>bru*RNAi ovaries treated with cyclohexamide. (B) The logo of the sequences used to identify shared targets of Pum and Bru mediated regulation that contain a sequence similar to the PRE/BRE sequence identified in the *pgc* 3'UTR. (C) A developmental profile of GFP expression in *pgcGFP, pgcGFP; pum*$^{ET1/FC8}$ and germline knockdown of *pum, pgcGFP; nos*$^{RC/BN}$ and germline knockdown of *nos* and *pgcGFP; twin*$^{ry3/ry5}$ and germline knockdown of *twin, pgcGFP; me31B*RNAi, *pgcGFP; dGe-1*RNAi, *pgcGFP; pum*^{680}, *pgcGFP; nosGAL4>brat*RNAi, *pgcGFP; nosGAL4>d4EHP*RNAi, *pgcGFP; nosGAL4>bru*RNAi, and *pgcGFP; nosGAL4>cup*RNAi ovarioles show temporal and sequential loss of GFP regulation in different stages of oogenesis where these trans-acting factors mediate *pgc* regulation.
