# Supplementary material for: Sequential Regulation of Maternal mRNAs through a Conserved cis-Acting Element in Their 3′ UTRs
Source: Cell Rep. Author manuscript; Available in PMC 2019 Jan 10. (PMC6328254; doi:10.1016/j.celrep.2018.12.007)
Supplement: 3 [file NIHMS1517597-supplement-3.pdf]

Cell Reports

Article

Sequential Regulation of Maternal mRNAs through a Conserved *cis*-Acting Element in Their 3′ UTRs

Graphical Abstract

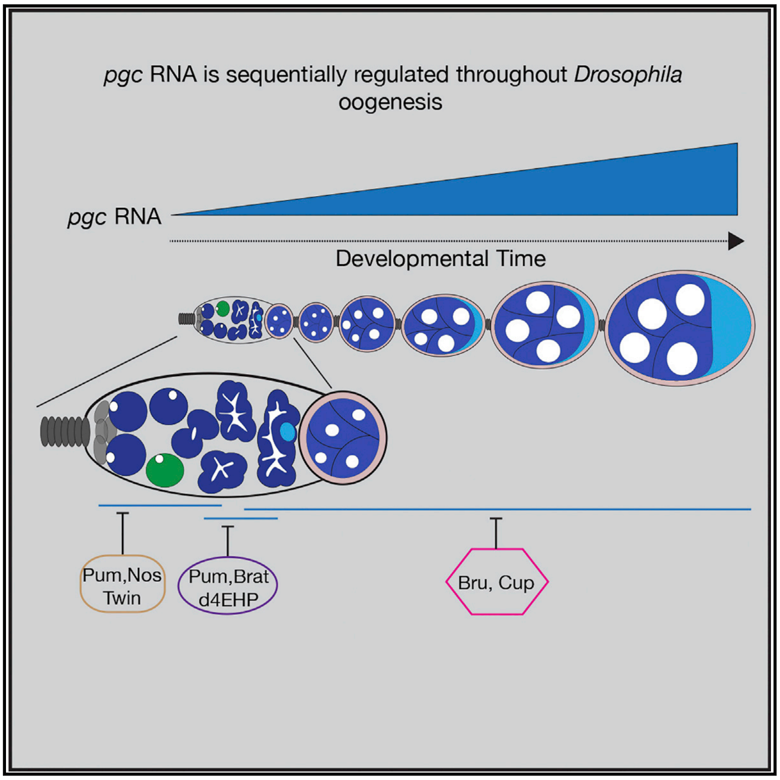

Authors

Pooja Flora, Siu Wah Wong-Deyrup, Elliot Todd Martin, ..., Dhruv Patel, Gabriele Fuchs, Prashanth Rangan

Correspondence

prangan@albany.edu

In Brief

Flora et al. show that *pgc*, a germline determinant, is translationally regulated throughout oogenesis. Different conserved RBPs bind a 10-nt sequence in the 3′ UTR to continuously repress translation throughout oogenesis. This mode of regulation applies to a class of maternal mRNAs, including *zelda*, the activator of the zygotic genome.

Highlights

- *pgc*, a germline RNA, is translationally regulated throughout *Drosophila* oogenesis
- A conserved 10-nt sequence in the *pgc* 3′ UTR is required for its regulation
- Pum and Bru, conserved RBPs, sequentially repress *pgc* translation via this sequence
- A class of maternal RNAs are also regulated by Pum and Bru during oogenesis

Flora et al., 2018, Cell Reports *25*, 3828–3843
December 26, 2018 © 2018 The Author(s).
https://doi.org/10.1016/j.celrep.2018.12.007

CellPress

OPEN ACCESS
CellPress

Cell Reports
Article

Sequential Regulation of Maternal mRNAs through a Conserved *cis*-Acting Element in Their 3′ UTRs

Pooja Flora,[1,2] Siu Wah Wong-Deyrup,[1] Elliot Todd Martin,[1] Ryan J. Palumbo,[1,3] Mohamad Nasrallah,[1,4] Andrew Oligney,[1,5] Patrick Blatt,[1] Dhruv Patel,[1,6] Gabriele Fuchs,[1] and Prashanth Rangan[1,7,*]
[1]Department of Biological Sciences/RNA Institute, University at Albany SUNY, Albany, NY 12222, USA
[2]Present address: Department of Cell, Developmental and Regenerative Biology, Icahn School of Medicine at Mount Sinai, New York, NY 10129, USA
[3]Present address: Department of Biochemistry and Molecular Biology, Upstate Medical University, SUNY, Syracuse, NY 13210, USA
[4]Present address: University of Massachusetts Medical School, Worcester, MA 01605, USA
[5]Present address: Touro College of Osteopathic Medicine (TouroCOM), Middletown, NY 10940, USA
[6]Present address: Albany Medical College, Albany, NY 12208, USA
[7]Lead Contact
*Correspondence: prangan@albany.edu
https://doi.org/10.1016/j.celrep.2018.12.007

SUMMARY

Maternal mRNAs synthesized during oogenesis initiate the development of future generations. Some maternal mRNAs are either somatic or germline determinants and must be translationally repressed until embryogenesis. However, the translational repressors themselves are temporally regulated. We used *polar granule component* (*pgc*), a *Drosophila* maternal mRNA, to ask how maternal transcripts are repressed while the regulatory landscape is shifting. *pgc*, a germline determinant, is translationally regulated throughout oogenesis. We find that different conserved RNA-binding proteins bind a 10-nt sequence in the 3′ UTR of *pgc* mRNA to continuously repress translation at different stages of oogenesis. Pumilio binds to this sequence in undifferentiated and early-differentiating oocytes to block Pgc translation. After differentiation, Bruno levels increase, allowing Bruno to bind the same sequence and take over translational repression of *pgc* mRNA. We have identified a class of maternal mRNAs that are regulated similarly, including *zelda*, the activator of the zygotic genome.

INTRODUCTION

The germline gives rise to eggs and sperm that launch the next generation. Upon fertilization, the egg differentiates into every cell lineage of the adult organism, including a new germline, and is therefore totipotent (Seydoux and Braun, 2006; Cinalli et al., 2008). Pivotal to the task of kick-starting the next generation is a maternally synthesized trust fund of mRNAs deposited into the egg during oogenesis (Lasko 2012). After fertilization, and prior to zygotic genome activation, translation of these maternally supplied mRNAs helps power early development (Zhang and Smith, 2015; Tadros and Lipshitz, 2009; Lee et al., 2014). Some of the maternally supplied mRNAs code for key determinants of both somatic and germline cell fate and thus need to be exquisitely regulated during oogenesis and early embryogenesis.

RNA-binding proteins (RBPs) regulate the maternal mRNAs through interactions with sequences within the 3′ UTRs of their target mRNAs (Rosario et al., 2017; Slaidina and Lehmann, 2014; Johnstone and Lasko, 2001). Loss of RBPs during oogenesis results in death, sterility, or germline to soma *trans*-differentiation (Ciosk et al., 2006; Forbes and Lehmann, 1998). This suggests that RBPs are critical for silencing key somatic and germline determinants during oogenesis. Consistent with this observation, it has been shown that gene regulation during oogenesis and early embryogenesis relies primarily on the 3′ UTRs of mRNAs rather than on their promoters (Merritt et al., 2008; Rangan et al., 2009). Additionally, loss of specific sequences in the 3′ UTR of maternal mRNAs results in their dysregulation (Kim-Ha et al., 1995; Wharton and Struhl, 1991). However, several RBPs that are regulators of translation also fluctuate in levels of expression, with these fluctuations promoting critical developmental transitions. For example, during *C. elegans* oogenesis, GLD-1 and MEX-3, two RBPs whose loss results in germline to soma *trans*-differentiation, have a reciprocal expression pattern (Mootz et al., 2004; Ciosk et al., 2006; Draper et al., 1996). In human fetal ovary, RBPs such as deleted in azoospermia-like (DAZL) play an important role in regulating RNA targets, such as *TEX11*, a gene required for recombination and DNA repair, via its 3′ UTR (Rosario et al., 2017). During human oogenesis, DAZL has a dynamic expression pattern; it is robustly expressed in the pre-meiotic and post-meiotic germ cells but absent during meiotic stages (Anderson et al., 2007; He et al., 2013). The conundrum remains as to how mRNAs can be continually silenced during oogenesis when the RBPs that regulate them fluctuate.

Drosophila oogenesis is an excellent model to investigate how maternal mRNAs are continuously regulated. Oogenesis in *Drosophila* begins when germline stem cells (GSCs) divide to both self-renew and give rise to a stem cell daughter called a cystoblast (CB) (Figures 1A and 1B) (Chen and McKearin, 2003). The CB differentiates by undergoing four incomplete mitotic divisions to give rise to 2-, 4-, 8-, and 16-cell cysts

 © 2018 The Author(s).
This is an open access article under the CC BY license (http://creativecommons.org/licenses/by/4.0/).

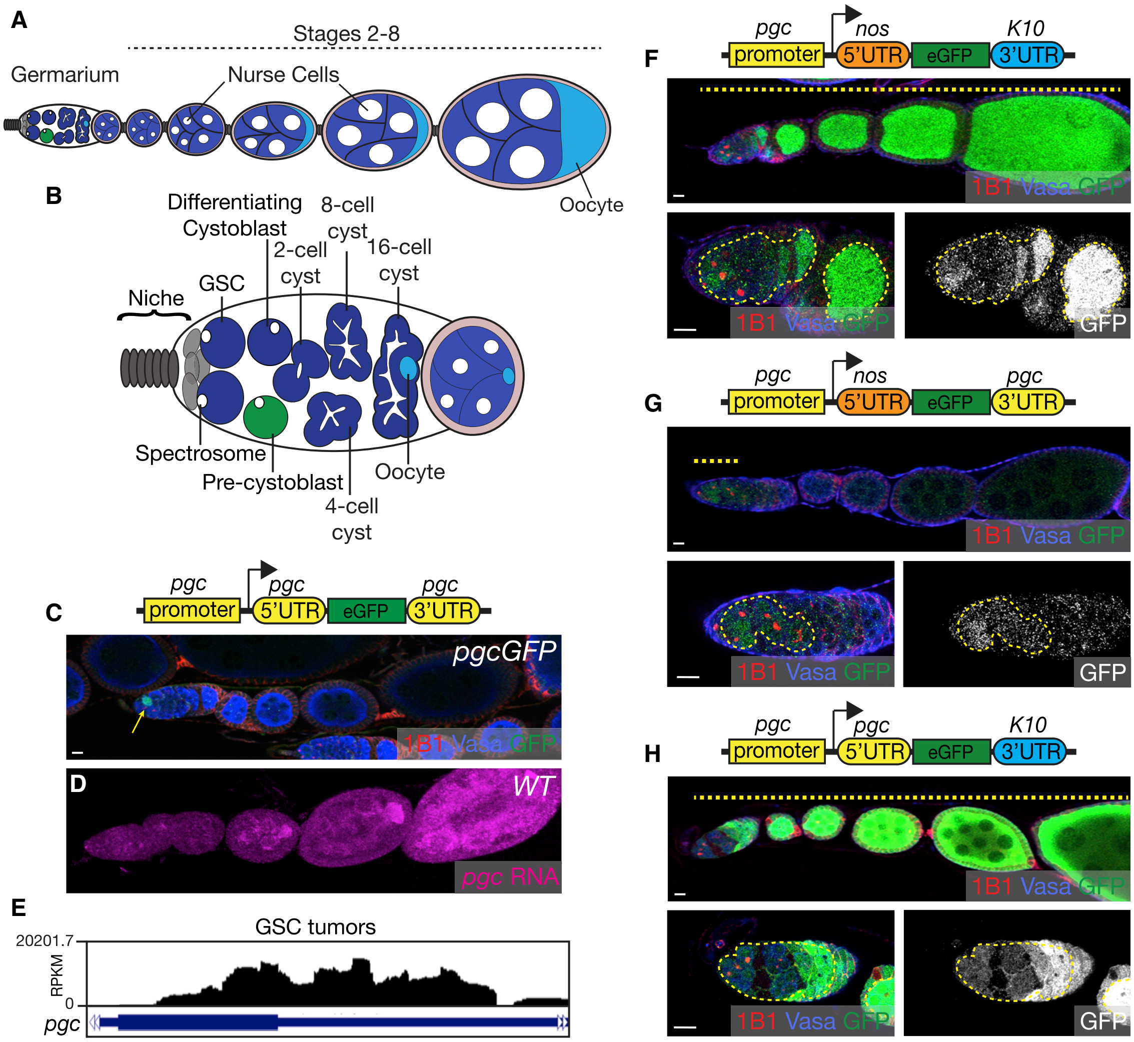

Figure 1. Pgc Is Translationally Regulated via Its UTRs

(A) Schematic representation of a *Drosophila* ovariole.

(B) Schematic representation of a germarium housing the germline stem cells (GSCs) (blue), pre-cystoblast (pre-CB) (green), and differentiating cysts. The single cells of the germarium can be identified by spectrosomes and the differentiating cysts can be identified by fusomes.

(C) The ovariole of a *pgcGFP* ovary stained with 1B1 (red), which marks the spectrosomes and fusomes; Vasa (blue), which marks the germline; and GFP (green), which marks Pgc-expressing cells. Expression of GFP is restricted to the pre-CB (arrow).

(D) The ovariole of a wild-type fly probed for *pgc* RNA (magenta) using FISH shows that *pgc* RNA is present throughout oogenesis.

(E) RNA-seq track of *pgc* in *nosGAL4 > UAS-tkv* ovaries.

(F) The ovariole of a transgenic fly (*pgc* promoter-*nos* 5′ UTR-GFP-*K10* 3′ UTR) stained with 1B1 (red), Vasa (blue), and GFP (green). GFP expression shows that *pgc* promoter is active throughout oogenesis (dashed line).

(G) The ovariole of a transgenic fly (*pgc* promoter-*nos* 5′ UTR-GFP- *pgc* 3′ UTR) stained with 1B1 (red), Vasa (blue) and GFP (green) shows GFP expression only in the earliest stages of oogenesis (dashed line).

(H) The ovariole of a transgenic fly (*pgc* promoter-*pgc* 5′ UTR-GFP-*K10* 3′ UTR) stained with 1B1 (red), Vasa (blue), and GFP (green) shows GFP expression throughout oogenesis (dashed line).

Scale bars, 10 μm. See also Figure S1.

(Figure 1B) (McKearin and Ohlstein, 1995; McKearin and Spradling, 1990). Of these 16 cells, one is designated as the oocyte and the others become nurse cells (Figure 1A) (Spradling et al., 1997); the maternal mRNAs and proteins synthesized by the nurse cells are deposited into the oocyte (Spradling 1993). The oocyte and surrounding nurse cells are encapsulated by somatic cells to form an egg chamber, which progresses through successive developmental stages (Margolis and Spradling, 1995; Gilboa and Lehmann, 2004). These maternal mRNAs that are deposited into the oocyte need to be post-transcriptionally regulated to promote proper oogenesis and embryogenesis (Richter and Lasko, 2011; Lasko 2012; Laver et al., 2015).

Polar granule component (*pgc*) is a superb candidate to address how maternal mRNAs are regulated during oogenesis developmental transitions. During oogenesis, *pgc* is synthesized and provided to the oocyte, where it localizes to the germ plasm (Nakamura et al., 1996). While *pgc* mRNA is continuously present, Pgc is only translated in two short pulses: in the CB during oogenesis and in the germ cells during embryogenesis (Hanyu-Nakamura et al., 2008; Flora et al., 2018). Pgc expression in the CB is required to promote timely differentiation (Flora et al., 2018), while expression of Pgc in the germ cells is required to repress the expression of somatic genes that could interfere with germline specification (Hanyu-Nakamura et al., 2008). Pgc performs these tasks by causing global transcriptional silencing through targeting the basal transcriptional elongation machinery of RNA polymerase II (Martinho et al., 2004; Hanyu-Nakamura et al., 2008; Flora et al., 2018). *pgc* can even suppress transcription in other cell types upon ectopic expression (Timinszky et al., 2008). The strong effects of Pgc on transcription lead to a requirement for strict regulation of *pgc* translation in cells where it is normally found. It is known that the 3′ UTR of *pgc* mRNA is sufficient to mediate translational control after GSC differentiation into an oocyte (Rangan et al., 2008); however, it is not known whether *pgc* is regulated transcriptionally or translationally prior to differentiation nor what *trans*-acting factors regulate *pgc* translation after differentiation.

Temporally restricted RBPs that bind to 3′ UTRs regulate developmental transitions during *Drosophila* oogenesis by controlling translation of their targets. Pumilio (Pum), an RBP that belongs to the conserved Pum- and Fem-3-binding factor (PUF) family of proteins, is present at high levels in the undifferentiated cells in the ovary, including GSCs, CBs, and early-differentiating cysts (Lin and Spradling 1993; Forbes and Lehmann, 1998). Pum represses the translation of differentiation-promoting mRNAs in GSCs, thereby preventing stem cell loss (Forbes and Lehmann, 1998; Joly et al., 2013). Pum expression is attenuated in the differentiated stages, allowing for the expression of the differentiation-promoting mRNAs (Forbes and Lehmann, 1998; Carreira-Rosario et al., 2016). *Drosophila* Bruno 1 (Bru), a CUGBP and ETR-3-like factor (CELF) superfamily protein, is expressed at increasing levels during differentiation and is then maintained for the rest of oogenesis (Xin et al., 2013; Sugimura and Lilly, 2006; Webster et al., 1997). Bru regulates several maternal mRNAs post-differentiation during oogenesis (Schüpbach and Wieschaus, 1991; Webster et al., 1997; Snee et al., 2014). Thus, Pum and Bru have reciprocal temporal regimes and could act jointly to repress targets throughout oogenesis. However, it is not known whether further repression is required of Pum targets after differentiation or Bru targets prior to differentiation.

Pum and Bru can use various cofactors to mediate translational repression using distinct mechanisms. Pum partners with Nanos (Nos) to recruit translation modulators such as Twin, a deadenylase causing a shortening of the poly(A)-tail (Joly et al., 2013). Pum can also recruit brain tumor (Brat), which is known to modulate translation by interacting with *Drosophila* eukaryotic translation initiation factor 4E homologous protein (d4EHP), a cap-binding protein (Cho et al., 2006; Harris et al., 2011). Bru can form oligomers to form silencing particles or can partner with Cup, which associates with the 5′ cap-binding initiation factor eIF4E, to regulate mRNAs (Nakamura et al., 2004; Kim-Ha et al., 1995; Chekulaeva et al., 2006). Why certain mechanisms are preferred over others is not known.

Here, we elucidate a control mechanism that ensures handoff of translational repression of a germline determinant, *pgc*, from one set of regulators to another. This governs the critical expression of Pgc just in CBs, ensuring proper maintenance of GSCs and their conversion into differentiated progeny. We demonstrate that this control depends on a 10-nt sequence in the 3′ UTR of *pgc* mRNA. In the undifferentiated stages, we find that Pum binds the 10-nt sequence and partners with Nos and the CCR4-Not complex to regulate *pgc* mRNA in a poly(A)-dependent manner. When Nos levels drop in CBs, *pgc* is expressed. After CB differentiation, Pum switches partners to use Brat to suppress *pgc* in the early-differentiating cysts in a cap-dependent manner. However, when Pum levels diminish, *pgc* mRNA is bound by Bru via the same 10-nt sequence to translationally regulate it. Bru recruits Cup to silence *pgc* translation also in a cap-dependent manner. We find that a class of maternal mRNAs, including *zelda*, which play pivotal roles during development, are also regulated by both Pum and Bru and contain this 10-nt sequence. This suggests that the sequential handoff of mRNAs between Pum and Bru is broadly utilized to control translation of maternal RNAs. We propose that this handoff from one set of *trans*-acting factors utilizing a poly(A)-shortening mechanism to another set of *trans*-acting factors that utilizes a cap-dependent mechanism is required to protect mRNAs post-differentiation and prime them for translation during embryogenesis.

RESULTS

Pgc Is Translationally Regulated via Its UTRs

During oogenesis, Pgc is expressed in CBs, where it promotes timely differentiation (Figure 1C) (Flora et al., 2018). To assess if this temporal specificity of Pgc protein production is due to transcriptional or translational regulation, we carried out fluorescent *in situ* hybridization (FISH) for *pgc* in wild-type ovaries and for GFP in ovaries of flies carrying a reporter for Pgc (Flora et al., 2018). *pgc* transcription in the GSCs was difficult to discern because of the low resolution of FISH in the germarium; however, we did detect *pgc* mRNA in all later-differentiated stages (Figures 1D and S1A–S1C). To assess *pgc* mRNA expression in the GSCs through an alternate method, we overexpressed the self-renewal signaling receptor, thick veins receptor (TKV), to enrich for GSCs and then sequenced their transcriptome (Xie and Spradling, 1998). We detected 88 transcripts per million (TPM) of *pgc*, indicating that the mRNA is transcribed in the GSCs (Figures 1E and S1D). To further substantiate that the *pgc* promoter is active in the GSCs, we created a reporter construct in which the *pgc* promoter drives the expression of GFP flanked by the *nos* 5′ UTR and *K10* 3′ UTR, which are not translationally silenced during oogenesis (Figure 1F) (Serano et al., 1994; Gavis and Lehmann, 1992, 1994). We observed GFP expression throughout oogenesis, including in the GSCs. This suggests that the maternal *pgc* mRNA is transcribed from the GSCs onward throughout oogenesis and is under strict translational regulation pre- and post-differentiation (Rangan et al., 2008).

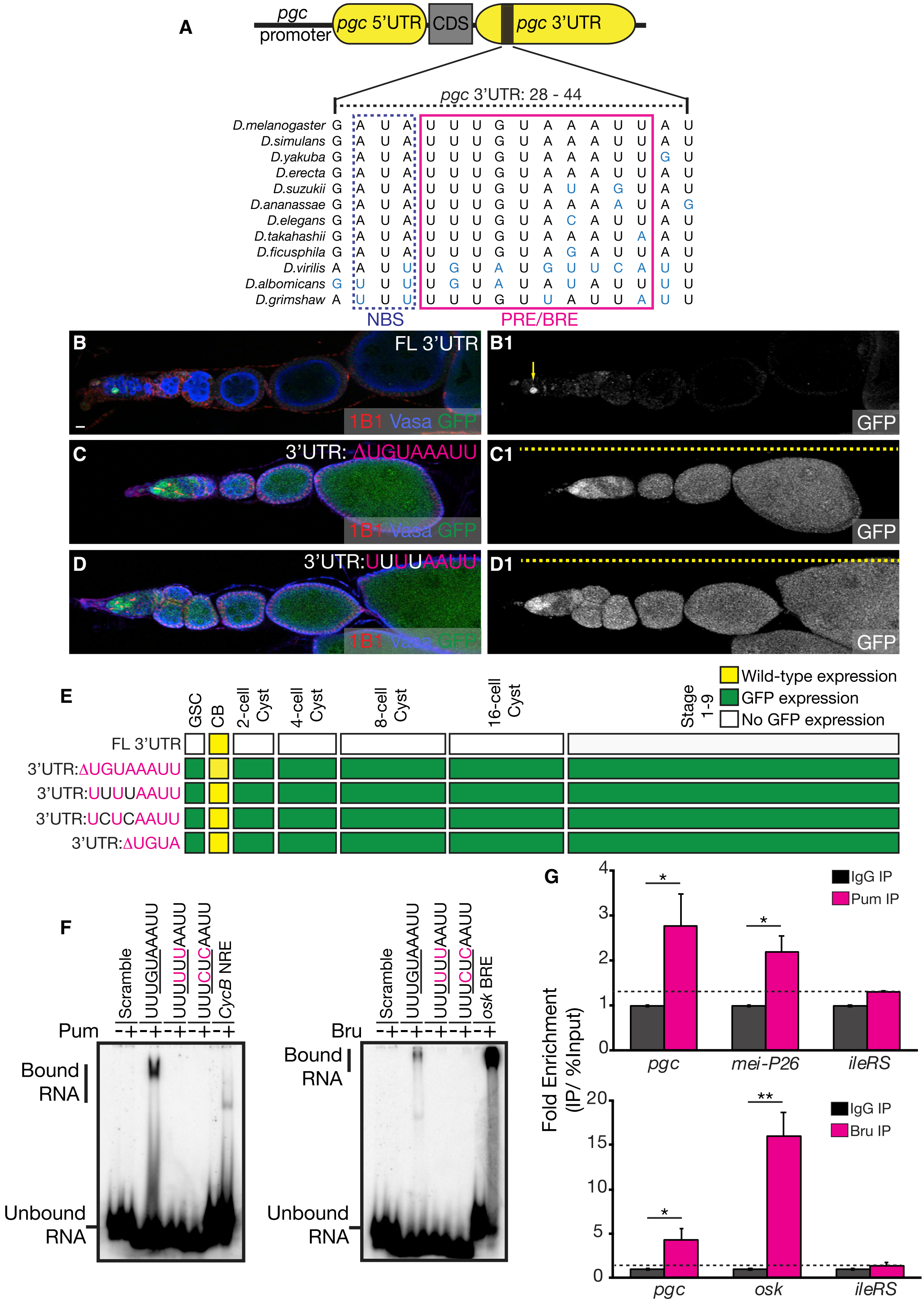

(legend on next page)

The 5′ UTR and 3′ UTR of an mRNA are commonly recognized by sequence-specific RBPs to regulate translation (Wilkie et al., 2003). We wanted to test the potential role of both the 5′ UTR and 3′ UTR of *pgc* in repressing translation in the GSCs. *pgc* mRNA has two annotated 5′ UTRs; to determine which one was expressed in the GSCs, we designed primers that distinguish these two forms. We carried out PCR on RNA enriched from GSCs by overexpressing TKV, and for CBs, by using a mutation for differentiation factor, *bag-of-marbles* (*bam*) (Xie and Spradling, 1998; McKearin and Ohlstein, 1995). We found that only the short form was expressed in the GSCs and CBs (Figure S1E). To determine if this short *pgc* 5′ UTR is required for translational regulation of *pgc*, we swapped it with the *nos* 5′ UTR in a GFP reporter construct that still retained the *pgc* 3′ UTR and the *pgc* promoter. We found that the absence of the *pgc* 5′ UTR results in upregulation of GFP protein expression in the GSCs, but not in later stages (Figure 1G). Our results indicate that in GSCs, the *pgc* 5′ UTR is required for translational regulation, while the 3′ UTR is not sufficient (Figure 1G). In differentiated stages, the 3′ UTR alone is sufficient to mediate translational regulation (Figure 1G). To test if the 5′ UTR is sufficient for translational regulation in GSCs, we created a construct with the *pgc* 5′ UTR and non-repressed *K10* and *tubulin* (*tub*) 3′ UTRs flanking GFP under the control of the *pgc* promoter (Figures 1H and S1F). GFP was expressed in the GSCs as well as in later-differentiating stages and egg chambers, demonstrating that the 5′ UTR alone is not sufficient for translational regulation (Figures 1H and S1F). Taken together, we conclude that both the *pgc* 5′ UTR and 3′ UTR are required for translational control pre-differentiation in the GSCs and that the 3′ UTR alone is sufficient post-differentiation in the cysts and egg chambers.

A *cis*-Element in the *pgc* 3′ UTR that Binds Both Pum and Bru Is Required for Translational Control throughout Oogenesis

We predicted that *cis*-acting sequences in either the 5′ or 3′ UTRs of *pgc* could regulate translation during oogenesis by recruiting *trans*-acting factors. To identify these sequences, we carried out a phylogenetic analysis of the *pgc* 5′ and 3′ UTR in Drosophilids separated by 40 million years of evolution and discovered several regions of conservation in the 3′ UTR (Figure S1G). We could not identify unique conserved regions in the *pgc* 5′ UTR, as the sequence overlaps with the coding region of *type III alcohol dehydrogenase* (*T3dh*). We also used algorithms that search for RBP-binding sequences and did not find any in the short form 5′ UTR of *pgc* (Bailey et al., 2009). In the 3′ UTR, a conserved 10-nt sequence, UUUGUAAAUU, stood out (Figures 2A and S1G). This sequence closely matches the sequences that have been previously described as the Pumilio response element (PRE), which is part of the Nanos response element (NRE) in *hunchback* and *Cyclin B* (*CycB*), respectively (Weidmann et al., 2016; Murata and Wharton, 1995; Kadyrova et al., 2007). PREs are known to bind Pum, which then recruits Nos, to bind to the Nanos-binding sequence (NBS), resulting in translational regulation of RNAs (Figure 2A) (Murata and Wharton, 1995; Kadyrova et al., 2007). This sequence in the *pgc* 3′ UTR can also bind another conserved RBP, Bru. Pum binds to the UGUA motif, while Bru binds to a uU$^{G/A}$U$^{G/A}$U$^{G/A}$Uu motif, which is described as the Bruno response element (BRE) (Kim-Ha et al., 1995; Wharton and Struhl, 1991).

We asked if this conserved 10-nt sequence that is predicted to bind two RBPs can regulate *pgc* translation. To test this, we generated a reporter construct that deleted 8 nt of the conserved sequence including the UGUA motif that is known to bind Pum and the uU$^{G/A}$U$^{G/A}$ motif that binds Bru. This resulted in an upregulation of translation throughout oogenesis (Figures 2B, 2C, 2E, and S1J). We also generated three transgenes in which we mutated the core UGUA motif to UUUU or UCUC and also deleted the core UGUA motif. We found that all these changes resulted in loss of translational control (Figures 2D, 2E, and S1H–S1J). To test if this 10-nt PRE and/or BRE was sufficient for translation regulation, we generated a reporter construct where we inserted the conserved sequence into the *tub* 3′ UTR (*tub* 3′UTR: NBS + PRE and/or BRE), fused it to GFP and *pgc* 5′ UTR, and drove it under the control of *pgc* promoter. We found that the inclusion of this sequence in the 3′ UTR of *tub* is sufficient to repress GFP translation throughout oogenesis, but it is not sufficient for GFP expression in the pre-CB (Figures S2A–S2C). Thus, we conclude that the conserved 10-nt sequence in the *pgc* 3′ UTR that is predicted to bind Pum and Bru is required and sufficient for translation repression of *pgc* during oogenesis.

To determine if the conserved sequence binds Pum and Bru as predicted, we purified the recombinant RNA-binding domain of Pum and full-length Bru and carried out electrophoresis mobility

Figure 2. A *cis*-Element in the *pgc* 3′ UTR that Binds Pum and Bru Is Required for Translational Control throughout Oogenesis

(A) The NBS and PRE and/or BRE sequence identified in the *pgc* 3′ UTR is conserved in 12 species of Drosophilids.

(B) An ovariole of a *pgcGFP* fly stained with 1B1 (red), Vasa (blue), and GFP (green) showing that GFP expression is restricted to the pre-CB (arrow in B1).

(C) An ovariole of a *pgcGFP* reporter that lacks the PRE and/or BRE sequence in the 3′ UTR stained with 1B1 (red), Vasa (blue), and GFP (green). GFP regulation was lost throughout oogenesis (dashed line in C1).

(D) An ovariole of a *pgcGFP* reporter in which the PRE and/or BRE core UGUA motif was mutated stained with 1B1 (red), Vasa (blue), and GFP (green). GFP regulation was lost throughout oogenesis (dashed line in D1).

(E) A developmental profile of GFP expression in different stages of oogenesis of transgenes in which the PRE and/or BRE sequence was either deleted or mutated.

(F) EMSAs show that purified Pum and Bru proteins bind to the PRE and/or BRE of the *pgc* 3′ UTR, the NRE of the *CycB* 3′ UTR, and the BRE of the *osk* 3′ UTR, respectively.

(G) qPCR of *pgc*, *mei-P26* (positive control), and *ileRS* (negative control) carried out on RNA samples extracted after an IP with Pum antibody (top). qPCR of *pgc*, *osk* (positive control), and *ileRS* (negative control) carried out on RNA samples extracted after an IP with Bru antibody (bottom). RIP-qPCR graphs represent an average generated from three independent biological samples. The error bars represent SE. A Student's t test analysis was performed. * and ** indicate a p value < 0.05 and < 0.005, respectively.

Scale bar, 10 μm. See also Figures S1 and S2.

OPEN ACCESS
CellPress

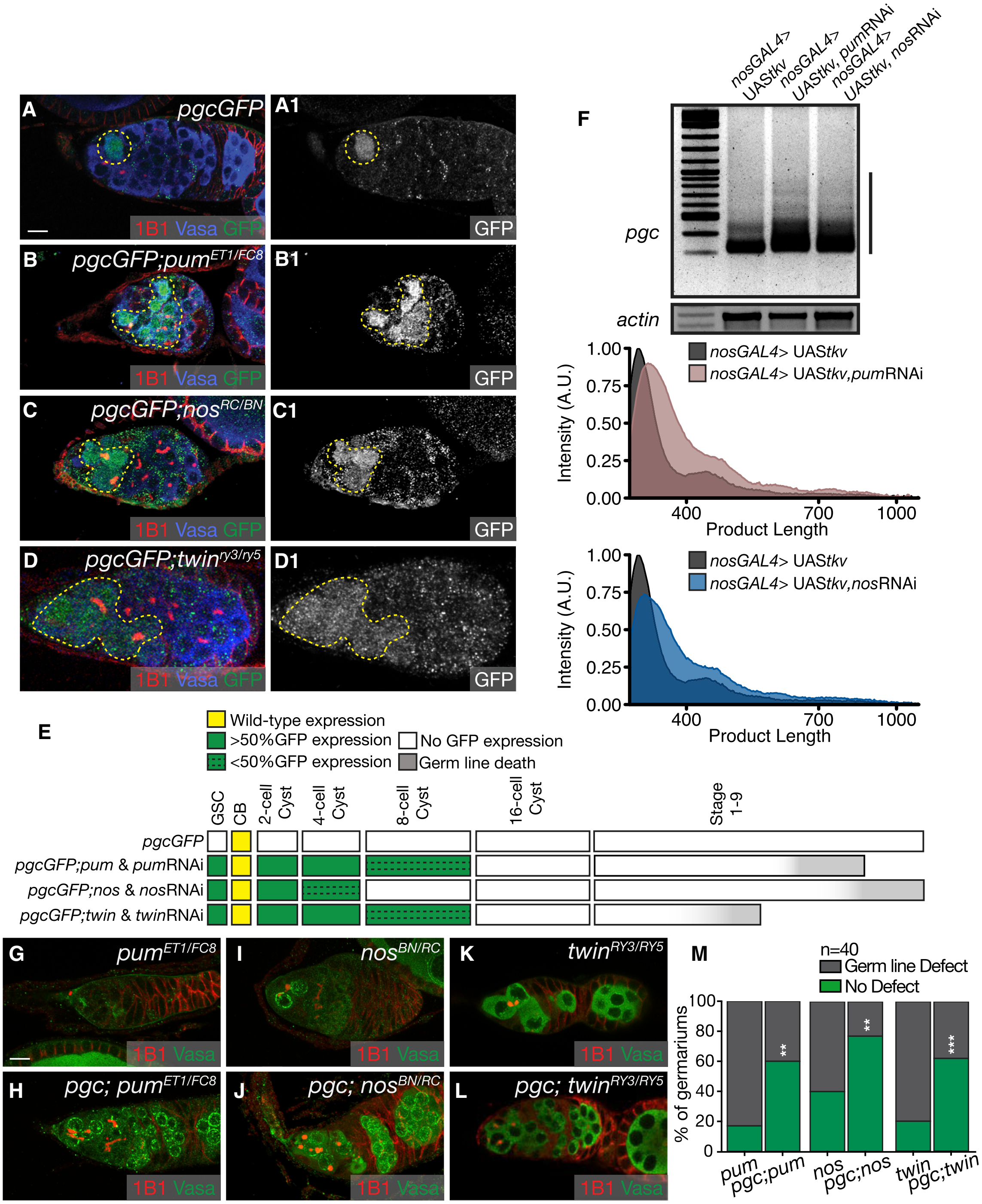

Figure 3. Pum and Its Cofactor, Nos, Regulate Pgc Translation in the GSCs and Early-Differentiating Cysts

(A) A germarium of a *pgcGFP* ovary stained with 1B1 (red), Vasa (blue), and GFP (green) shows expression of GFP only in the pre-CB (dashed circle).

(B) A germarium of a *pgcGFP; pum*$^{ET1/FC8}$ ovary stained with 1B1 (red), Vasa (blue), and GFP (green) shows aberrant GFP expression from GSCs to the 8-cell cyst (100% from GSC to 4-cell cyst, 32% in 8-cell cyst, n = 25) (dashed outline).

(C) A germarium of a *pgcGFP; nos*$^{RC/BN}$ ovary stained with 1B1 (red), Vasa (blue), and GFP (green) shows aberrant GFP expression from GSCs to the 4-cell cyst (100% from GSCs to 2-cell cyst, 13% in 4-cell cyst, n = 25) (dashed outline).

(D) A germarium of a *pgcGFP; twin*$^{RY3/RY5}$ ovary stained with 1B1 (red), Vasa (blue), and GFP (green) shows aberrant GFP expression from GSCs to the 8-cell cyst (100% from GSC to 4-cell cyst, 40% in 8-cell cyst, n = 25) (dashed outline). The GFP channel is shown in A1–D1.

(E) A developmental profile of GFP expression when Pum, Nos, and Twin are depleted in the germline.

(F) PAT assay of *pgc* poly(A)-tail length in GSC tumors and in GSC tumors lacking Pum and Nos.

(legend continued on next page)

shift assay (EMSA) experiments (Figure S2D) (Chekulaeva et al., 2006; Weidmann et al., 2016). As positive controls, we utilized the NRE in *CycB* and the BRE in *Oskar's* (*osk*) 3′ UTR and demonstrated that our recombinant Pum and Bru bound the NRE and BRE, respectively (Figure 2F) (Kim-Ha et al., 1995; Kadyrova et al., 2007). Both Pum and Bru also bound the PRE and/or BRE in the 3′ UTR of *pgc*. This binding was lost when the core UGUA sequence was mutated to UCUC or UUUU (Figure 2F). To test if Pum and Bru also bind to *pgc* mRNA *in vivo*, we performed an RNA immunoprecipitation (RIP)-qPCR experiment with anti-immunoglobulin G (anti-IgG), anti-Pum, and anti-Bru antibody in lysates from wild-type ovaries. We observed that along with known RNA targets, *mei-P26* for Pum and *osk* for Bru, *pgc* RNA was significantly enriched in both Pum and Bru pull-downs relative to non-specific IgG pull downs. There was no significant enrichment of a non-target RNA, *isoleucyl-tRNA synthetase* (*ileRS*), in either of these pull-downs (Figures 2G and S2E). Thus, we conclude that Pum and Bru bind to the 10-nt PRE and/or BRE of *pgc* 3′ UTR *in vitro* and to *pgc* mRNA *in vivo*.

Pum and Its Cofactor, Nos, Regulate Pgc Translation in the GSCs and Early-Differentiating Cysts

We asked if *pgc* was translationally regulated by Pum and Bru during oogenesis, and in particular, given their inverse expression patterns, if they might each govern distinct phases. Pum is expressed from GSCs to the 8-cell cyst stage and is attenuated from the 16-cell cyst onward (Figures S2F–S2F2′) (Forbes and Lehmann, 1998; Carreira-Rosario et al., 2016). Bru levels are low from GSCs to the 8-cell cyst stage but are high in the 16-cell cyst stage and throughout later oogenesis (Figures S2F–S2F2′) (Webster et al., 1997; Sugimura and Lilly, 2006; Xin et al., 2013). Thus, we hypothesized that Pum may regulate *pgc* translation until the 8-cell cyst and Bru thereafter. We first focused on Pum and its potential role in regulating *pgc* translation during early oogenesis. Pum requires co-factors to regulate translation and can use distinct partners and multiple mechanisms. Pum is known to recruit Nos and Twin, a deadenylase, to NRE-containing 3′ UTRs to induce poly(A)-tail shortening in *Drosophila* embryonic germ cells (Sonoda and Wharton, 1999; Kadyrova et al., 2007). During oogenesis, Twin is ubiquitously expressed (Temme et al., 2010; Joly et al., 2013) and Nos protein is present in all stages, except for in the pre-CB where Pgc is expressed (Figures S3A–S3B1) (Forbes and Lehmann, 1998; Li et al., 2009). We therefore hypothesized that Pum might be regulating Pgc expression with Nos and Twin only until the cyst stages, during which time a drop in Nos expression in the pre-CBs would allow for Pgc expression.

To test this hypothesis, we separately assayed for PgcGFP expression in *pum*, *nos*, and *twin* mutants. We observed that in the absence of each of these genes, the reporter was ectopically expressed in the GSCs, as marked by pMAD, and in 2- and 4-cell cysts (Figures 3A–3D1 and S3C–S3F). Ectopic expression in the GSCs was also observed upon germline depletion of *pum*, *nos*, and *twin* via RNAi (Figures S3G–S3I and S3N). We confirmed that Pum RNAi depleted Pum in the germline (Figures S3J–S3K1). Twin is a deadenylase and is part of the CCR4-Not complex (Morris et al., 2005; Temme et al., 2010; Fu et al., 2015). To determine if other members of this complex were involved in regulating *pgc* translation, we depleted Pop2 and Not1 in the germline using RNAi and assayed for GFP expression. Compared to *pgcGFP*, depletion of Pop2 and Not1 resulted in ectopic expression of the reporter from the GSCs to the 4-cell cysts, consistent with what we observed in the *nos*, *pum*, and *twin* mutants (Figures S3L–S3N). We also observed that loss of *pum* and *twin* results in an elevated GFP expression in the 8-cell cyst. Differences of ectopic *pgcGFP* reporter expression is not due to *nosGAL4* activity in the germline (Figures S3O–S3P1). We generated a developmental profile to show the temporal loss of translational regulation of GFP at each stage of development in *pum*, *nos*, and *twin* when compared to control *pgcGFP* ovarioles (Figure 3E). Taken together, we can conclude that *pgc* is regulated by Nos, Pum, and Twin from GSCs to the 4-cell cyst stage via the CCR4-Not complex. In the pre-CB, when Nos is absent, Pgc is expressed even though Pum and Twin proteins are still present. This suggests that Pum and Twin alone are not sufficient for regulating *pgc* in the pre-CB and require the presence of their co-regulator Nos.

To test if Pum and Nos control translation of *pgc* mRNA by shortening poly(A)-tail length, we utilized the poly(A)-tail-length (PAT) assay (Sallés and Strickland, 1999). We performed this assay on RNA extracted from GSC-enriched tumors and GSC tumors depleted of Nos and Pum to eliminate the stage of oogenesis in which *pgc* is translationally repressed (Figures S4A–S4C1). In the absence of these RBPs, we detected an increase in the length of the poly(A)-tail compared to the control (Figure 3F). Together, these observations suggest that Pum, Nos, and Twin are recruited to *pgc's* 3′ UTR to suppress its translation in the GSCs by a mechanism that involves shortening its poly(A)-tail.

We next asked if this regulation of *pgc* by Pum, Nos, and Twin is biologically meaningful. Loss of *pum* and *nos* results in failure to maintain GSCs, and this defect is thought to be the result of dysregulation of differentiation-promoting mRNAs in the GSCs (Forbes and Lehmann, 1998; Wang and Lin, 2005). We have previously shown that *pgc* promotes timely differentiation in the pre-CBs (Flora et al., 2018). Thus, we hypothesized that in *nos*, *pum*, and *twin* mutants, Pgc is upregulated in the GSCs, forcing premature differentiation. To test this hypothesis, we made double mutants of *pgc* with *nos*, *pum*, and *twin*, respectively. Lowering *pgc* levels in all three mutants rescued germline defects (Figures 3G–3M). Together, our results suggest that Pgc is translationally repressed by Pum, Nos, and Twin in the GSCs to ensure appropriate GSC self-renewal and maintenance.

(G, I, and K) Germaria of $pum^{ET1/FC8}$ (G), $nos^{RC/BN}$ (I), and $twin^{RY3/RY5}$ (K) mutants stained with 1B1 (red) and, Vasa (green).
(H, J, and L) Germaria of *pgc*; $pum^{ET1/FC8}$ (H) *pgc*; $nos^{RC/BN}$ (J) and *pgc*; $twin^{RY3/RY5}$ (L) double mutants stained with 1B1 (red) and Vasa (green).
(M) A graphical representation of the rescue experiment (n = 40). A population proportion z-test was performed. ** and *** indicate a p value < 0.005 and < 0.0005, respectively.
Scale bars, 10 μm. See also Figures S3 and S4.

OPEN
ACCESS
CellPress

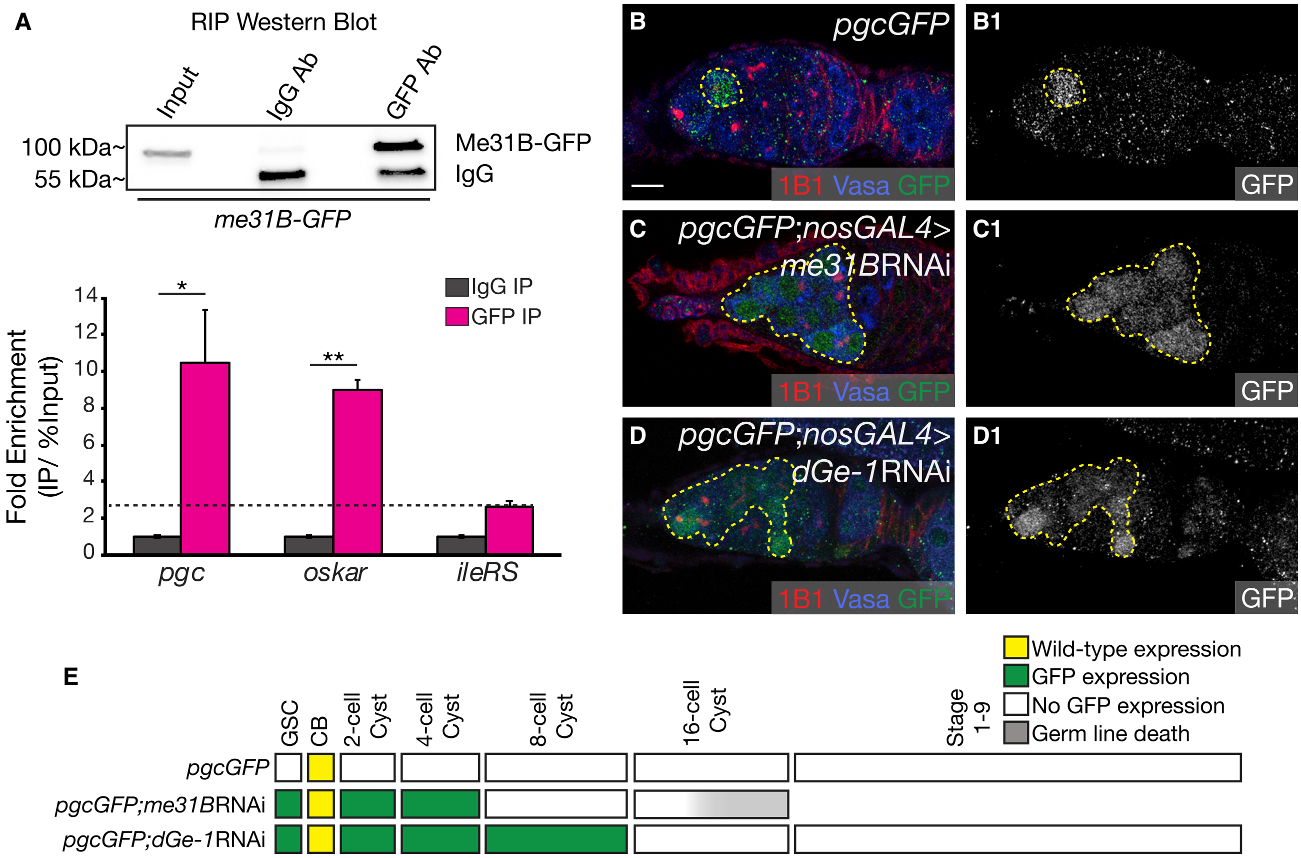

Figure 4. Me31B Cooperates with the Decapping Protein dGe-1 and the *pgc* 5′ UTR to Mediate Repression in GSCs and Early-Differentiating Cysts

(A) Western blot shows pull-down of GFP from *me31BGFP*-trap fly ovary lysates (top). qPCR of *pgc*, *mei-P26* (positive control), and *ileRS* (negative control) carried out on RNA samples extracted after the IP (bottom). The graph represents an average generated from three independent biological samples. The error bars represent SE. A Student's t test analysis was performed. * and ** indicate a p value < 0.05 and < 0.005, respectively.

(B) A germarium of a *pgcGFP* ovary stained with 1B1 (red), Vasa (blue), and GFP (green) shows expression of GFP only in the pre-CB (dashed circle).

(C) A germarium of a *pgcGFP*; *nosGAL4>me31B*RNAi ovary stained with 1B1 (red), Vasa (blue), and GFP (green) shows aberrant GFP expression in GSCs to the 4-cell cyst (100%, n = 20) (dashed outline).

(D) A germarium of *pgcGFP*; *nosGAL4>dGe-1*RNAi stained with 1B1 (red), Vasa (blue), and GFP (green) shows aberrant GFP expression in GSCs to the 8-cell cyst stages (100%, n = 20) (dashed outline). The GFP channel is shown in B1–D1.

(E) A developmental profile of GFP expression when Me31B and dGe-1 are depleted in the germline.

Scale bar, 10 μm. See also Figure S4.

Me31B Cooperates with the Decapping Protein dGe-1 and the *pgc* 5′ UTR to Mediate Repression in GSCs and Early-Differentiating Cysts

Our results suggest that Pum, Nos, and Twin regulate *pgc* translation via a conserved sequence in the *pgc* 3′ UTR. However, we also found a requirement for the *pgc* 5′ UTR in the regulation of *pgc* in undifferentiated cells (Figure 1G). Does the 5′ UTR and 3′ UTR of *pgc* cooperate to mediate repression? It has been shown that recruitment of the CCR4-Not complex also facilitates the recruitment of the decapping complex to the 5′ UTR of mRNAs (Meyer et al., 2004; Garneau et al., 2007; Behm-Ansmant et al., 2006) and that these two complexes at the 5′ UTR and 3′ UTR can be bridged by an RNA helicase, DDX6, or maternal expression at 31B (Me31B) (Ozgur et al., 2015; Nakamura et al., 2001). This allows "masking" of the mRNAs, making them inaccessible to the ribosome. We therefore hypothesized that Pum, Nos, and Twin at the *pgc* 3′ UTR could recruit decapping complex members, such as EDC4 or *Drosophila* Ge-1 (dGe-1), to the cap at the 5′ UTR to promote translational repression by masking through the bridging action of Me31B (Fan et al., 2011; Eulalio et al., 2007).

To test this model, we first asked if Me31B associates with *pgc* mRNA. We used wild-type ovaries from a Me31B protein-GFP trap construct and carried out a RIP-qPCR experiment with both anti-GFP and anti-IgG antibodies. We found that there was a significant enrichment of *pgc* mRNA bound to Me31B-GFP protein comparable to those of the positive control, *osk* mRNA (Figure 4A) (Nakamura et al., 2001) and no significant enrichment of a non-target RNA, *ileRS*. Next, we assayed for *pgc*GFP expression upon germline depletion of *me31B* and

found a loss of GFP repression from the GSC to the 4-cell cyst (Figures 4B–4C1 and 4E). *me31B* RNAi results in depletion of Me31B (Figures S4D–S4E1). We also observed ectopic *pgcGFP* reporter expression from the GSC to the 8-cell cyst stage in the presence of the *dGe-1* RNAi (Figures 4D, 4E, and S4F). Our results suggest that *pgc* 5′ and 3′ UTRs together with Me31B and proteins of the decapping complex such as dGe-1 regulate its translation.

Pum and Its Cofactor, Brat, Regulate Pgc Translation in the 4- to 16-Cell Cysts

Pum can also mediate translational repression via an alternate mechanism by recruiting Brat (Sonoda and Wharton, 2001; Muraro et al., 2008; Olesnicky et al., 2012; Harris et al., 2011). Brat engages the cap-binding protein d4EHP, which competes with the cap-binding protein eIF4E, to prevent translational initiation (Cho et al., 2005). Pum is present from the GSCs until the 8-cell cyst and is attenuated from the 16-cell cyst onward, while Brat is expressed only after the CB differentiates and persists throughout all later cyst stages (Carreira-Rosario et al., 2016; Harris et al., 2011). To test if Pum regulates *pgc* via Brat, we assayed for *pgcGFP* expression in the pum^{680} mutant, a separation-of-function mutant that disrupts the interaction between Pum and Brat without affecting the interaction between Pum and Nos (Wharton et al., 1998; Sonoda and Wharton 1999). We found that in pum^{680} mutants, there was ectopic *pgcGFP* reporter expression from the 4- to 16-cell cyst, but not in the earlier stages (Figures 5A–5B1 and S5A). This observation suggested that Pum may be interacting with Brat and its partner, d4EHP, to repress *pgc* translation in the differentiating cysts. To test this, we depleted *brat* and *d4EHP* in the germline using RNAi. We observed that loss of Brat and d4EHP also results in ectopic expression of GFP from 4- to 16-cell cyst, but not in the earlier stages (Figures 5C–5D1 and S5A). Although we do not see an upregulation of reporter expression in the 16-cell cyst in a *pum* mutant and a *pum*RNAi ovary (Figures 3B and S3G), we do see ectopic expression of GFP in the 16-cell cyst when Brat and d4EHP are depleted in the germline. Brat can act independent of d4EHP during oogenesis and independent of Pum during embryogenesis (Harris et al., 2011; Laver et al., 2015). We do not think Brat acts independent of either Pum or d4EHP to regulate *pgc* during oogenesis, as we see ectopic reporter expression from the 4- to 16-cell cyst when the Pum-Brat interaction is specifically perturbed in a pum^{680} mutant and upon loss of d4EHP. We think that the reason why *pum* mutant alleles and RNAi lines repress *pgc* in the 16-cell cysts could be due to their hypomorphic nature. A developmental profile of GFP expression in *pgcGFP, pgcGFP;* pum^{680}*, pgcGFP; nosGAL4* > *brat*RNAi and *pgcGFP; nosGAL4* > *d4EHP*RNAi shows that compared to the control, loss of Brat and d4EHP results in the loss of *pgcGFP* regulation restricted to the 4- and 16-cell cysts (Figure 5E). We conclude that Pum, Brat, and d4EHP regulate Pgc translation in the 4- to 16-cell cysts. To determine whether Pum-Brat complex affects the poly(A)-tail length of *pgc*, we performed a PAT assay on *pgc* RNA in pum^{680} mutants and germline depletions of *brat* and *d4EHP.* We observed no significant change in these mutants (Figure S5B). These results suggest that Pum switches not only binding partners but also the mode of regulation from a poly(A)-tail-dependent mechanism to a cap-dependent mechanism to regulate *pgc* translation pre- and post-differentiation, respectively.

Bru and Cup Regulate Pgc Translation in the Later Stages of Oogenesis

After differentiation, levels of Pum diminish and levels of Bru increase (Figures S2F–S2F2′). We have shown that Bru binds to the 10-nt conserved sequence in the 3′ UTR that is required for *pgc* translational control throughout oogenesis (Figures 2C and 2F). Therefore, we asked if Bru and its binding partner, Cup, can repress Pgc translation post-differentiation (Nakamura et al., 2004; Chekulaeva et al., 2006; Kim et al., 2015b). Assaying for the *pgc* reporter in both *bru* mutants and germline depletion of Bru via RNAi, we found that translation was de-repressed primarily from the 16-cell cyst stage onward (Figures 6A–6B1, S6A, and S6B). We confirmed that *bru*RNAi depleted Bru in the germline (Figures S6C–S6D1). To determine if Bru recruits Cup to mediate this regulation, we depleted *cup* in the germline via RNAi and observed similar ectopic expression of GFP from the 16-cell cyst stage (Figure 6C). A developmental profile of GFP expression in *pgcGFP; nosGAL4, pgcGFP; nosGAL4* > *bru*RNAi and *pgcGFP; nosGAL4* > *cup*RNAi shows that compared to the control, loss of *bru* and *cup* results in loss of *pgcGFP* regulation primarily from the 16-cell cyst stage onward (Figure 6D). To test if Bru and Cup's mode of regulation affected the poly(A)-tail length of *pgc*, we performed a PAT assay on *pgc* RNA in germline depletion of Bru and Cup. We observed that Bru and Cup depletion results in an increase of *pgc* poly(A)-tail length with depletion of *bru* showing a more dramatic change (Figure 6E). As Bru can act independent of Cup to form RNA oligomers that "mask" transcripts from the translation initiation machinery (Chekulaeva et al., 2006), we think that in the absence of Cup, Bru can independently regulate a subset of *pgc* mRNAs. As loss of components of the CCR4-Not complex does not show loss of translational control in later stages and poly(A)-tail length increase has been shown as directly correlated to increased translational efficiency (Eichhorn et al., 2016; Sachs and Wahle, 1993), we favor the model that *pgc* is regulated in the differentiated stages by Bru and its binding partner, Cup, via a cap-dependent mechanism that restricts access to both cap and poly-adenylation machinery.

A Class of Germline RNAs Are Similarly Regulated by Both Pum and Bru

Our results show that the conserved RBPs Pum and Bru can recognize and bind the same *cis*-element in the *pgc* 3′ UTR to mediate repression throughout oogenesis. We wondered if this mechanism could be applicable for regulation of other maternally deposited mRNAs. To address this, we carried out a polysome-sequencing (Poly-seq) experiment to calculate the translational efficiency (TE) of transcripts (Kronja et al., 2014). We utilized this method to identify transcripts that are actively translated in the ovaries of *nosGAL4*>*pum*RNAi and *nosGAL4*>*bru*RNAi flies when compared to young *nosGAL4* flies. We used young *nosGAL4* ovaries as controls because they do not have mature later stages (stage 10 and onward) comparable to germline depletion of both Pum and Bru. We conducted RNA sequencing (RNA-seq) of transcripts extracted from the polysome fractions

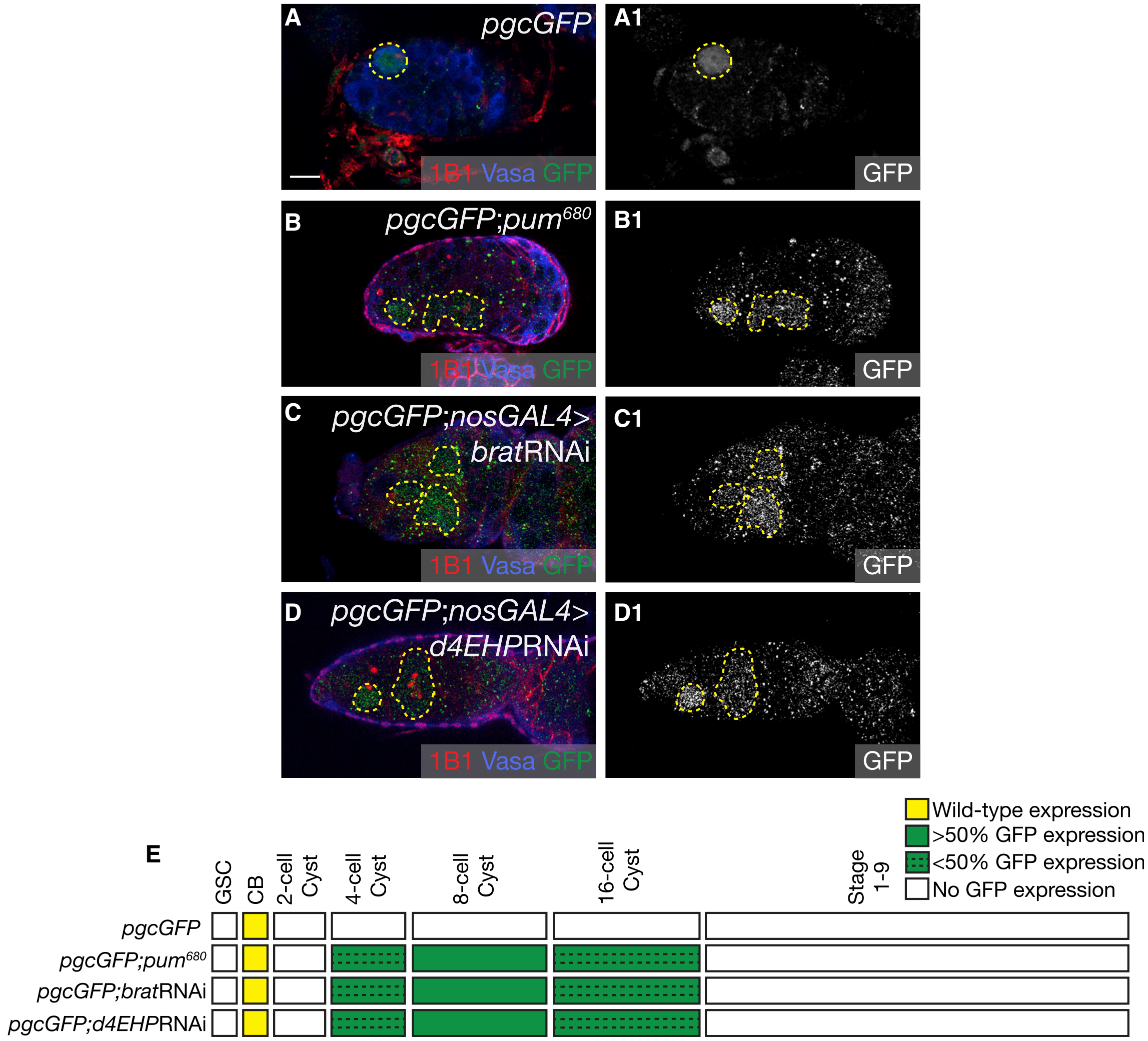

Figure 5. Pum and Its Cofactor, Brat, Regulate Pgc Translation in 4- to 16-Cell Cysts

(A) A germarium of a *pgcGFP* ovary stained with 1B1 (red), Vasa (blue), and GFP (green) shows expression of GFP in the pre-CB (dashed circle).

(B) A germarium of a *pgcGFP*; pum^{680} ovary stained with 1B1 (red), Vasa (blue), and GFP (green) shows aberrant expression of GFP in the differentiating cysts (25% in the 4-cell cyst, 75% in the 8-cells cyst, and 10% in the 16-cell cyst, n = 20) (dashed outline).

(C) A germarium of a *pgcGFP*; *nosGAL4>brat*RNAi ovary stained with 1B1 (red), Vasa (blue), and GFP (green) shows aberrant expression of GFP in the differentiating cysts (38% in the 4-cell cyst, 54% in the 8-cells cyst, and 18% in the 16-cell cysts, n = 30) (dashed outline).

(D) A germarium of a *pgcGFP*; *nosGAL4>d4EHP*RNAi ovary stained with 1B1 (red), Vasa (blue), and GFP (green) shows aberrant expression of GFP in the differentiating cysts (34% in the 4-cell cyst, 62% in the 8-cells cyst, and 15% in the 16-cell cyst, n = 32) (dashed outline). The GFP channel is shown in A1–D1.

(E) A developmental profile of GFP expression when the Pum-Brat interaction is ablated and Brat and d4EHP are depleted in the germline.

Scale bar, 10 μm. See also Figure S5.

(Figure S7A). We found that when Pum and Bru are depleted in the germline, 1,081 and 908 transcripts have higher TE, respectively, than in the control (Figures 7A–7C; Tables S1 and S2). 436 of these transcripts display an increase in TE when either *pum* or *bru* is depleted, suggesting that these targets may be co-regulated (Figure 7C; Table S3). 212 of the 436 shared transcripts contained a sequence similar to the 10-nt PRE and/or BRE sequence identified in the *pgc* 3′ UTR (Figure S7B; Table S4). 368 of the 436 transcripts and 179 of the 212 transcripts are maternally provided mRNAs that are also present in mature eggs (Kronja et al., 2014). Gene Ontology analysis of the 212 shared targets show these genes are required for gastrulation and cell motility; processes mediated by maternally deposited RNAs and occurring prior to the maternal-to-zygotic transition of *Drosophila* embryogenesis (Figure 7D). One such gene identified to be co-regulated by Pum and Bru throughout oogenesis was *zelda*, a maternally provided mRNA that plays the role of master regulator during early *Drosophila* embryogenesis (Figures 7A and 7B) (Harrison et al., 2011; Nien et al., 2011; Liang et al., 2008). It is a transcription factor that is required to activate early-developmental somatic genes essential for cellularization, sex determination, and body patterning. We do not know if these

OPEN
ACCESS
CellPress

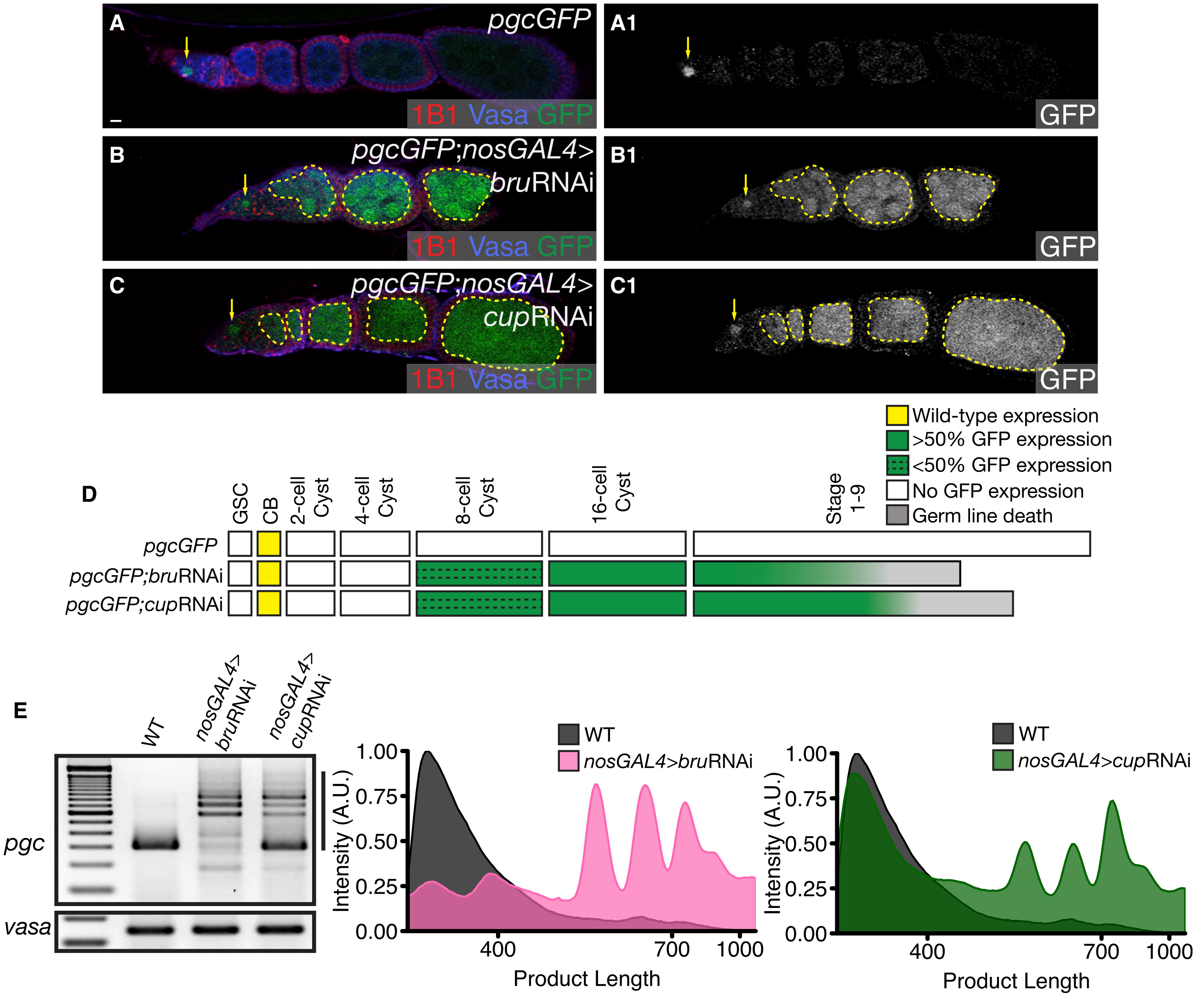

Figure 6. Bru and Cup Regulate Pgc Translation in the Later Stages of Oogenesis

(A) An ovariole of a *pgcGFP* ovary stained with 1B1 (red), Vasa (blue), and GFP (green) shows expression of GFP in the pre-CB (arrow).

(B) An ovariole of a *pgcGFP*; *nosGAL4>bru*RNAi ovary stained with 1B1 (red), Vasa (blue), and GFP (green) aberrant expression of GFP beyond the 16-cell cyst (12% from 8-cell cyst onward, 100% from 16-cell cyst onward, n = 25) (dashed outline).

(C) An ovariole of a *pgcGFP*; *nosGAL4>cup*RNAi ovary stained with 1B1 (red), Vasa (blue), and GFP (green) shows aberrant expression of GFP from the later cyst stages (20% from 8-cell cyst onward, 100% from 16-cell cyst onward, n = 30) (dashed outline). The GFP channel is shown in A1–C1.

(D) A developmental profile of GFP expression when Bru and Cup are depleted in the germline.

(E) PAT assay analysis of *pgc* poly(A)-tail length of *pgc* RNA when Bru and Cup are depleted in the germline.

Scale bars, 10 μm. See also Figure S6.

maternal mRNAs are expressed in the CBs, like *pgc*, or if additional translational regulatory mechanisms silence these mRNAs there. Taken together, our results demonstrate that key determinants for somatic and germline fate, such as *zelda* and *pgc*, respectively, are translationally suppressed by Pum and Bru to ensure their repression during oogenesis.

DISCUSSION

Here, we report that a maternal mRNA, *pgc*, is translationally repressed via different temporally restricted RBPs that use the same *cis*-acting sequence during oogenesis. We find that prior to differentiation, *pgc* 5′ and 3′ UTRs cooperate to regulate translation. In contrast, after differentiation, the 3′ UTR of *pgc* is necessary and sufficient for translational control. We find that a 10-nt conserved sequence in this 3′ UTR is essential for *pgc* regulation during the entirety of oogenesis. Surprisingly, two distinct RBPs whose expression is temporally restricted, Pum and Bru, both recognize and bind this conserved sequence to regulate translation. We find that regulation by these RBPs during oogenesis is not unique to *pgc* but that a large class of maternal mRNAs also lose translational control in the absence

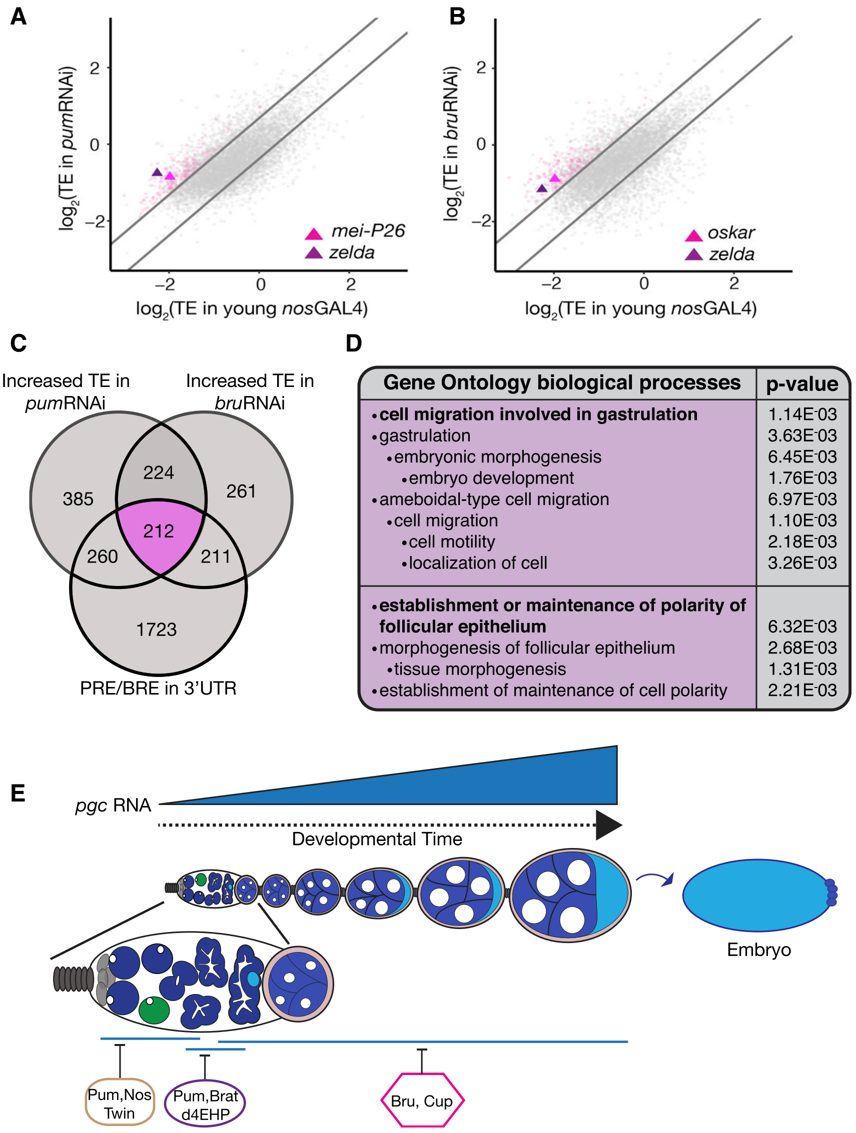

Figure 7. A Class of Germline RNAs Are Similarly Regulated by Both Pum and Bru

(A and B) A bi-plot representing the translational efficiencies (TEs) of expressed mRNAs in *nosGAL4>pum*RNAi (A) and *nosGAL4>bru*RNAi (B) versus young wild-type ovaries. The lines represent cutoffs, which are 1 SD above and below the median ratio. Pink points represent shared targets of Pum and Bru containing a PRE and/or BRE sequence.

(C) A Venn diagram showing the shared targets that have a higher TE upon the germline depletion of *pum* and *bru*. The targets in the pink set contain a PRE and/or BRE similar to that of *pgc's* in their 3′ UTR.

(D) Gene Ontology analysis of the 212 shared targets.

(E) A model accounting for the sequential regulation of *pgc* RNA by different RBPs throughout oogenesis.

See also Figure S7 and Tables S1–S4.

of both Pum and Bru. Our results indicate that 212 members of this class of mRNAs also share in their 3′ UTR a version of the 10-nt conserved sequence necessary for Pum and Bru regulation of *pgc*. These findings suggest that we have identified a broadly utilized mechanism that prevents the translation of specific mRNAs during oogenesis. The fact that some of these mRNAs affect gastrulation and developmental patterning argues that this mechanism evolved to prevent the translation of mRNAs that govern the key early steps of embryogenesis but could be deleterious if translated during oogenesis.

We find that a dynamic and diverse landscape of translational regulators has evolved to allow fine-scale control of maternal mRNAs. mRNAs can be regulated either by the CCR4-Not complex shortening the poly(A) tail or by the decapping machinery or other proteins that bind the cap interfering with cap recognition (Meyer et al., 2004; Garneau et al., 2007; Temme et al., 2014). CCR4-Not complex members as well as decapping machinery proteins are expressed continuously during *Drosophila* germline development and thus cannot mediate dynamic translational control on their own (Temme et al., 2010; Joly et al., 2013; Fan et al., 2011). However, carefully choreographed expression of specific RBPs that recognize and bind sequences in the UTRs recruit these regulatory proteins to target transcripts at different stages. Our studies show that Pum, whose expression is restricted to the earliest stages of oogenesis, associates with Nos to recruit the CCR4-Not complex to regulate *pgc* mRNA poly(A)-tails in the GSCs. After differentiation, Pum switches binding partners and complexes with Brat, a protein only expressed in the differentiating stages, and d4EHP, an adaptor protein that binds to the mRNA cap to mask *pgc* transcript from the translation initiation factors. As Pum levels diminish, this mode of regulation is handed over to Bru, which is robustly expressed from the 16-cell cyst onward, and its partner, Cup, which binds to eIF4E at the mRNA cap to mask *pgc* transcript from the translation initiation factors. Thus, we posit that by utilizing temporally restricted RBPs that bind the 3′ UTR at a single conserved sequence in a combinatorial fashion, the germline can sculpt differential expression of maternal mRNAs.

Why does *pgc* use the same sequence to bind the two *trans*-acting factors, Pum and Bru, as opposed to utilizing two distinct sequences? We observed that Pum recruits Brat, which complexes with d4EHP, to bind the cap and prevent the initiation machinery from accessing the mRNA. Bru accomplishes this by recruiting Cup, which binds eIF4E at the cap. If Pum and Bru are present at the same time, as in the 8- to 16-cell cyst stage, and are bound to different sequences, then they will recruit two proteins that compete to bind to the mRNA cap. In

the presence of Pum, its partner d4EHP can outcompete the cap partner eIF4E (Cho et al., 2005), which would make the handoff from Pum to Bru difficult. How then is repression of *pgc* mRNA seamlessly transitioned from one RBP to another? We also observed a temporal overlap in repression in the 4- and 8-cell cysts mediated by Pum with its two distinct partner complexes (Figure S7C). From the GSCs through 8-cell cyst stage, Pum partners with Nos, Twin, Me31B, and dGe-1 to repress *pgc,* while it partners with Brat and d4EHP to regulate *pgc* from the 4- through 16-cell cyst stages (Figures 7E and S7C). The overlap between Pum- and Bru-mediated repression occurs between the 8- and 16-cell cyst stages (Figures 7E and S7C). We hypothesize that to maintain seamless translational regulation during the 4- to 16-cell cyst stages, instead of competing for the cap, the RBPs compete to bind the same *cis*-element of their target mRNAs. When levels of one RBP diminish and those of another increase, the RBP present at a lower concentration could be displaced from its binding site on the mRNA, allowing for a smooth transition. Thus, we favor the idea that seamless transitions are mediated by overlapping *trans*-acting factor regimes and competition for the binding site.

pgc is transcribed continuously from the GSC stage onward and accumulates in the oocyte post differentiation. We find that there is a switch in mode of *pgc* regulation from a Twin (CCR4)-dependent mechanism mediated by Pum, which can destabilize mRNAs in the GSCs, to a Twin (CCR4)-independent mode mediated by Bru in the later-differentiated stages. Loss of Bru during oogenesis results in a dramatic increase in poly-adenylation of the *pgc* mRNA as well as translation of Pgc. This suggests that Bru-mediated regulation not only translationally represses *pgc* mRNA during oogenesis but also could maintain it in a state poised for poly-adenylation and translation. We also show that this mode of regulation is not unique to *pgc* and that there is a large set of maternally deposited germline mRNAs, including *zelda*, that seem to be regulated similarly. *zelda*, a transcription factor that activates the zygotic genome, is expressed at low levels in early embryos and increases as development proceeds concurrent with attenuation of Bru levels (Harrison et al., 2011; Nien et al., 2011; Webster et al., 1997). We hypothesize that post-differentiation, it is advantageous to switch the mode of translational regulation to a cap-dependent mechanism mediated by proteins such as Bru to prime these mRNAs to be translated during early embryonic development.

During mammalian development, maternally synthesized mRNAs are deposited into the egg to support embryonic development and need to be translationally regulated. Pum and CELF and/or Bruno-like proteins are both expressed in the mammalian germline and required for fertility (Kress et al., 2007; Mak et al., 2016). The mammalian homologs of Pum, PUMILIO 1 and 2 also bind to a sequence similar to the *Drosophila* NRE, and CELF1 and/or Bruno-like proteins bind to an “EDEN” sequence similar to *Drosophila* BREs (Wang et al., 2001; Vlasova et al., 2008; Jenkins et al., 2009). Pum and CELF and/or Bruno-like proteins are required not only in the germline but also for the development of other organs, including the CNS in mice (Spassov and Jurecic, 2003; Barreau et al., 2006; Wagnon et al., 2011; Zhang et al., 2017). Whether Pum and Bru function together on similar targets in the mammalian germline and nervous system as they do in the *Drosophila* ovary is not known. Our data suggest that such a handoff mechanism could be acting in these vertebrate systems as well.

STAR★METHODS

Detailed methods are provided in the online version of this paper and include the following:

- KEY RESOURCES TABLE
- CONTACT FOR REAGENT AND RESOURCE SHARING
- EXPERIMENTAL MODEL AND SUBJECT DETAILS
  - Fly strains
- METHOD DETAILS
  - Generation of transgenic fly strains
  - Immuno-fluorescence Staining
  - Fluorescent *in situ* hybridization (FISH)
  - Imaging
  - Western Blot
  - RNA Extraction
  - Real Time-PCR (RT-PCR) and quantitative Real Time-PCR (qRT-PCR)
  - Pumilio Protein Purification
  - Bruno Protein Purification
  - Electrophoretic mobility shift assays (EMSA)
  - Poly(A) tail length (PAT) Assay
  - RNA-Immuno-precipitation (RIP)-qPCR
  - RNA sequencing and sample library preparation
  - Polysome profiling and Polysome-seq
- QUANTIFICATION AND STATISTICAL ANALYSIS
  - Western Blot Analysis
  - Quantitative Real Time-PCR (qRT-PCR) analysis
  - RNA-Immuno-precipitation (RIP) qPCR analysis
  - Statistical Analysis
  - RNA-seq data analysis
  - Translation Efficiency (TE) Analysis
  - Gene Ontology (GO) Enrichment Analysis
- DATA AND SOFTWARE AVAILABILITY

SUPPLEMENTAL INFORMATION

Supplemental Information includes seven figures and five tables and can be found with this article online at https://doi.org/10.1016/j.celrep.2018.12.007.

ACKNOWLEDGMENTS

We would like to thank Dr. Buszczak (UT Southwestern) for anti-Nanos, Dr. Lilly (NIH), anti-Bruno (IP), and Dr. Lehmann (NYUMC) for anti-Pumilio and anti-Bruno (IF) antibodies; Dr. Salz (Case Western) and Dr. Nakamura (RIKEN) for the *pumGFP* and *me31BGFP* transgenes, respectively; Dr. Goldstrohm (University of Minnesota) for Pum protein expression vector; and Dr. Lehmann for GFP *in situ* probes. P.R. is funded by the NIH/NIGMS (grant RO1 1119484-1-68857) and the Pew Biomedical Scholars Program.

AUTHOR CONTRIBUTIONS

P.F., S.W.W.-D., E.T.M., R.J.P., and P.R. designed experiments and analyzed and interpreted data. P.F., S.W.W.-D., R.J.P., M.N., A.O., P.B., and D.P. performed experiments. P.F. and P.R. wrote the manuscript, which all authors edited and approved.

DECLARATION OF INTERESTS

The authors declare no competing interests.

Received: May 10, 2018
Revised: October 28, 2018
Accepted: November 30, 2018
Published: December 26, 2018

REFERENCES

Anderson, R.A., Fulton, N., Cowan, G., Coutts, S., and Saunders, P.T. (2007). Conserved and divergent patterns of expression of DAZL, VASA and OCT4 in the germ cells of the human fetal ovary and testis. BMC Dev. Biol. *7*, 136.

Arrizabalaga, G., and Lehmann, R. (1999). A selective screen reveals discrete functional domains in Drosophila Nanos. Genetics *153*, 1825–1838.

Astigarraga, S., Hofmeyer, K., Farajian, R., and Treisman, J.E. (2010). Three Drosophila liprins interact to control synapse formation. J. Neurosci. *30*, 15358–15368.

Bailey, T.L., Boden, M., Buske, F.A., Frith, M., Grant, C.E., Clementi, L., Ren, J., Li, W.W., and Noble, W.S. (2009). MEME SUITE: tools for motif discovery and searching. Nucleic Acids Res. *37*, W202–W208.

Barreau, C., Paillard, L., Méreau, A., and Osborne, H.B. (2006). Mammalian CELF/Bruno-like RNA-binding proteins: molecular characteristics and biological functions. Biochimie *88*, 515–525.

Behm-Ansmant, I., Rehwinkel, J., Doerks, T., Stark, A., Bork, P., and Izaurralde, E. (2006). mRNA degradation by miRNAs and GW182 requires both CCR4:NOT deadenylase and DCP1:DCP2 decapping complexes. Genes Dev. *20*, 1885–1898.

Carreira-Rosario, A., Bhargava, V., Hillebrand, J., Kollipara, R.K., Ramaswami, M., and Buszczak, M. (2016). Repression of Pumilio protein expression by Rbfox1 promotes germ cell differentiation. Dev. Cell *36*, 562–571.

Chekulaeva, M., Hentze, M.W., and Ephrussi, A. (2006). Bruno acts as a dual repressor of oskar translation, promoting mRNA oligomerization and formation of silencing particles. Cell *124*, 521–533.

Chen, D., and McKearin, D.M. (2003). A discrete transcriptional silencer in the bam gene determines asymmetric division of the Drosophila germline stem cell. Development *130*, 1159–1170.

Cho, P.F., Poulin, F., Cho-Park, Y.A., Cho-Park, I.B., Chicoine, J.D., Lasko, P., and Sonenberg, N. (2005). A new paradigm for translational control: inhibition via 5′-3′ mRNA tethering by Bicoid and the eIF4E cognate 4EHP. Cell *121*, 411–423.

Cho, P.F., Gamberi, C., Cho-Park, Y.A., Cho-Park, I.B., Lasko, P., and Sonenberg, N. (2006). Cap-dependent translational inhibition establishes two opposing morphogen gradients in Drosophila embryos. Curr. Biol. *16*, 2035–2041.

Cinalli, R.M., Rangan, P., and Lehmann, R. (2008). Germ cells are forever. Cell *132*, 559–562.

Ciosk, R., DePalma, M., and Priess, J.R. (2006). Translational regulators maintain totipotency in the Caenorhabditis elegans germline. Science *311*, 851–853.

Draper, B.W., Mello, C.C., Bowerman, B., Hardin, J., and Priess, J.R. (1996). MEX-3 is a KH domain protein that regulates blastomere identity in early C. elegans embryos. Cell *87*, 205–216.

Eichhorn, S.W., Subtelny, A.O., Kronja, I., Kwasnieski, J.C., Orr-Weaver, T.L., and Bartel, D.P. (2016). mRNA poly(A)-tail changes specified by deadenylation broadly reshape translation in Drosophila oocytes and early embryos. eLife *5*, 714.

Eulalio, A., Rehwinkel, J., Stricker, M., Huntzinger, E., Yang, S.-F., Doerks, T., Dorner, S., Bork, P., Boutros, M., and Izaurralde, E. (2007). Target-specific requirements for enhancers of decapping in miRNA-mediated gene silencing. Genes Dev. *21*, 2558–2570.

Fan, S.-J., Marchand, V., and Ephrussi, A. (2011). Drosophila Ge-1 promotes P body formation and oskar mRNA localization. PLoS ONE *6*, e20612.

Flora, P., Schowalter, S., Wong-Deyrup, S., DeGennaro, M., Nasrallah, M.A., and Rangan, P. (2018). Transient transcriptional silencing alters the cell cycle to promote germline stem cell differentiation in Drosophila. Dev. Biol. *434*, 84–95.

Forbes, A., and Lehmann, R. (1998). Nanos and Pumilio have critical roles in the development and function of Drosophila germline stem cells. Development *125*, 679–690.

Fu, Z., Geng, C., Wang, H., Yang, Z., Weng, C., Li, H., Deng, L., Liu, L., Liu, N., Ni, J., and Xie, T. (2015). Twin promotes the maintenance and differentiation of germline stem cell lineage through modulation of multiple pathways. Cell Rep. *13*, 1366–1379.

Garneau, N.L., Wilusz, J., and Wilusz, C.J. (2007). The highways and byways of mRNA decay. Nat. Rev. Mol. Cell Biol. *8*, 113–126.

Gavis, E.R., and Lehmann, R. (1992). Localization of nanos RNA controls embryonic polarity. Cell *71*, 301–313.

Gavis, E.R., and Lehmann, R. (1994). Translational regulation of nanos by RNA localization. Nature *369*, 315–318.

Gilboa, L., and Lehmann, R. (2004). How different is Venus from Mars? The genetics of germ-line stem cells in Drosophila females and males. Development *131*, 4895–4905.

Hanyu-Nakamura, K., Sonobe-Nojima, H., Tanigawa, A., Lasko, P., and Nakamura, A. (2008). Drosophila Pgc protein inhibits P-TEFb recruitment to chromatin in primordial germ cells. Nature *451*, 730–733.

Harris, R.E., Pargett, M., Sutcliffe, C., Umulis, D., and Ashe, H.L. (2011). Brat promotes stem cell differentiation via control of a bistable switch that restricts BMP signaling. Dev. Cell *20*, 72–83.

Harrison, M.M., Li, X.-Y., Kaplan, T., Botchan, M.R., and Eisen, M.B. (2011). Zelda binding in the early Drosophila melanogaster embryo marks regions subsequently activated at the maternal-to-zygotic transition. PLoS Genet. *7*, e1002266.

He, J., Stewart, K., Kinnell, H.L., Anderson, R.A., and Childs, A.J. (2013). A developmental stage-specific switch from DAZL to BOLL occurs during fetal oogenesis in humans, but not mice. PLoS ONE *8*, e73996.

Jenkins, H.T., Baker-Wilding, R., and Edwards, T.A. (2009). Structure and RNA binding of the mouse Pumilio-2 Puf domain. J. Struct. Biol. *167*, 271–276.

Johnstone, O., and Lasko, P. (2001). Translational regulation and RNA localization in Drosophila oocytes and embryos. Annu. Rev. Genet. *35*, 365–406.

Joly, W., Chartier, A., Rojas-Rios, P., Busseau, I., and Simonelig, M. (2013). The CCR4 deadenylase acts with Nanos and Pumilio in the fine-tuning of Mei-P26 expression to promote germline stem cell self-renewal. Stem Cell Reports *1*, 411–424.

Kadyrova, L.Y., Habara, Y., Lee, T.H., and Wharton, R.P. (2007). Translational control of maternal Cyclin B mRNA by Nanos in the Drosophila germline. Development *134*, 1519–1527.

Kim, D., Langmead, B., and Salzberg, S.L. (2015a). HISAT: a fast spliced aligner with low memory requirements. Nat. Methods *12*, 357–360.

Kim, G., Pai, C.I., Sato, K., Person, M.D., Nakamura, A., and Macdonald, P.M. (2015b). Region-specific activation of oskar mRNA translation by inhibition of Bruno-mediated repression. PLoS Genet *11*, e1004992.

Kim-Ha, J., Kerr, K., and Macdonald, P.M. (1995). Translational regulation of oskar mRNA by bruno, an ovarian RNA-binding protein, is essential. Cell *81*, 403–412.

Kress, C., Gautier-Courteille, C., Osborne, H.B., Babinet, C., and Paillard, L. (2007). Inactivation of CUG-BP1/CELF1 causes growth, viability, and spermatogenesis defects in mice. Mol. Cell. Biol. *27*, 1146–1157.

Kronja, I., Yuan, B., Eichhorn, S.W., Dzeyk, K., Krijgsveld, J., Bartel, D.P., and Orr-Weaver, T.L. (2014). Widespread changes in the posttranscriptional landscape at the Drosophila oocyte-to-embryo transition. Cell Rep. *7*, 1495–1508.

Lasko, P. (2012). mRNA localization and translational control in Drosophila oogenesis. Cold Spring Harb. Perspect. Biol. *4*, a012294.

Laver, J.D., Marsolais, A.J., Smibert, C.A., and Lipshitz, H.D. (2015). Regulation and function of maternal gene products during the maternal-to-zygotic transition in Drosophila. Curr. Top. Dev. Biol. *113*, 43–84.

Lee, M.T., Bonneau, A.R., and Giraldez, A.J. (2014). Zygotic genome activation during the maternal-to-zygotic transition. Annu. Rev. Cell Dev. Biol. *30*, 581–613.

Li, Y., Minor, N.T., Park, J.K., McKearin, D.M., and Maines, J.Z. (2009). Bam and Bgcn antagonize Nanos-dependent germ-line stem cell maintenance. Proc. Natl. Acad. Sci. USA *106*, 9304–9309.

Liang, H.-L., Nien, C.-Y., Liu, H.-Y., Metzstein, M.M., Kirov, N., and Rushlow, C. (2008). The zinc-finger protein Zelda is a key activator of the early zygotic genome in Drosophila. Nature *456*, 400–403.

Liao, Y., Smyth, G.K., and Shi, W. (2014). featureCounts: an efficient general purpose program for assigning sequence reads to genomic features. Bioinformatics *30*, 923–930.

Lin, H., and Spradling, A.C. (1993). Germline stem cell division and egg chamber development in transplanted Drosophila germaria. Dev. Biol. *159*, 140–152.

Mak, W., Fang, C., Holden, T., Dratver, M.B., and Lin, H. (2016). An important role of Pumilio 1 in regulating the development of the mammalian female germline. Biol. Reprod. *94*, 134.

Margolis, J., and Spradling, A. (1995). Identification and behavior of epithelial stem cells in the Drosophila ovary. Development *121*, 3797–3807.

Martinho, R.G., Kunwar, P.S., Casanova, J., and Lehmann, R. (2004). A noncoding RNA is required for the repression of RNApolII-dependent transcription in primordial germ cells. Curr. Biol. *14*, 159–165.

McKearin, D., and Ohlstein, B. (1995). A role for the Drosophila bag-of-marbles protein in the differentiation of cystoblasts from germline stem cells. Development *121*, 2937–2947.

McKearin, D.M., and Spradling, A.C. (1990). bag-of-marbles: a Drosophila gene required to initiate both male and female gametogenesis. Genes Dev. *4* (*12B*), 2242–2251.

Merritt, C., Rasoloson, D., Ko, D., and Seydoux, G. (2008). 3′ UTRs are the primary regulators of gene expression in the C. elegans germline. Curr. Biol. *18*, 1476–1482.

Meyer, S., Temme, C., and Wahle, E. (2004). Messenger RNA turnover in eukaryotes: pathways and enzymes. Crit. Rev. Biochem. Mol. Biol. *39*, 197–216.

Mootz, D., Ho, D.M., and Hunter, C.P. (2004). The STAR/Maxi-KH domain protein GLD-1 mediates a developmental switch in the translational control of C. elegans PAL-1. Development *131*, 3263–3272.

Morris, J.Z., Hong, A., Lilly, M.A., and Lehmann, R. (2005). twin, a CCR4 homolog, regulates cyclin poly(A) tail length to permit Drosophila oogenesis. Development *132*, 1165–1174.

Muraro, N.I., Weston, A.J., Gerber, A.P., Luschnig, S., Moffat, K.G., and Baines, R.A. (2008). Pumilio binds para mRNA and requires Nanos and Brat to regulate sodium current in Drosophila motoneurons. J. Neurosci. *28*, 2099–2109.

Murata, Y., and Wharton, R.P. (1995). Binding of pumilio to maternal hunchback mRNA is required for posterior patterning in Drosophila embryos. Cell *80*, 747–756.

Nakamura, A., Amikura, R., Mukai, M., Kobayashi, S., and Lasko, P.F. (1996). Requirement for a noncoding RNA in Drosophila polar granules for germ cell establishment. Science *274*, 2075–2079.

Nakamura, A., Amikura, R., Hanyu, K., and Kobayashi, S. (2001). Me31B silences translation of oocyte-localizing RNAs through the formation of cytoplasmic RNP complex during Drosophila oogenesis. Development *128*, 3233–3242.

Nakamura, A., Sato, K., and Hanyu-Nakamura, K. (2004). Drosophila cup is an eIF4E binding protein that associates with Bruno and regulates oskar mRNA translation in oogenesis. Dev. Cell *6*, 69–78.

Nien, C.-Y., Liang, H.-L., Butcher, S., Sun, Y., Fu, S., Gocha, T., Kirov, N., Manak, J.R., and Rushlow, C. (2011). Temporal coordination of gene networks by Zelda in the early Drosophila embryo. PLoS Genet. *7*, e1002339.

Olesnicky, E.C., Bhogal, B., and Gavis, E.R. (2012). Combinatorial use of translational co-factors for cell type-specific regulation during neuronal morphogenesis in Drosophila. Dev. Biol. *365*, 208–218.

Ozgur, S., Basquin, J., Kamenska, A., Filipowicz, W., Standart, N., and Conti, E. (2015). Structure of a human 4E-T/DDX6/CNOT1 complex reveals the different interplay of DDX6-binding proteins with the CCR4-NOT complex. Cell Rep. *13*, 703–711.

Rangan, P., DeGennaro, M., and Lehmann, R. (2008). Regulating gene expression in the Drosophila germ line. Cold Spring Harb. Symp. Quant. Biol. *73*, 1–8.

Rangan, P., DeGennaro, M., Jaime-Bustamante, K., Coux, R.-X., Martinho, R.G., and Lehmann, R. (2009). Temporal and spatial control of germ-plasm RNAs. Curr. Biol. *19*, 72–77.

Richter, J.D., and Lasko, P. (2011). Translational control in oocyte development. Cold Spring Harb. Perspect. Biol. *3*, a002758.

Rosario, R., Childs, A.J., and Anderson, R.A. (2017). RNA-binding proteins in human oogenesis: Balancing differentiation and self-renewal in the female fetal germline. Stem Cell Res. (Amst.) *21*, 193–201.

Sachs, A., and Wahle, E. (1993). Poly(A) tail metabolism and function in eucaryotes. J. Biol. Chem. *268*, 22955–22958.

Sallés, F.J., and Strickland, S. (1999). Analysis of poly(A) tail lengths by PCR: the PAT assay. Methods Mol. Biol. *118*, 441–448.

Schüpbach, T., and Wieschaus, E. (1991). Female sterile mutations on the second chromosome of Drosophila melanogaster. II. Mutations blocking oogenesis or altering egg morphology. Genetics *129*, 1119–1136.

Serano, T.L., Cheung, H.-K., Frank, L.H., and Cohen, R.S. (1994). P element transformation vectors for studying Drosophila melanogaster oogenesis and early embryogenesis. Gene *138*, 181–186.

Seydoux, G., and Braun, R.E. (2006). Pathway to totipotency: lessons from germ cells. Cell *127*, 891–904.

Slaidina, M., and Lehmann, R. (2014). Translational control in germline stem cell development. J. Cell Biol. *207*, 13–21.

Snee, M., Benz, D., Jen, J., and Macdonald, P.M. (2014). Two distinct domains of Bruno bind specifically to the oskarmRNA. RNA Biol. *5*, 49–57.

Sonoda, J., and Wharton, R.P. (1999). Recruitment of Nanos to hunchback mRNA by Pumilio. Genes Dev. *13*, 2704–2712.

Sonoda, J., and Wharton, R.P. (2001). Drosophila Brain Tumor is a translational repressor. Genes Dev. *15*, 762–773.

Spassov, D.S., and Jurecic, R. (2003). Mouse Pum1 and Pum2 genes, members of the Pumilio family of RNA-binding proteins, show differential expression in fetal and adult hematopoietic stem cells and progenitors. Blood Cells Mol. Dis. *30*, 55–69.

Spradling, A.C. (1993). Developmental Genetics of Oogenesis, *Volume 1* (Cold Spring Harbor Laboratory Press).

Spradling, A.C., de Cuevas, M., Drummond-Barbosa, D., Keyes, L., Lilly, M., Pepling, M., and Xie, T. (1997). The Drosophila germarium: stem cells, germ line cysts, and oocytes. Cold Spring Harb. Symp. Quant. Biol. *62*, 25–34.

Sugimura, I., and Lilly, M.A. (2006). Bruno inhibits the expression of mitotic cyclins during the prophase I meiotic arrest of Drosophila oocytes. Dev. Cell *10*, 127–135.

Tadros, W., and Lipshitz, H.D. (2009). The maternal-to-zygotic transition: a play in two acts. Development *136*, 3033–3042.

Temme, C., Zhang, L., Kremmer, E., Ihling, C., Chartier, A., Sinz, A., Simonelig, M., and Wahle, E. (2010). Subunits of the Drosophila CCR4-NOT complex and their roles in mRNA deadenylation. RNA *16*, 1356–1370.

Temme, C., Simonelig, M., and Wahle, E. (2014). Deadenylation of mRNA by the CCR4-NOT complex in Drosophila: molecular and developmental aspects. Front. Genet. *5*, 143.

Timinszky, G., Bortfeld, M., and Ladurner, A.G. (2008). Repression of RNA polymerase II transcription by a Drosophila oligopeptide. PLoS ONE *3*, e2506.

Trcek, T., Lionnet, T., Shroff, H., and Lehmann, R. (2017). mRNA quantification using single-molecule FISH in Drosophila embryos. Nat. Protoc. *12*, 1326–1348.

Vlasova, I.A., Tahoe, N.M., Fan, D., Larsson, O., Rattenbacher, B., Sternjohn, J.R., Vasdewani, J., Karypis, G., Reilly, C.S., Bitterman, P.B., and Bohjanen, P.R. (2008). Conserved GU-rich elements mediate mRNA decay by binding to CUG-binding protein 1. Mol. Cell *29*, 263–270.

Wagnon, J.L., Mahaffey, C.L., Sun, W., Yang, Y., Chao, H.T., and Frankel, W.N. (2011). Etiology of a genetically complex seizure disorder in Celf4 mutant mice. Genes Brain Behav. *10*, 765–777.

Wang, Z., and Lin, H. (2005). The division of Drosophila germline stem cells and their precursors requires a specific cyclin. Curr. Biol. *15*, 328–333.

Wang, X., Zamore, P.D., and Hall, T.M. (2001). Crystal structure of a Pumilio homology domain. Mol. Cell *7*, 855–865.

Webster, P.J., Liang, L., Berg, C.A., Lasko, P., and Macdonald, P.M. (1997). Translational repressor bruno plays multiple roles in development and is widely conserved. Genes Dev. *11*, 2510–2521.

Weidmann, C.A., Qiu, C., Arvola, R.M., Lou, T.-F., Killingsworth, J., Campbell, Z.T., Tanaka Hall, T.M., and Goldstrohm, A.C. (2016). Drosophila Nanos acts as a molecular clamp that modulates the RNA-binding and repression activities of Pumilio. eLife *5*, 1948.

Wharton, R.P., and Struhl, G. (1991). RNA regulatory elements mediate control of Drosophila body pattern by the posterior morphogen nanos. Cell *67*, 955–967.

Wharton, R.P., Sonoda, J., Lee, T., Patterson, M., and Murata, Y. (1998). The Pumilio RNA-binding domain is also a translational regulator. Mol. Cell *1*, 863–872.

Wilkie, G.S., Dickson, K.S., and Gray, N.K. (2003). Regulation of mRNA translation by 5′- and 3′-UTR-binding factors. Trends Biochem. Sci. *28*, 182–188.

Xie, T., and Spradling, A.C. (1998). decapentaplegic is essential for the maintenance and division of germline stem cells in the Drosophila ovary. Cell *94*, 251–260.

Xin, T., Xuan, T., Tan, J., Li, M., Zhao, G., and Li, M. (2013). The Drosophila putative histone acetyltransferase Enok maintains female germline stem cells through regulating Bruno and the niche. Dev. Biol. *384*, 1–12.

Zhang, K., and Smith, G.W. (2015). Maternal control of early embryogenesis in mammals. Reprod. Fertil. Dev. *27*, 880–896.

Zhang, M., Chen, D., Xia, J., Han, W., Cui, X., Neuenkirchen, N., Hermes, G., Sestan, N., and Lin, H. (2017). Post-transcriptional regulation of mouse neurogenesis by Pumilio proteins. Genes Dev. *31*, 1354–1369.

STAR★METHODS

KEY RESOURCES TABLE

REAGENT or RESOURCE	SOURCE	IDENTIFIER
Antibodies		
Rabbit polyclonal anti-GFP	abCam	Cat# ab6556
Chicken polyclonal anti-GFP	abCam	Cat# ab13970
Rabbit polyclonal anti-pMad	abCam	Cat# ab52903
Mouse monoclonal anti-1B1	Developmental studies Hybridoma Bank	Antibody Registry ID:528070
Rat monoclonal anti-HA high affinity	Roche Diagnostics	REF:11867423001
Rabbit polyclonal anti-Vasa	Rangan Lab	N/A
Chicken polyclonal anti-Vasa	Rangan Lab	N/A
Rabbit polyclonal anti-Pumilio	Gift from Lehmann Lab	N/A
Rabbit polyclonal anti-Bruno	Gift from Lehmann Lab	N/A
Rabbit polyclonal anti-Nanos	Gift from Buszczak Lab	N/A
Rabbit polyclonal anti-Bruno	Gift from Lily Lab (Sugimura and Lilly, 2006)	N/A
Anti-rabbit Alexa 488	Jackson ImmunoResearch Labs	Code:711-546-152
Anti-chicken Alexa 488	Jackson ImmunoResearch Labs	Code:703-546-155
Anti-rabbit Alexa Cy3	Jackson ImmunoResearch Labs	Code:711-166-152
Anti-mouse Alexa Cy3	Jackson ImmunoResearch Labs	Code:715-546-150
Anti-chicken Alexa 647	Jackson ImmunoResearch Labs	Code:703-606-155
Anti-mouse Alexa 647	Jackson ImmunoResearch Labs	Code:715-606-150
Anti-Rat HRP	Jackson ImmunoResearch Labs	Code:112-035-003
Anti-Rabbit HRP	Jackson ImmunoResearch Labs	Code:111-035-144
ChromePure Rabbit IgG	Jackson ImmunoResearch Labs	Code: 011-000-003
Bacterial and Virus Strains		
BL21(DE3) competent *E.coli*	New England Biolabs Inc.	Cat# C25271
KRX *E.coli* competent cells	Promega	Cat# L3002
DH5α competent cells	Invitrogen	Cat# 18265017
Chemicals, Peptides, and Recombinant Proteins		
Formaldehyde (Methanol Free),10% Ultrapure	Polysciences Inc.	Cat# 04018-1
Donkey Serum	Sigma-Aldrich	SKU: D9663
Vectashield Antifade Mounting Medium with DAPI	Vector Laboratories	Cat# H-1200
T4 Polynucleotide Kinase	New England Biolabs Inc.	Cat# M0201S
Restriction Endonuclease XhoI	New England Biolabs Inc.	Cat# R0146S
Restriction Endonuclease KpnI	New England Biolabs Inc.	Cat# R0142S
Restriction Endonuclease AgeI	New England Biolabs Inc.	Cat# R0552S
Restriction Endonuclease SpeI	New England Biolabs Inc.	Cat# R0133S
Restriction Endonuclease NotI	New England Biolabs Inc.	Cat# R0189S
Phusion High-Fidelity DNA Polymerase	New England Biolabs Inc.	Cat# M0530S
HaloLink Resin	Promega	Cat# G1912
L-Rhamnose monohydrate	Sigma-Aldrich	SKU: R3875
IPTG	Invitrogen	Cat# 15529019
AcTEV Protease	Invitrogen	Cat# 12575015
LightShift Poly (dI-dC)	ThermoFisher Scientific	Cat# 20148E
Yeast tRNA	ThermoFisher Scientific	Cat# AM7119
Salmon Sperm DNA	ThermoFisher Scientific	Cat# 15632011

(*Continued on next page*)

Continued

REAGENT or RESOURCE	SOURCE	IDENTIFIER
Nonidet P-40 (NP-40) substitute	IBI Scientific	Cas# 9016-45-9
Tween-20 detergent	VWR	Cat# 97062-332
Triton X-100 detergent	VWR	Cat# 97062-208
Igepal CA-630 detergent	Sigma-Aldrich	SKU: I8896
DNase I	Roche	Cat# 04 716 728 001
Aprotinin	Sigma-Aldrich	SKU: 10236624001
PMSF	Sigma-Aldrich	SKU: 10837091001
Leupeptin protease inhibitor	ThermoFisher Scientific	Cat# 78435
Pepstatin A protease inhibitor	ThermoFisher Scientific	Cat# 78436
TRIzol	Invitrogen	Cat# 15596026
Dynabeads Protein A	Invitrogen	Cat# 10002D
cOmplete, EDTA-free Protease Inhibitor Cocktail Pill	Sigma-Aldrich	SKU: 11873580001
Bradford reagent	Bio-Rad	Cat. #500-0205
4X Laemmli Sample Buffer	Bio-Rad	Cat. #161-0747
Ultrapure Sucrose	Amresco	Code: 0335-1KG
Bruno expression plasmid pETM-82	EMBL (Chekulaeva et al., 2006)	N/A
Pumilio expression plasmid pFN18K	Goldstrohm Lab (Weidmann et al., 2016)	N/A
Critical Commercial Assays		
G-25 Sephadix Columns	Roche	Cat# 11273990001
PD-10 column	GE Health care Life Sciences	Cat# 17-0851-01
His GraviTrap	GE Health care Life Sciences	Cat# 11-0033-99
TURBO DNA-free Kit	Life Technologies	Cat# AM1907
Super Script III	Life Technologies	Cat# 1808051
SYBR Green Master Mix	Applied Biosystems	Cat# 4367659
NEXTflex Rapid Illumina DNA-Seq Library Prep Kit	Bioo Scientific	Cat# NOVA-5138-11
Mini-PROTEAN TGX 4-20% gradient SDS-PAGE gels	Bio-Rad	Cat# 456-1094
Western ECL Substrate	Bio-Rad	Cat# 1705060
Deposited Data		
RNA-seq Data	This paper	GEO: GSE119458
Polysome-seq Data	This paper	GEO: GSE119458
Experimental Models: Organisms/Strains		
D. melanogaster: w*; pgc^{Δ}	(Martinho et al., 2004; Flora et al., 2018)	N/A
D. melanogaster: w*; Df(2R)Liprin-γ^{H1}, P{neoFRT}42D Liprin-γ^{H1}/CyO	Bloomington Drosophila Stock Center (Astigarraga et al., 2010)	BDSC:63813; FlyBase: FBst0063813
D. melanogaster: w*; P{UAS-tkv.CA}3	Bloomington Drosophila Stock Center	BDSC:36537; FlyBase: FBst0036537
D. melanogaster: pum^{FC8} mutant	(Forbes and Lehmann, 1998)	N/A
D. melanogaster: pum^{ET1} mutant	(Forbes and Lehmann, 1998)	N/A
D. melanogaster: RNAi for *pum:* y^1 v^1; P{TRiP.JF02267}attP2	Bloomington Drosophila Stock Center	BDSC:26725; FlyBase: FBst0026725
D. melanogaster: RNAi for *pum:* y^1 sc* v^1; P{TRiP.HMS01685}attP40	Bloomington Drosophila Stock Center	BDSC:38241; FlyBase: FBst0038241
D. melanogaster: st^1 pum^{680}/TM3, Sb^1 Ser^1	Bloomington Drosophila Stock Center (Wharton et al., 1998)	BDSC:3260; FlyBase: FBst0003260
D. melanogaster: nos^{RC} mutant	(Arrizabalaga and Lehmann, 1999)	N/A
D. melanogaster: nos^{BN} mutant	(Arrizabalaga and Lehmann, 1999)	N/A

(*Continued on next page*)

Continued

REAGENT or RESOURCE	SOURCE	IDENTIFIER
D. melanogaster: RNAi for *nos:* y^1 sc* v^1; P{TRiP.HMS00930}attP2	Bloomington Drosophila Stock Center	BDSC:33973; FlyBase: FBst0033973
D. melanogaster: RNAi for *nos:* y^1 sc* v^1; P{TRiP.GLC01867}attP40	Bloomington Drosophila Stock Center	BDSC:57700 FlyBase: FBst0057700
D. melanogaster: *twin*^{RY3} mutant	(Morris et al., 2005)	N/A
D. melanogaster: *twin*^{RY5} mutant	(Morris et al., 2005)	N/A
D. melanogaster: RNAi for *twin:* y^1 sc* v^1; P{TRiP.HMS00493}attP2	Bloomington Drosophila Stock Center	BDSC:32490; FlyBase: FBst0032490
D. melanogaster: RNAi for *not:* y^1 sc* v^1; P{TRiP.HMS00526}attP2	Bloomington Drosophila Stock Center	BDSC:32836; FlyBase: FBst0032836
D. melanogaster: RNAi for *pop2:* y^1 sc* v^1; P{TRiP.HM05235}attP2	Bloomington Drosophila Stock Center	BDSC:30492; FlyBase: FBst0030492
D. melanogaster: RNAi for *me31B:* y^1 v^1; P{TRiP.HM05052}attP2	Bloomington Drosophila Stock Center	BDSC:28566; FlyBase: FBst0028566
D. melanogaster: RNAi for *dGe1:* y^1 sc* v^1; P{TRiP.HMS00340}attP2	Bloomington Drosophila Stock Center	BDSC:32349; FlyBase: FBst0032349
D. melanogaster: RNAi for *Brat:* y^1 sc* v^1; P{TRiP.HMS01121}attP2	Bloomington Drosophila Stock Center	BDSC:34646; FlyBase: FBst0034646
D. melanogaster: RNAi for *d4eHP:* y^1 sc* v^1; P{TRiP.GL01035}attP2	Bloomington Drosophila Stock Center	BDSC:36876; FlyBase: FBst0036876
D. melanogaster: *aret*^{QB} (*bruno*) mutant	Schupbach Lab (Schüpbach and Wieschaus, 1991)	N/A
D. melanogaster: *aret*^{PA} (*bruno*) mutant	Schupbach Lab (Schüpbach and Wieschaus, 1991)	N/A
D. melanogaster: RNAi for *bru*RNAi: y^1 v^1; P{TRiP.HMS01899}attP40	Bloomington Drosophila Stock Center	BDSC:38983; FlyBase: FBst0038983
D. melanogaster: RNAi for *cup*RNAi: y^1 sc* v^1; P{TRiP.GL00327}attP2/TM3, Sb^1	Bloomington Drosophila Stock Center	BDSC:35406; FlyBase: FBst0035406
D. melanogaster: *nosGAL4::VP16*	Lehmann Lab (NYUMC)	N/A
D. melanogaster: *nosGAL4.NGT*	Lehmann Lab (NYUMC)	N/A
D. melanogaster: *pumGFP* transgene	Gift from Salz Lab (Case Western)	N/A
D. melanogaster: *me31BGFP*-TRAP transgene	Gift from Nakamura Lab (RIKEN)	N/A
D. melanogaster: *pgcGFP* transgene (P-P-P)	Rangan Lab (Flora et al., 2018)	N/A
D. melanogaster: *pgc* promoter-*pgc* 5′UTR-eGFP-*tubulin* 3′UTR transgene (P-P-T)	This paper	N/A
D. melanogaster: *pgc* promoter-*pgc* 5′UTR-eGFP-*K10* 3′UTR (P-P-K)	This paper	N/A
D. melanogaster: *pgc* promoter-*nos* 5′UTR-eGFP-*K10* 3′UTR (P-N-K)	This paper	N/A
D. melanogaster: *pgc* promoter-*nos* 5′UTR-eGFP-*pgc*3′UTR (P-N-P)	This paper	N/A
D. melanogaster: *pgc* promoter-*pgc* 5′UTR-eGFP-*tubulin* 3′UTR+(NBS+PRE/BRE) transgene (P-P-T: NBS+PRE/BRE)	This paper	N/A
D. melanogaster: *pgcGFP* transgene (P-P-P: ΔUGUAAAUU)	This paper	N/A
D. melanogaster: *pgcGFP* transgene (P-P-P: UUUUAAUU)	This paper	N/A
D. melanogaster: *pgcGFP* transgene (P-P-P: UCUCAAUU)	This paper	N/A

(*Continued on next page*)

Continued

REAGENT or RESOURCE	SOURCE	IDENTIFIER
D. melanogaster: *pgcGFP* transgene (P-P-P: ΔUGUA)	This paper	N/A
Oligonucleotides		
Primers used for generating transgenes see Table S5	This paper	N/A
Primers for site-directed mutagenesis see Table S5	This paper	N/A
Oligonucleotides for EMSA see Table S5	This paper	N/A
Primers for RT-PCR see Table S5	This paper	N/A
Primers for qRT-PCR see Table S5	This paper	N/A
Primers for PAT assay see Table S5	This paper	N/A
GFP RNA FISH probe labeled with CALFluor590	(Trcek et al., 2017)	N/A
pgc RNA FISH probe labeled with CALFluor590	(Trcek et al., 2017)	N/A
Recombinant DNA		
Plasmid: pCaSpeR2 P element transformation vector	Drosophila Genomics Resource Center	Stock Number: 1066
Software and Algorithms		
ImageJ	NIH	https://imagej.nih.gov/ij/
MEME Suite	(Bailey et al., 2009)	http://meme-suite.org/doc/overview.html
HISAT2	(Kim et al., 2015a)	https://ccb.jhu.edu/software/hisat2/index.shtml
featureCounts	(Liao et al., 2014)	http://bioinf.wehi.edu.au/featureCounts/
R package Biostrings	Bioconductor	https://bioconductor.org/packages/release/bioc/html/Biostrings.html
PANTHER Gene Analysis	Gene Ontology Reference Genome Project	http://www.pantherdb.org/

CONTACT FOR REAGENT AND RESOURCE SHARING

Further information and requests for resources and reagents should be directed to Lead Contact, Dr. Prashanth Rangan (prangan@albany.edu).

EXPERIMENTAL MODEL AND SUBJECT DETAILS

Fly strains

Drosophila was grown on corn flour and agar media with brewer's yeast. All strains were grown at 25°C, except RNAi crosses, which were grown at 29°C. *pgcGFP and pgc*$^{\Delta}$ used in this study have been previously reported (Martinho et al., 2004; Flora et al., 2018). *liprin-γ*^{H1} flies were a gift from the Triesman Lab (Astigarraga et al., 2010). *nos* mutants were generated by crossing the *nos*^{RC} and *nos*^{BN} alleles (Arrizabalaga and Lehmann 1999). *pum* mutants were created by crossing the *pum*^{FC8} and *pum*^{ET1} alleles (Forbes and Lehmann 1998). *twin* mutants were created by crossing the *twin*^{ry3} and *twin*^{ry5} (Morris et al., 2005). The *pum*^{680} allele is described in Wharton *et.al*.,1998. *aret* mutants were created by crossing the *aret*^{PA} and *aret*^{QB} (Schüpbach and Wieschaus 1991). ·*nosGAL4::VP16* and *nosGAL4.NGT* was gifted by the Lehmann lab. *w*^{1118}, *nos*RNAi, *pum*RNAi, *twin*RNAi, *brat*RNAi, *d4EHP*RNAi, *not1*RNAi, *pop2*RNAi, *Me31B*RNAi, d*Ge-1*RNAi, *bru*RNAi and *cup*RNAi lines were acquired from the Bloomington *Drosophila* Stock Center, Bloomington, IN. The transgenic flies in this paper were generated in the Rangan Lab. They are as follows: P-P-P/*pgcGFP* (*pgc* promoter-*pgc* 5′UTR-eGFP-*pgc* 3′UTR) (Flora et al., 2018), P-P-T (*pgc* promoter-*pgc* 5′UTR-eGFP-*α-tubulin84B* 3′UTR), P-P-K (*pgc* promoter-*pgc* 5′UTR-eGFP-*K10* 3′UTR), P-N-K (*pgc* promoter-*nos* 5′UTR-eGFP-*K10* 3′UTR), generate P-N-P (*pgc* promoter-*nos* 5′UTR-eGFP-*pgc*3′UTR), P-P-T:NBS+PRE/BRE (*pgc* promoter-*pgc* 5′UTR-eGFP-*α-tubulin84B* 3′UTR: NBS+PRE/BRE), P-P-P: ΔUGUAAAUU (*pgc* promoter-*pgc* 5′UTR-eGFP-*pgc* 3′UTR: ΔUGUAAAUU), P-P-P: ΔUGUA (*pgc* promoter-*pgc* 5′UTR-eGFP-*pgc* 3′UTR: ΔUGUA), P-P-P: UUUUAAUU (*pgc* promoter-*pgc* 5′UTR-eGFP-*pgc* 3′UTR: UUUUAAUU), P-P-P: UCUCAAUU (*pgc* promoter-*pgc* 5′UTR-eGFP-*pgc* 3′UTR: UCUCAAUU).

METHOD DETAILS

Generation of transgenic fly strains

The P-P-P/*pgcGFP* construct was generated by cloning eGFP coding sequence into a plasmid with the *pgc* 5′UTR and *pgc* 3′UTR as previously described (Flora et al., 2018).The P-P-T and P-P-K constructs were assembled by PCR amplifying a XhoI-KpnI fragment containing the α-*tubulin84B* (*tub*) 3′UTR or *K10* 3′UTR was then cloned into the XhoI-KpnI site of the P-P-P plasmid, respectively. In order to allow for interchanging of the 700 bp *pgc* promoter and *pgc* 5′UTR region of P-P-K, AgeI site was created between of those regions of P-P-K via GenScript by Fisher Scientific. The P-N-K construct was then generated by inserting the *nos* 5′UTR with AgeI and SpeI overhangs into the AgeI-SpeI site of the P-P-K plasmid. The *pgc* 3′UTR fragment was cloned downstream of eGFP at the XhoI-KpnI site of P-N-K to generate P-N-P. The P-P-P: ΔUGUAAAUU, P-P-P: ΔUGUA, P-P-P: UUUUAAUU and P-P-P: UCUCAAUU transgenes in (Figures 2 and S1) was created by site-directed mutagenesis using Phusion High-Fidelity DNA Polymerase. The primers used are listed separately. For the sufficiency experiment the P-P-T: NBS+PRE/BRE construct was generated by inserting the PRE/BRE sequence was added at the same location (after nucleotide 28 of *tub* 3′UTR) of that of *pgc* 3′UTR into α -tubulin 84B 3′UTR. These gene fragments were created from gBlock gene fragment service by Integrated DNA technology with XhoI and KpnI sites. The plasmids for injections were then constructed by cloning those gBlock fragments via restriction digest.

Immuno-fluorescence Staining

Female *Drosophila* ovaries were dissected in cold 1X PBS and fixed in 4% paraformaldehyde for 20 minutes at room temperature (RT). The tissue was permeabilized in 1mL of PBST (1X PBS, 0.2% Tween and 1% Triton-X) for 1 hour at RT. After permeabilization the tissues were blocked in 1mL of BBT (0.5% BSA in PBST) for 2 hours at RT. Then 0.5mL of primary antibody was added and tissues were placed on a nutator at 4°C overnight. The following steps were then carried out at RT. After incubation, ovaries were washed three times in 1mL of BBT for 10, 15, 30 minutes. An additional wash for 30 minutes was carried on by adding 2% Donkey serum to 1mL of BBT. After the last wash secondary antibody in 0.5ml of BBT with 4% Donkey serum was added and incubated for 2 hours protected from light. After the incubation, ovaries were washed in 1mL of PBST for five times. After the washed one-drop of Vectashield was added and then the tissue was mounted on a glass slide and a coverslip was placed on the slide (Flora et al., 2018). The antibodies used and dilution are listed as follows: Rabbit anti-Vasa (1:4000 dilution), chicken anti-Vasa (1:500 dilution), mouse anti-1B1 (1:20), rabbit anti-GFP (1:2000), rabbit anti-pSmad3 (1:150), rabbit anti-Nanos (1:500), rabbit anti-Bruno (Lehmann Lab) (1:500), rabbit anti-Pumilio (1:150), Alexa 488, Cy3 and Cy5 conjugated secondary antibodies were used at a concentration of 1:500.

Fluorescent *in situ* hybridization (FISH)

FISH of the ovaries was carried out probes against pgc and GFP, which were a gift from the Lehmann lab (Trcek et al., 2017). The ovaries were dissected in 1XPBS, fixed in 3% methanol-free paraformaldehyde in PBS for 20 minutes and washed 3 times with PBST. Next, they were treated with 3 ug/ml Proteinase K in PBS and placed on a nutator for 13 minutes at RT, and then placed on ice for 30 minutes. The tissue was then blocked in 2 mg/ml glycine in PBST twice for 10 minutes each and rinsed twice with PBST for 2 minutes. The ovaries were post-fixed for 20 minutes in 3%. The tissue was then washed with PBST 5 times for 2 minutes and washed with pre-warmed fresh pre-hybridization mix (10% deionized formamide in 2X SSC) for 10 minutes. 60 μL per sample of hybridization mix (10% deionized formamide, 0.5 μL of yeast t-RNA, 0.5 μL of salmon sperm DNA, 1 μM of probe, 10% Dextran sulfate, 2 mg/ml BSA, 2X SSC and 1 μL of RNase Out) was added and the sample was incubated overnight at 37°C for at least 12 hours and no more than 16 hours. After incubation, 1 mL of pre-warmed pre-hybridization solution was added to the tissues. After 10 minutes, the pre-hybridization solution was removed, and the ovaries were washed 5 times with 1XPBS for 15 minutes each. After the last wash, PBS was aspirated out and a drop of Vectashield (Vector Labs, Inc.) was added to the tissue before preparing the slide.

Imaging

All images were taken on a Carl Zeiss 710 Meta confocal microscope using 20X or 40X oil immersion objectives. Scale bars were added using Zen Blue image processing software.

Western Blot

Twenty wild-type ovaries or 40 mutant ovaries were dissected in 1XPBS. Tissue was homogenized in 30 μL of RIPA buffer and centrifuged at 13,000 rpm for 15 minutes at 4°C. 1 μL of the protein extract was used to carry out a Bradford assay. 25 μg of protein sample was denatured with 4X Laemmli Sample Buffer and β-marcepthanol at 95°C for 5 minutes. The samples were loaded in a Mini-PROTEAN TGX 4%–20% gradient SDS-PAGE gels and run at 110V for 1 hour. The proteins were then transferred to a 0.20 μm nitrocellulose membrane at 100V for 1 hour at 4°C. After transfer, the membrane was blocked in 5% milk in PBST for 2 hours at RT and 1° antibody prepared in 5% milk in PBST was added to the membrane and incubated at 4°C O/N. The membrane was rinsed in 0.5% milk in PBST 4-5 times before adding 2° antibody prepared in 5% milk in PBST. After 2 hours the membrane was rinsed

in PBST 4-5 times. Chemiluminescence ECL kit was used to develop the membrane. The membrane was stripped prior to re-probing for loading control. Antibodies used for Western Blots are listed below:

Primary antibody rat anti-HA was used at 1:3000 dilution. Anti-rat HRP (1:10,000) was used at 1:10,000 dilution. Rabbit anti-Vasa (1:6000) was used as a loading control. Anti-rabbit HRP was used at 1:10,000 dilution.

For Western Blot analysis *pgcHA* levels were normalized to Vasa levels of each genotype. Then the fold change was calculated for each genotype by subtracting fold change of wild-type control from all experimental samples.

For RIP western blots, rabbit anti-Pum, rabbit anti-Bru and rabbit anti-GFP was used at a 1:4000, 1:6000 and 1:5000 dilution respectively. Anti-rabbit HRP was used at 1:10,000 dilution.

RNA Extraction

Wild-type ovaries were dissected in 1XPBS. After dissection, 100 μL of Trizol reagent was added to the tissue and homogenized. Additional, 900 μL of Trizol was added, mixed and incubated at RT for 3 minutes. After incubation, 200 μL of Chloroform was added to each sample and mixed vigorously and incubated at RT for 5 minutes before centrifugation at 13,000 rpm for 20 minutes at 4°C. 2 volumes of 100% ethanol, 10% volume 3 M sodium acetate and 0.5 ul of glycol blue was added to aqueous layer and incubated at −20°C for 1 hour. The samples were centrifuged at 13,000 rpm for 20 minutes at 4°C. The pellet was washed with 75% ethanol, air-dried and re-suspended in RNase free H_2O. 10 μg of nucleic acid was then taken and subjected to a DNase treatment using the TURBO DNA-free Kit.

Real Time-PCR (RT-PCR) and quantitative Real Time-PCR (qRT-PCR)

500ng of DNase treated RNA was reverse transcribed using Super Script III. For RT-PCR experiments, 1.5 μL of cDNA was amplified using 0.5 μL of 10 μM of each reverse and forward primers, 0.5 μL of 10 μM (d)NTP and 0.125 μL Taq Polymerase and 2.5 μL 10XTaq Polymerase Buffer. The thermal cycling conditions for PCR was 95°C for 30 s, 32 cycles of 95°C for 30 s, 3°below the T_m of the lowest T_m primer for 30 s, 68°C for 1 minute, and 1 cycle of 68°C for 4 minutes. After PCR, 2.8 μL of Orange-G dye was added to each sample and 10 μL of PCR product was ran on a 1% agarose gel stained with ethidium bromide to visualize bands.

For qRT-PCR experiments, 0.5 μL of cDNA was amplified using 5 μL of SYBR green Master Mix, 0.3 μL of 10 μM of each reverse and forward primers. The thermal cycling conditions were as follows: 50°C for 2 min, 95°C for 10 min, 40 cycles at 95°C for 15 s, and 60°C for 60 s. The experiments were carried out in technical triplicate and three biological replicates for each data point.

Pumilio Protein Purification

Pumilio expression plasmid pFN18K Pum RNA-binding domain (aa 1091-1426) was gifted to us by the Goldstrohm lab. Pumilio was purified following the protocol adapted from Weidmann et.al, 2016. The vector was transformed into KRX cells. A single colony from the plate was picked and inoculated in 100 mL of LB containing 25 μg/mL of kanamycin and incubated in a shaker at 37°C overnight. 20 mL of this starter culture was inoculated in 1L of 2xYT (16 g Bacto Tryptone, 10 g Bacto Yeast Extract, 5g NaCl, pH 7.0 adhusted with 5N NaOH) media containing 2mM $MgSO_4$ and 25 μg/mL of kanamycin and incubated in a shaker at 37°C till OD_{600} was between 0.7 and 0.9. Protein was induced for 3 hours in a shaker at 37°C by adding 5 mL of 20% w/v L-rhamnose (0.1% final). The cells were split into 500 mL aliquots and pelleted. Pumilio was purified from one pellet of 500 mL culture. Pellet was resuspended in 30 mL of filtered Bugwash (50mM Tris-HCl, pH 8.0, 10% w/v Sucrose) and centrifuged again. Supernatant was discarded. The pellet was resuspended in 25 mL of filtered Binding buffer (50mM Tris pH 8.0, 2mM $MgCl_2$, 150 mM NaCl) that contained freshly added 1mM DTT, 0.05% v/v NP-40 and 1x Protease inhibitor Cocktail (50X: 50 mM PMSF, 500 μg/ml aprotinin, 500 μg/ml pepstatin, 500 μg/ml leupeptin, dissolve in 10% v/v ethanol). After pellet was resuspended 1.25 mL of 10mg/ml lysozyme was added, mixed by inversion and incubated at 4°C for 30 minutes. Then 140 μL of 1M $MgCl_2$ and 26 μL of DNase I was added and incubated at 4°C for 20 minutes. The lysate was then centrifuged at 50,000Xg for 30 minutes at 4°C. Supernatant was transferred to a new tube and 50 μL of equilibrated HaloLink Resin beads were added and incubated for 4-6 hours at 4°C. After incubation, lysate was centrifuged, and resin was transferred to a new tube. Resin was washed in filtered Wash buffer (50 mM Tris pH 8.0, 1M NaCl, 2 mM $MgCl_2$) four times and eluted in 250 μL of Binding Buffer. For cleavage of AcTEV tag, 3 μL (30 units) of AcTEV protease was added to the eluted beads and incubated on a nutator at 4°C overnight. The next day tube was centrifuged and the supernatant containing purified Pumilio was transferred to new tube and 100% glycerol was added to the eluted protein for a final glycerol concentration of 20%. Protein was aliquoted, and flash frozen in liquid nitrogen and stored at −80°C.

Bruno Protein Purification

Bruno expression plasmid pETM-82 was acquired from EMBL (Chekulaeva et al., 2006). 5 mL of Bruno in pETM-82 in BL21(DE3) was grown overnight at 37°C. This culture was added to 1000 mL of LB-Kanamycin media. Cells were shaken at 220 rpm at 37°C for 2-3 hr or until OD_{600}~0.8. The culture was then cooled down to 25°C.0.5 mM IPTG was added to induce the cells and shaken at 220 rpm at 25°C for 3 hours. The cells were then centrifuged at 4000xg for 20 minutes at 4°C in 50 mL aliquots. The pellet was re-suspended in

3 mL of re-suspension buffer (20 mM Na phosphate, 50 mM NaCl, 20 mM imidazole, 10 ul of 500 mg/ml pH 7.4) and sonicated at 20% intensity for 20 s for 3 times and pulsed for 20 s for 3 times using 1/8 probe, making sure the cell suspension is on ice throughout sonication. The suspension was then centrifuged at 10,000xg for 10 minutes for 4°C. Meanwhile, the His GraviTrap column was equilibrated with 10 mL binding buffer (20 mM Na phosphate, 50 mM NaCl, 20 mM imidazole, 10 ul of 500 mg/ml pH 7.4). The supernatant was added to the column and washed with increments of 1 ml, 4 mL and 5 mL of binding buffer. The protein was then eluted using the following washes; twice with 1 mL of elution buffer (1), twice with 1 mL of elution buffer (2) and three times with 1 mL of elution buffer (3).

Elution Buffer (1): 20 mM $NaPO_4$, 50 mM NaCl, 150 mM imidazole, pH 7.4
Elution Buffer (2): 20 mM $NaPO_4$, 50 mM NaCl, 300 mM imidazole, pH 7.4
Elution Buffer (3): 20 mM $NaPO_4$, 50 mM NaCl, 500 mM imidazole, pH 7.4

The last two fractions contained purified Bruno protein. 100% glycerol was added to the eluted protein for a final glycerol concentration of 20%. The eluted protein sample was de-salted using the PD-10 column. Protein was aliquoted, and flash frozen in liquid nitrogen and stored at −80°C.

Electrophoretic mobility shift assays (EMSA)

RNA oligonucleotides were end-labeled using T4 Kinase with ATP [γ-^{32}P]. Excess ATP was eliminated by using G-25 Sephadix Columns. All RNA-binding reaction was performed in 1X Binding Buffer (50mM Tris pH 7.5, 150mM NaCl, 2mM DTT, 0.1mg/µl BSA, 0.001% Igepal CA-630, 0.5 µL of dIdC and 0.5 µL of yeast t-RNA). RNA and purified protein were incubated for 20 minutes at RT and then ran on a 6% native polyacrylamide TBE gel at 150V for 4 hours at 4°C. The gel was then dried onto Whatmann filter paper and exposed to a phosphor screen overnight. A Typhoon Trio imager was used to image the EMSAs.

Poly(A) tail length (PAT) Assay

500ng of DNase treated RNA was reverse transcribed using Super Script III but instead of using oligo (dT), 5 µL of anchored Oligo (dT) primer was used for each sample (Rangan et al., 2008). 2 µL of cDNA was then amplified using 0.5 µL of gene specific forward primer, 0.5 µL of anchored Oligo(d)T, 0.5 µL of 10 µM dNTP and 0.125 µL Taq Polymerase and 2.5 µL 10XTaq Polymerase Buffer. The thermal cycling conditions for PCR was 95°C for 30 s, 30 cycles of 95°C for 30 s, 2° below T_m of primer for 30 s, 65°C for 1.5 minutes, and 1 cycle of 65°C for 4 minutes. After PCR, 2.8 µL of Orange-G dye was added to each sample and 10 µL of PCR product was ran on a 2.5% agarose gel. The gel was post-stained with ethidium bromide for 20 minutes, and then washed three times with H2O prior to imaging.

RNA-Immuno-precipitation (RIP)-qPCR

Each IP experiment was carried out in 100 pairs of wild-type ovaries. Ovaries were dissected in RNase free 1XPBS. After dissection, PBS was aspirated and 100 µL of RIPA lysis buffer was added to the tissues and homogenized. Another 200 µL of RIPA lysis buffer was added to the lysate and mixed well. The lysate was then centrifuged at 13,000 rpm for 20 minutes at 4°C. 5% of cleared lysate was set aside for Western Blot analysis. 10% of the lysate was set aside and frozen in Trizol as RNA Input for each IP experiment. Remaining lysate was divided equally; one was for IgG control and the other for antibody of interest (AI). 100 µL of Dynabeads Protein A was rinsed 3 times with 400 µL of 1:10 dilution of NP-40 buffer. 25 µL of resuspended beads were added to each AI and IgG containing lysate samples and incubated overnight at 4°C. After incubation, the beads were washed 4 times with 1:10 dilution of NP-40 buffer for 1 minute. An additional two washes for 5 minutes were carried out before re-suspending the beads in 25 µL of NP-40 buffer. 10 µL of beads from each of the samples were used to perform a Western Blot analysis to confirm pull-down. The other 15 µL was used to extract RNA to perform qRT-PCR experiments to show association of RNA with pulled-down protein. Buffers and antibodies used are described below:

RIPA lysis buffer: 10mM Tris-Cl Buffer (pH 8.0), 1mM EDTA, 1% Triton X-100,0.1% Sodium deoxycholate, 0.1% SDS, 140mM NaCl, 1mM PMSF, 1 cOmplete, EDTA-free Protease Inhibitor Cocktail Pill RNase free H_2O.

NP-40 buffer: 50mM Tris-Cl Buffer (pH 8.0), 150mM NaCl, 10% NP-40, 1 cOmplete, EDTA-free Protease Inhibitor Cocktail Pill, RNase free H_2O.

The following antibodies were added to the lysate and incubated at 4°C for 3 hours; 2.5 µL of rabbit anti-GFP, 1.25 µL of Rabbit IgG, 1 µL of rabbit anti-Bru (Dr. Lilly) or 2 µL rabbit anti-Pum (Lehmann lab).

RNA sequencing and sample library preparation

Total RNA was extracted with Trizol, treated with Turbo DNase and poly(A)$^+$ RNA was isolated by double selection with poly-dT beads, using ~6µg total RNA, which is then followed by first- and second-strand synthesis. Sequencing libraries were prepared using NEXTflex Rapid Illumina DNA-Seq Library Prep Kit. 75 base-pair single-end mRNA sequencing was performed an Illumina NextSeq 500 by the Center for Functional Genomics.

Polysome profiling and Polysome-seq

~80 ovaries were dissected in PBS supplemented with cycloheximide and frozen immediately with liquid nitrogen. Tissue was homogenized in 200 μL of cold lysis buffer consisting of 1x Polysome buffer supplemented with 1% Triton-X and 1 protease inhibitor pill per 10 mL of buffer. The lysate was centrifuged at 15,000 x g at 4°C for 10 minutes. 20% of lysate was kept aside for "Input RNA" libraries. 750 μL of cleared lysate was loaded onto 10%–50% sucrose gradients (500 mM KCl; 15 mM Tris-HCl, pH 7.5; 15 mM MgCl2; and 100 μg/ml cycloheximide) in Beckman Coulter 9/16x3.5 PA tubes (Cat. #331372). Gradients were centrifuged at 35,000xg using a SW41 rotor for 3 hours at 4°C. Gradients were fractionated on a Brandel flow cell (Model #621140007) at 0.75 mls/min and 750 μL was collected for each fraction with the sensitivity settings at 0.5 Abs. RNA was extracted from the fractions using standard acid phenol: chloroform extraction. The RNA pellet was washed with 80% ethanol and air-dried. After air-drying the pellet was dissolved in 10 μL of nuclease-free water. Turbo DNase treatment and library preparation was carried out as described above.

QUANTIFICATION AND STATISTICAL ANALYSIS

Western Blot Analysis

To calculate relative change in HA protein expression of the various transgenes reported in this study, first, ImageJ was used to calculate the arbitrary units (A.U) of PgcHA bands and loading control Vasa bands. Then the HA A.U was divided by the Vasa A.U to calculate relative fold change. Wild-type control A.U was subtracted from each ratio to eliminate background. Western blots were repeated three times with independent biological samples.

Quantitative Real Time-PCR (qRT-PCR) analysis

To calculate fold change in GFP mRNA levels to RP49 mRNA levels, first, the Ct values of technical replicates of each trial was averaged. ΔCt was calculated by subtracting RP49 Ct average from the Ct average of GFP. Then the of the 2^-ΔCt was calculated for each trial. To diminish background, 2^-ΔCt valued form wild-type control was subtracted from GFP and RP49 2^-ΔCt values.

To calculate relative protein levels to mRNA levels, the fold protein change was divided by fold RNA change from qRT-PCR experiment for each biological trial. The average, standard deviation and standard error was then calculated for the three trials.

RNA-Immuno-precipitation (RIP) qPCR analysis

The following calculation was adapted from the Sigma-aldrich Imprint RIP Kit protocol.

1. Each RIP RNA fractions' Ct value was normalized to each of the Input RNA fraction Ct value for the same qPCR Assay (ΔCt) to account for RNA sample preparation differences.

ΔCt [normalized RIP] = Ct [RIP] – (Ct [Input] – Log2 (Input Dilution Factor)), where, Input Dilution Factor = (fraction of the input RNA saved).

2. The % Input for each RIP fraction (linear conversion of the normalized RIP ΔCt) was calculated.

$$\%\text{Input} = 2^{(-\Delta \text{Ct}[\text{normalized RIP}])}$$

3. The normalized RIP fraction Ct value for the normalized background [IgG Ab] fraction Ct value (first ΔΔCt) was adjusted.

$$\Delta\Delta \text{Ct}[\text{RIP}/\text{IgG Ab}] = \Delta \text{Ct}^{[\text{normalized RIP}]} - \Delta \text{Ct}^{[\text{normalized IgG RIP}]}$$

4. IP Fold Enrichment above the sample specific background (linear conversion of the first ΔΔCt) was calculated.

$$\text{Fold Enrichment} = 2^{(-\Delta\Delta \text{Ct}[\text{RIP}/\text{IgG Ab}])}$$

Statistical Analysis

A student's two-tailed t test or population proportion z-test were carried out to calculate significance of results. Standard error was calculated from three independent biological samples for each experiment and is represented by the error bar. *, ** and *** denotes p values less than 0.05, 0.005 and 0.005 respectively. All analysis was carried out using Microsoft Excel.

RNA-seq data analysis

After quality of reads was assessed the RNA-seq reads were aligned via HISAT2 (version 2.1.0) (Kim et al., 2015a) set to be splice aware to UCSC dm6 release 6.01. Count tables were generated using featureCounts (version 3.16.5) (Liao et al., 2014).

Translation Efficiency (TE) Analysis

To determine translation efficiencies (TE), CPMs (counts per million) values were calculated for all polysome-seq libraries. Any transcript having zero reads in any library was discarded from analysis. The $\log_2$ ratio of CPMs between the polysome fraction and total mRNA was calculated and averaged between replicates. This ratio represents TE. After TE of each sample was

calculated and replicates were averaged, TE of *pum*RNAi and *bru*RNAi were compared to that of Control. This ratio represents ΔTE. Targets were defined as transcripts falling greater or less than one standard deviation from the median of ΔTE (Kronja et al., 2014). To discover sequences similar to the *pgc* BRE in the 3′UTR of targets, all annotated 3′UTRs were downloaded from Flybase for all analyzed targets. A list of BREs and PREs that contain the core sequence UGUA was compiled manually through a literature search. Using the R package Biostrings this list was used to generate and apply a position weight matrix (pwm). This pwm was used to score all 10-mers in all of the previously mentioned 3′UTRs. A minimum score of 90% was set as cutoff. Additionally, we manually ensured that the core sequence UGUA was present in all targets above the cutoff. Targets identified from polysome-seq were subsetted from the list of RNAs containing a *pgc*-like BRE in their 3′UTR using a custom R script.

Gene Ontology (GO) Enrichment Analysis

Significant over-represented functional categories of the 212 PRE/BRE containing shared targets of Pum and Bru was carried out using the PANTHER Gene List Analysis tool. Selected GO terms with p value < 0.05 have been shown in Figure 7D.

DATA AND SOFTWARE AVAILABILITY

The accession number for the data reported in this paper is GEO: GSE119458.

Cell Reports, Volume *25*

Supplemental Information

Sequential Regulation of Maternal mRNAs through a Conserved *cis*-Acting Element in Their 3′ UTRs

Pooja Flora, Siu Wah Wong-Deyrup, Elliot Todd Martin, Ryan J. Palumbo, Mohamad Nasrallah, Andrew Oligney, Patrick Blatt, Dhruv Patel, Gabriele Fuchs, and Prashanth Rangan

Flora_Figure S1

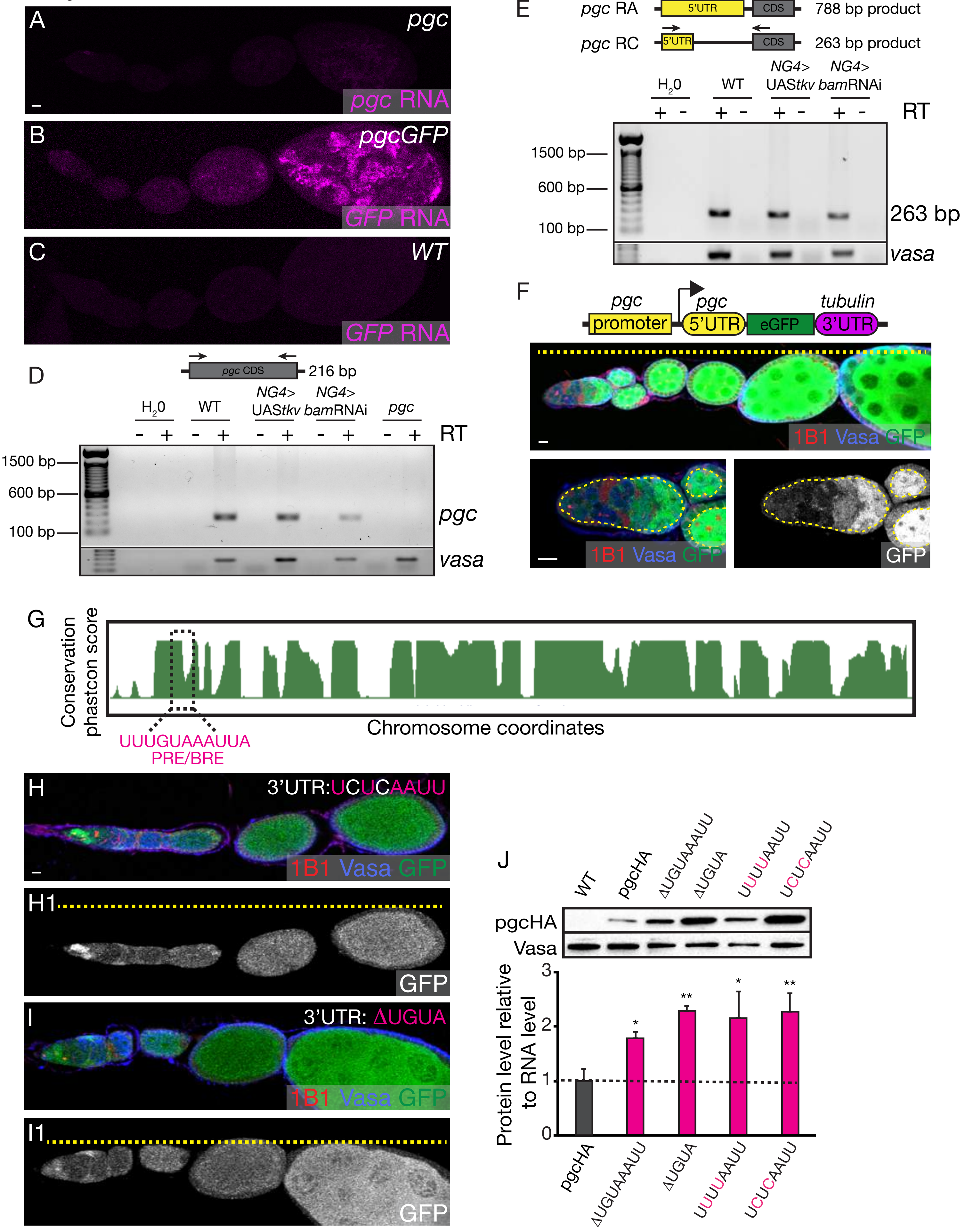

Supplemental Figure 1. Pgc is translationally regulated via its UTRs (Related to Figures 1 and 2) (A) The ovariole of a *pgc* mutant fly probed for *pgc* RNA (magenta) using FISH, show no signal for *pgc* RNA. (B) The ovariole of a *pgcGFP* transgenic fly probed for *GFP* RNA (magenta) using FISH, show similar expression pattern when compared to endogenous *pgc* RNA. (C) The ovariole of a wild-type fly probed for *GFP* RNA (magenta) using FISH, show no signal for *GFP* RNA. (D) RT-PCR of *pgc* CDS was carried out on RNA samples extracted from wild-type, *nosGAL4*>UAS*tkv* and *nosGAL4*>*bam*RNAi show *pgc RNA* is not only present in whole adult ovaries, but also transcribed in GSC and CB enriched tumors. RNA null *pgc* mutant was used as a negative control. RT-PCR of Vasa was carried out as a positive control. (E) RT-PCR of *pgc* 5'UTR was carried out on RNA samples extracted from wild-type, *nosGAL4*>UAS*tkv* and *nosGAL4*>*bam*RNAi. Primers were designed as to show either a 788bp or a 263bp product to confirm what 5'UTR length of *pgc RNA* was being expressed during oogenesis. Results showed presence of short version of *pgc 5'* UTR in whole adult ovaries, GSC and CB enriched tumors. RNA null *pgc* mutant was used as a negative control. RT-PCR of Vasa was carried out as a positive control. (F) The ovariole of a transgenic fly created by fusing GFP to the *pgc* 5' and *tub* 3'UTR and under the control of the *pgc* promoter was stained with 1B1 (red) which marks the spectrosomes and fusomes, Vasa (blue) which marks the germline and GFP (green) which marks Pgc expressing cells. There is a loss of GFP regulation throughout oogenesis, including at the earliest stages (yellow dashed line). (G) A phylogenetic analysis of *pgc* 3'UTR of all Drosophilids identified a conserved sequence that can potentially bind both RBPs, Pum and Bru. (H) The ovariole of a transgenic fly created by fusing GFP to *pgc* 5' and *pgc* 3'UTR where the UGUA sequence was mutated to UCUC (3'UTR: UCUCAAUU) and driven under *pgc* promoter stained with 1B1 (red), Vasa (blue) and GFP (green) shows loss of GFP regulation throughout oogenesis. GFP channel shown in H1. (I) Ovariole of a transgenic fly created by fusing GFP to *pgc* 5' and *pgc* 3'UTR where the UGUA sequence was deleted (3'UTR: ΔUGUA) and driven under *pgc* promoter stained with 1B1 (red), Vasa (blue) and GFP (green) shows loss of GFP regulation throughout oogenesis. GFP channel shown in I1. (J) Normalized protein expression to RNA levels shows that either deletions or mutations in the PRE/BRE sequence of the 3'UTR of *pgc* results in a significant upregulation of Pgc reporter protein when compared to FL 3'UTR. The graph represents an average generated from three independent biological replicates. The error bars are the standard error calculated from these replicates. A student t-test statistical analysis was performed. * indicates p-value <0.05 and ** indicates p-value <0.005. Scale bars: 10μm.

Flora_Figure S2

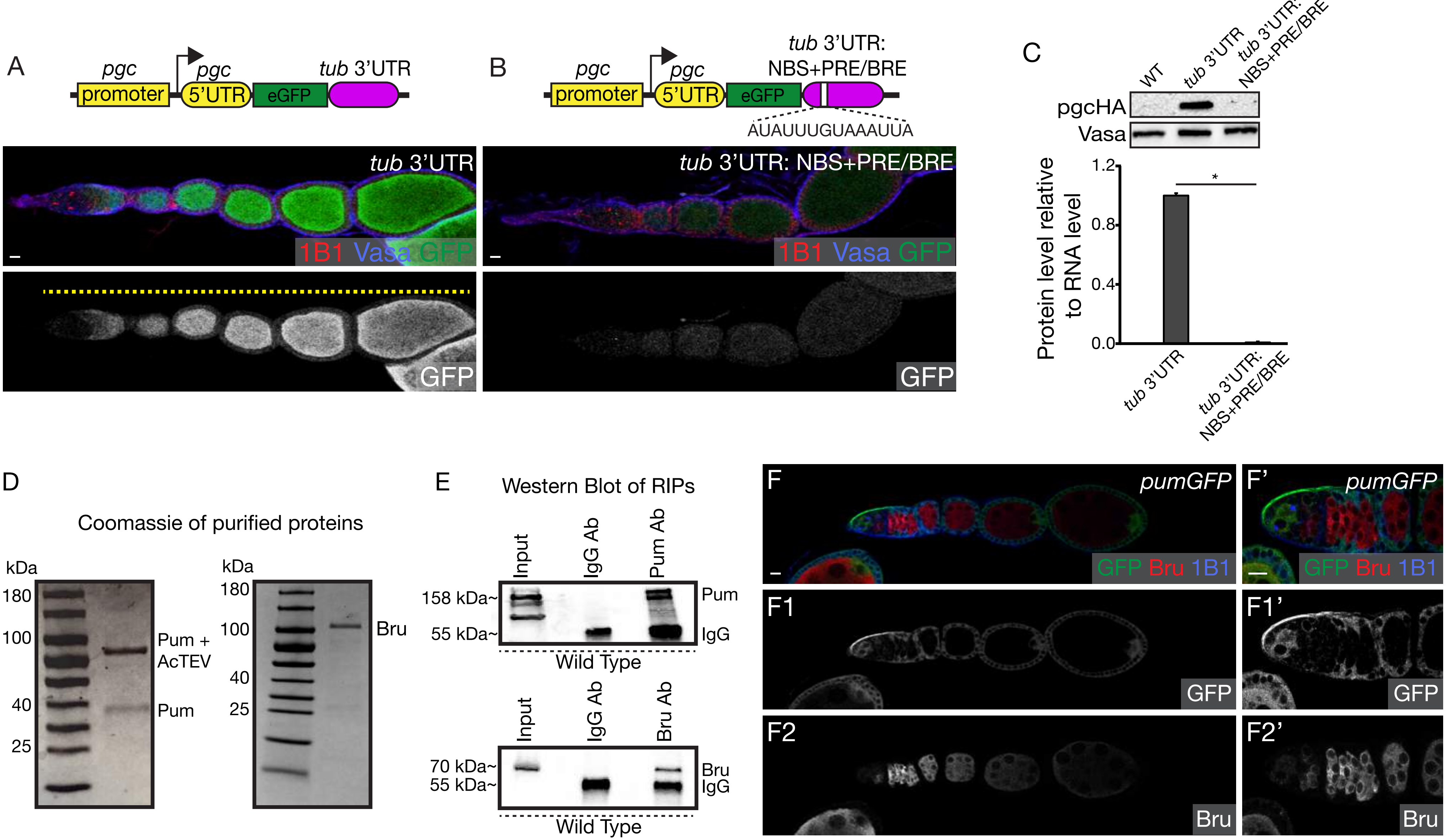

Supplemental Figure 2. A cis-element in the *pgc* 3'UTR that binds both Pum and Bru is required for translational control throughout oogenesis (Related to Figure 2) (A) The ovariole of a transgenic fly created by fusing GFP to the *pgc* 5' and *tub* 3'UTR and under the control of the *pgc* promoter stained with 1B1 (red) which marks the spectrosomes and fusomes, Vasa (blue) which marks the germline and GFP (green) shows a loss of GFP regulation throughout oogenesis (yellow dashed line). GFP channel is shown in A1. (B) The ovariole of a transgenic fly created by fusing GFP to the *pgc* 5' and *tub* 3'UTR that contains the NBS and PRE sequences and under the control of the *pgc* promoter stained with 1B1 (red), Vasa (blue) and GFP (green). Insertion of the sequence is sufficient translation repression. (C) Normalized protein expression to RNA levels shows that insertion of the NBS+PRE/BRE sequence in the *tub* 3'UTR results in significant repression of reporter protein when compared to control. The graph represents an average generated from three independent biological replicates. The error bars are the standard error calculated from these replicates. A student t-test statistical analysis was performed. * indicates p-value <0.05. (D) Commasie stained SDS-PAGE gels shows successful purification of recombinant Pum (left) and recombinant Bru protein (right). (E) Western Blot shows successful pull-down of Pum (top) and Bru (bottom) from wild-type ovary lysates using anti-Pum and anti-Bru antibody, respectively. (F-F2') *pumGFP* transgene fly stained with Bru (red), 1B1 (blue) and GFP (green) which marks Pum expressing cells shows that Pum protein is expressed in high levels in the earliest stages of oogenesis and lowers in later differentiating stages while Bru protein levels are low in early stages and increases from the 8-cell cyst stages and onwards. F1-F1' and F2-F2' shows GFP and Bru channels in gray. Scale bars: 10µm.

Flora_Figure S3

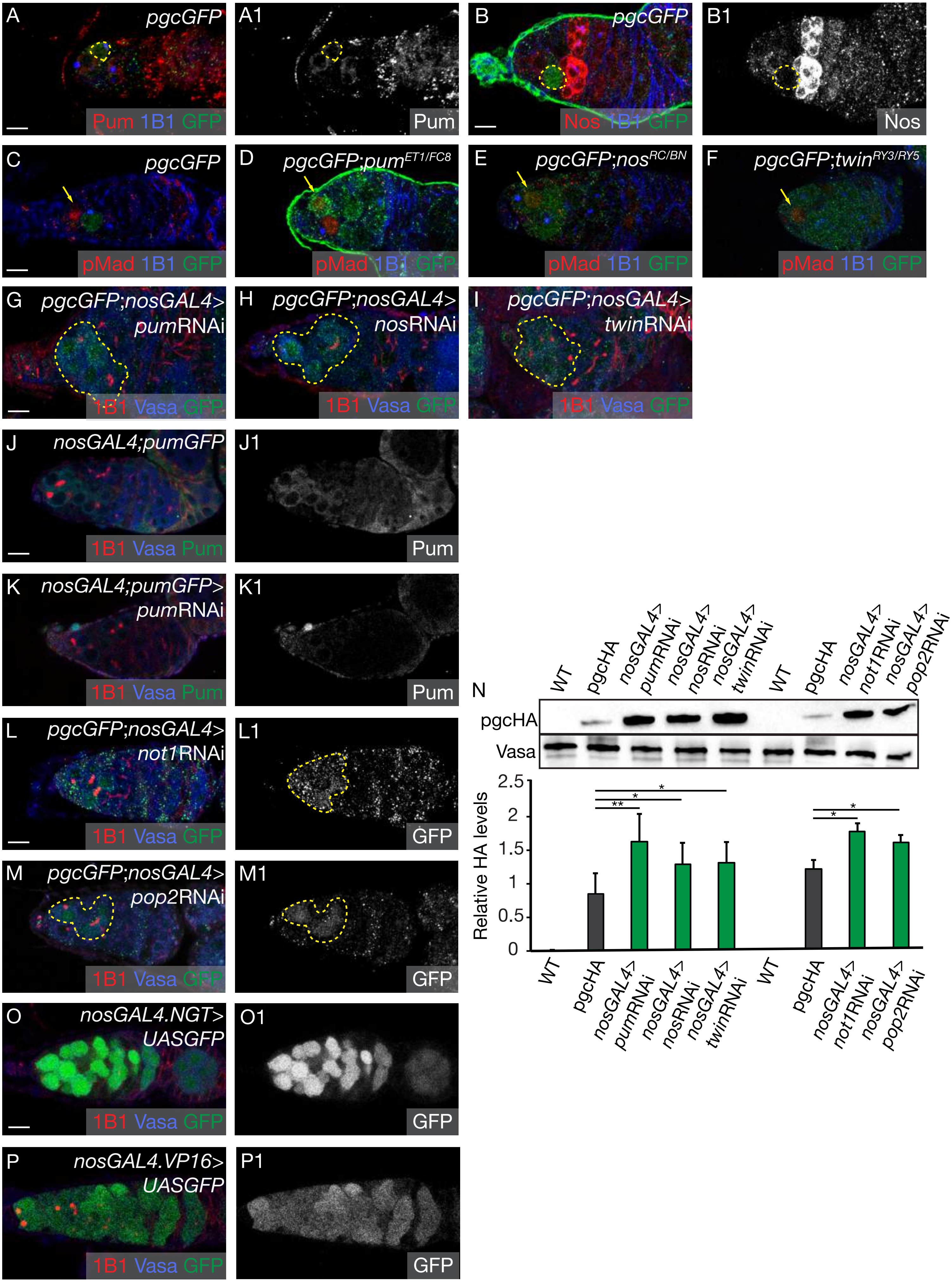

Supplemental Figure 3. Pum and its co-factor Nos regulate Pgc translation in the GSCs (Related to Figure 3) (A, A1) The germarium of *pgcGFP* fly stained with Pum (red), 1B1 (blue) which marks fusomes and spectrsomes and GFP (green) which marks Pgc expressing cells shows high levels of Pum protein in the GSC, pre-CB (yellow dashed circle) 2- to 4-cell cysts. Pum staining is shown in gray in A1. (B, B1) The germarium of *pgcGFP* fly stained with Nos (red), 1B1 (blue) and GFP (green) which marks Pgc expressing feels shows Nos protein is present throughout the germarium except for the GFP expressing pre-CB cell (yellow dashed circle). Nos staining is shown in gray in B1. (C) The germarium of *pgcGFP* fly stained with pMad (red) which marks GSCs, 1B1 (blue) and GFP (green) shows GSCs do not express GFP (yellow arrow). (D-F) The germaria of *pgcGFP, pgcGFP; pum, pgcGFP; nos* and *pgcGFP; twin* stained with pMad (red), 1B1 (blue) and GFP (green) show that in absence of Pum and its co-factors, there is a loss of GFP regulation in the GSCs (yellow arrow). (G-I) The germaria of *pgcGFP; nosGAL4>pum*RNAi, *pgcGFP; nosGAL4>nos*RNAi and *pgcGFP; nosGAL4>twin*RNAi flies stained with 1B1 (red), Vasa (blue) and GFP (green) show aberrant expression of GFP in the earliest stages of oogenesis, including the GSCs (outlines in yellow dashed line). (J, J1) The germarium of *nosGAL4* flies stained with 1B1 (red), Vasa (blue) and Pum (green) shows Pum being expressed in high levels in somatic cells and in the earliest stages of oogenesis. Pum channel shown in J1. (K, K1) The germarium of *nosGAL4; pum*RNAi flies stained with 1B1 (red), Vasa (blue) and Pum (green) shows Pum is significantly downregulated in the ovaries that contain germline. Pum channel shown in K1 (L, L1) The germarium of germline depleted *not1* ovary stained with 1B1 (red), Vasa (blue) and GFP (green) shows aberrant expression of GFP in the GSCs and 4-cell cysts (100%, n= 25 germaria) (outlined in yellow dashed line). GFP channel showed in gray scale in A1. (M, M1) The germarium of germline depleted *pop2* ovary stained with 1B1 (red), Vasa (blue) and GFP (green) shows aberrant expression of GFP in the GSCs to the 4-cell cyst stages (100%, n= 25 germaria) (outlined in yellow dashed line). GFP channel showed in gray scale in B1. (N) A western blot analysis shows a significant upregulation of Pgc reporter protein in the germline depletion of *pum*, *nos*, *twin*, *not1,* and *pop2* ovaries when compared to *pgcGFP.* The graph represents an average generated from three independent biological replicates. The error bars are the standard error calculated from these replicates. A student t-test statistical analysis was performed. * indicates p-value <0.05 and ** indicates p-value <0.005. (O, O1) The germarium of *nosGAL4.NGT>UAS-GFP* stained with 1B1 (red), Vasa (blue) and GFP (green) shows no difference in GFP expression levels in the germarium. GFP channels shown in O1. (P, P1) The ovariole of *nosGAL4.VP16>UAS-GFP* stained with 1B1 (red), Vasa (blue) and GFP (green) shows no difference in GFP expression levels in the germarium. GFP channels shown in P1. Scale bars: 10µm.

Flora_Figure S4

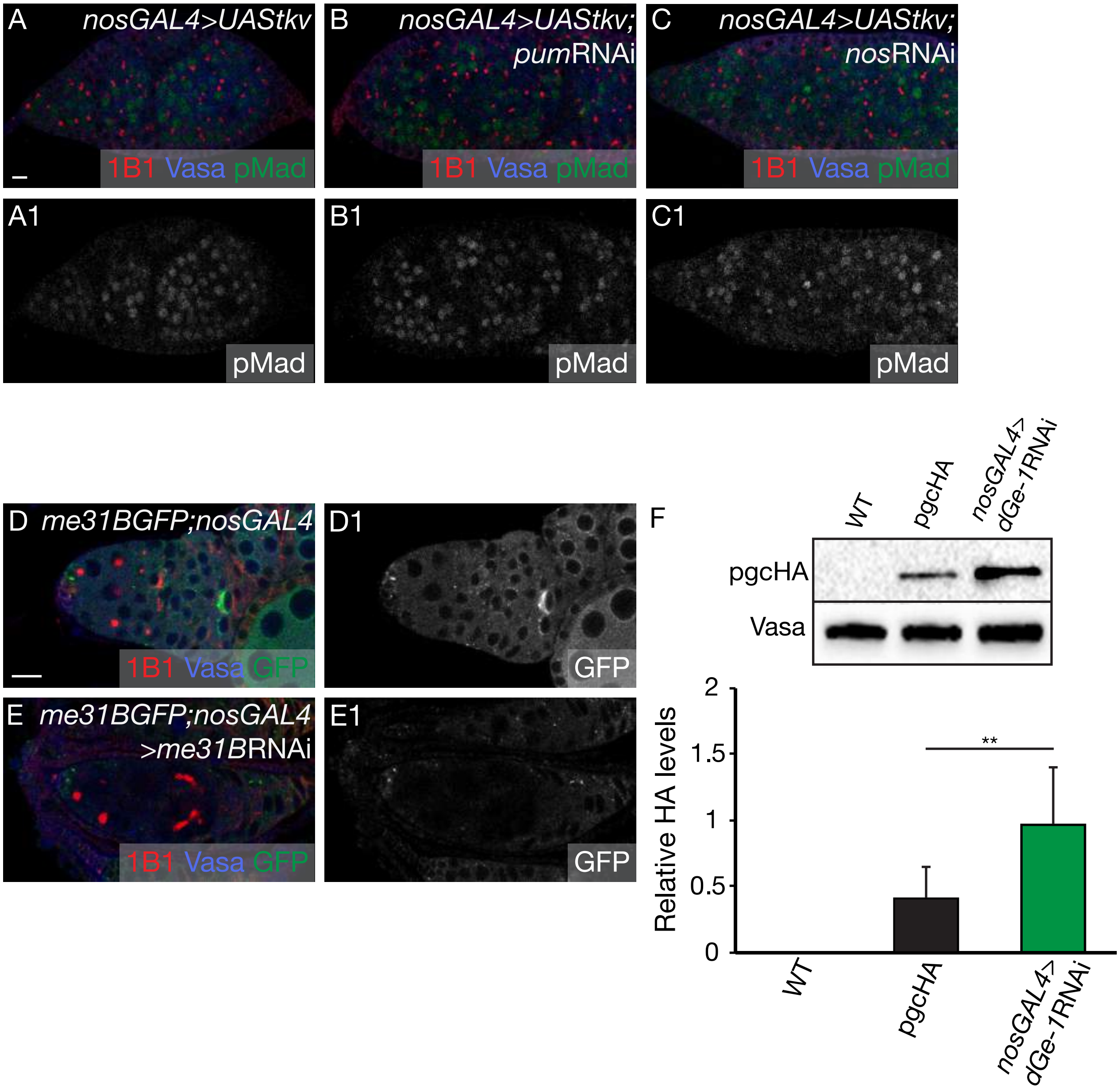

Supplemental Figure 4. Me31B cooperates with the decapping protein dGe-1 and *pgc* 5'UTR to mediate repression in the GSCs and early differentiating cysts (Related to Figures 3 and 4) (A, A1) The germarium of *nosGAL4*>UAS-*tkv* ovary stained with 1B1 (red) which marks the spectrosomes and fusomes, Vasa (blue) which marks the germline and pMad (green) which marks GSCs shows a tumor enriched with GSCs. pMad channel shown in A1. (B-C1) The germaria of *nosGAL4*>UAS-*tkv; pum*RNAi, *nosGAL4*>UAS-*tkv; nos*RNAi ovary stained with 1B1 (red), Vasa (blue) and pMad (green) shows a tumor of enriched with GSCs. pMad channel shown in B1 and C1. (D, D1) The germarium of *me31BGFP*-trap; *nosGAL4* ovary stained with 1B1 (red), Vasa (blue) and GFP (green) which marks Me31B expressing cells shows Me31B being expressed in both the germline and somatic cells of the germarium. GFP channel shown in D1. (E, E1) The germarium of *me31BGFP*-trap; *nosGAL4* depleted of *me31B* via RNAi stained with 1B1 (red), Vasa (blue GFP (green) Me31B being expressed only in the somatic cells of the germarium confirming germline knockdown of Me31B via RNAi. GFP channel shown in E1. (F) A western blot analysis shows a significant upregulation of Pgc reporter protein in the germline depletion of *dGe-1* ovaries when compared to *pgcGFP.* The graph represents an average generated from three independent biological replicates. The error bars are the standard error calculated from these replicates. A student t-test statistical analysis was performed. * indicates p-value <0.05 and ** indicates p-value <0.005. We were unsuccessful in isolating stable lysates from Me31B depleted ovaries to carry out a WB analysis. Scale bars: 10μm.

Flora_Figure S5

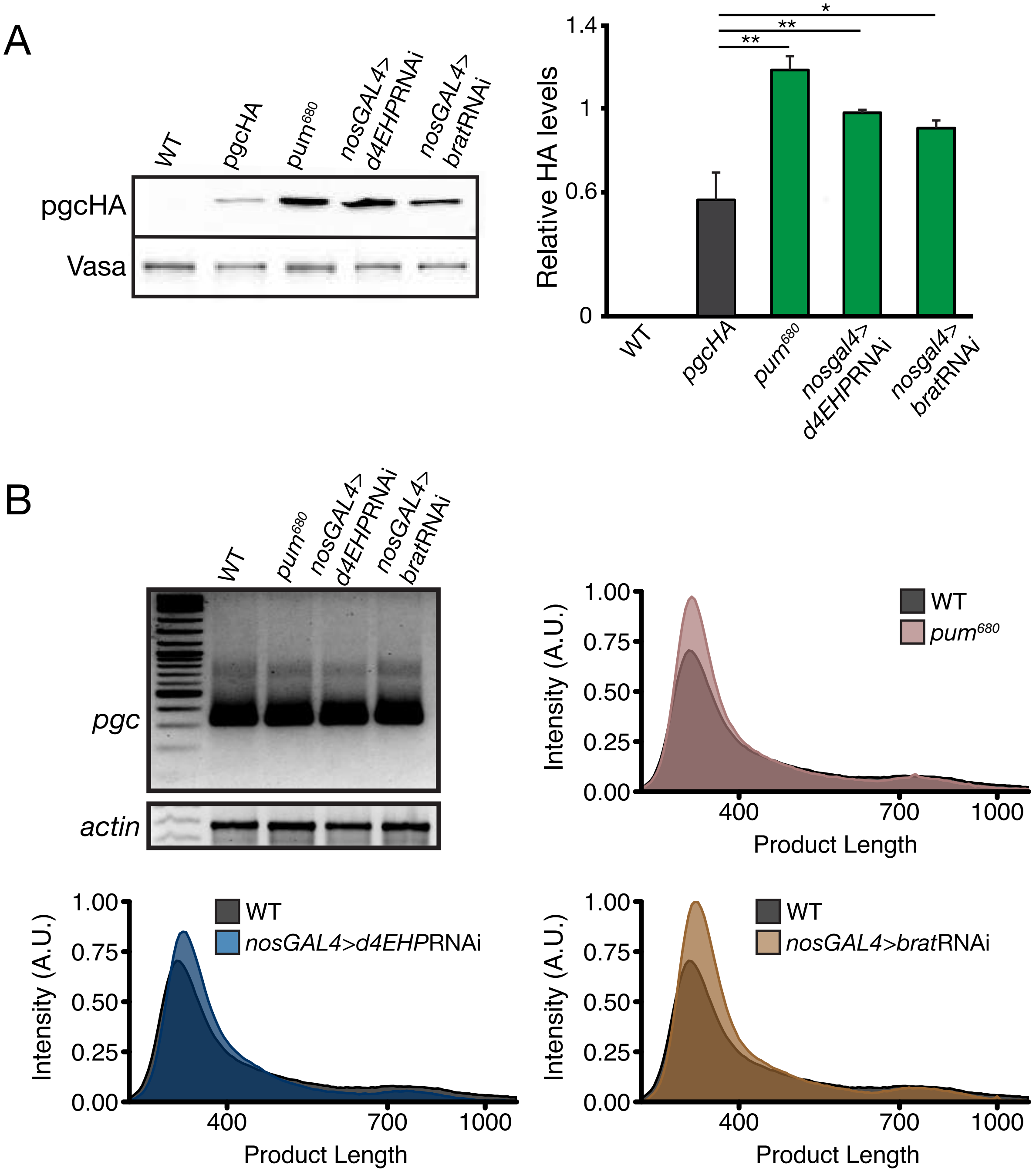

Supplemental Figure 5. Pum and its co-factor Brat regulate Pgc translation in the 4- to 16-cell cysts (Related to Figure 5) (A) A western blot analysis shows a significant upregulation of Pgc reporter protein in pum^{680} and the germline depletion of *brat* and *d4EHP* ovaries when compared to *pgcGFP.* The graph represents an average generated from three independent biological replicates. The error bars are the standard error calculated from these replicates. A student t-test statistical analysis was performed. ** indicates p-value <0.005. (B) PAT assay analysis of *pgc* poly(A)-tail length in wild-type, pum^{680} and germline depletions of d4EHP and Brat show that loss of these factors do not result in any change of poly(A)-tail length of *pgc*.

Flora_Figure S6

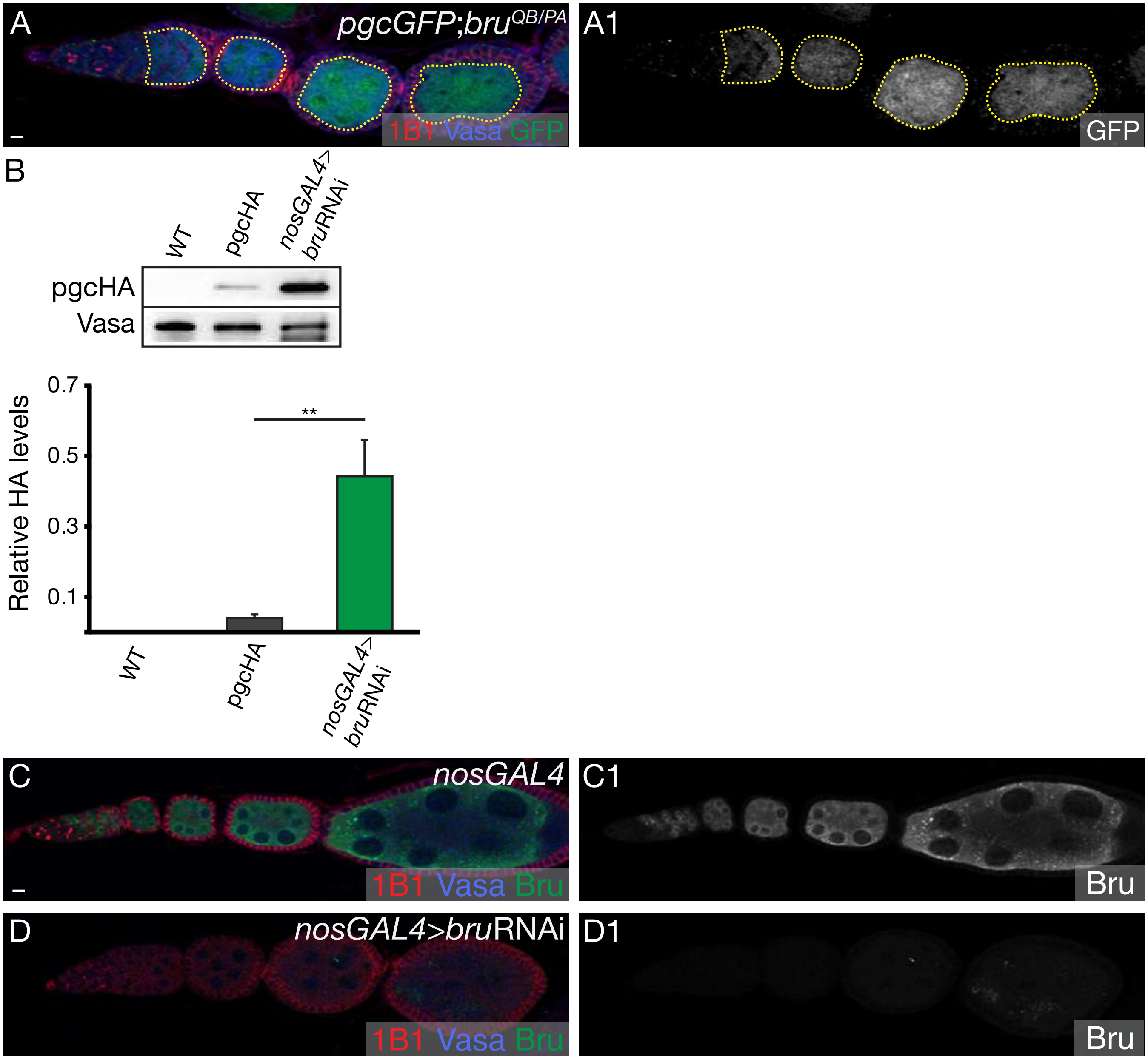

Supplemental Figure 6. Bru and Cup regulate Pgc translation in the later stages of oogenesis (Related to Figure 6) (A, A1) The ovariole of *pgcGFP; bru*RNAi$^{QB/PA}$ stained with 1B1 (red) which marks the spectrosomes and fusomes, Vasa (blue) which marks the germline and GFP (green) which marks Pgc expressing cells shows upregulation of reporter expression from 16-cell cyst onwards (outlined in yellow dashed line). GFP channel shown in A1. (B) A western blot analysis shows a significant upregulation of Pgc reporter protein in the germline depletion of Bru ovaries when compared to *pgcGFP.* We were unsuccessful in isolating stable lysates from Cup depleted ovaries to carry out a WB analysis. The graph represents an average generated from three independent biological replicates. The error bars are the standard error calculated from these replicates. A student t-test statistical analysis was performed. ** indicates p-value <0.005. (C, C1) The ovariole of control *nosGAL4* ovary stained with 1B1 (red), Vasa (blue) and Bru (green) shows Bru being expressed from 16-cell cyst and onwards. Bru channel shown in C1. (D, D1) The ovariole of *nosGAL4>bru*RNAi stained with 1B1 (red), Vasa (blue) and Bru (green) shows little or no Bru expression in the ovariole. GFP channel shown in D1. Scale bars: 10μm.

Flora_Figure S7

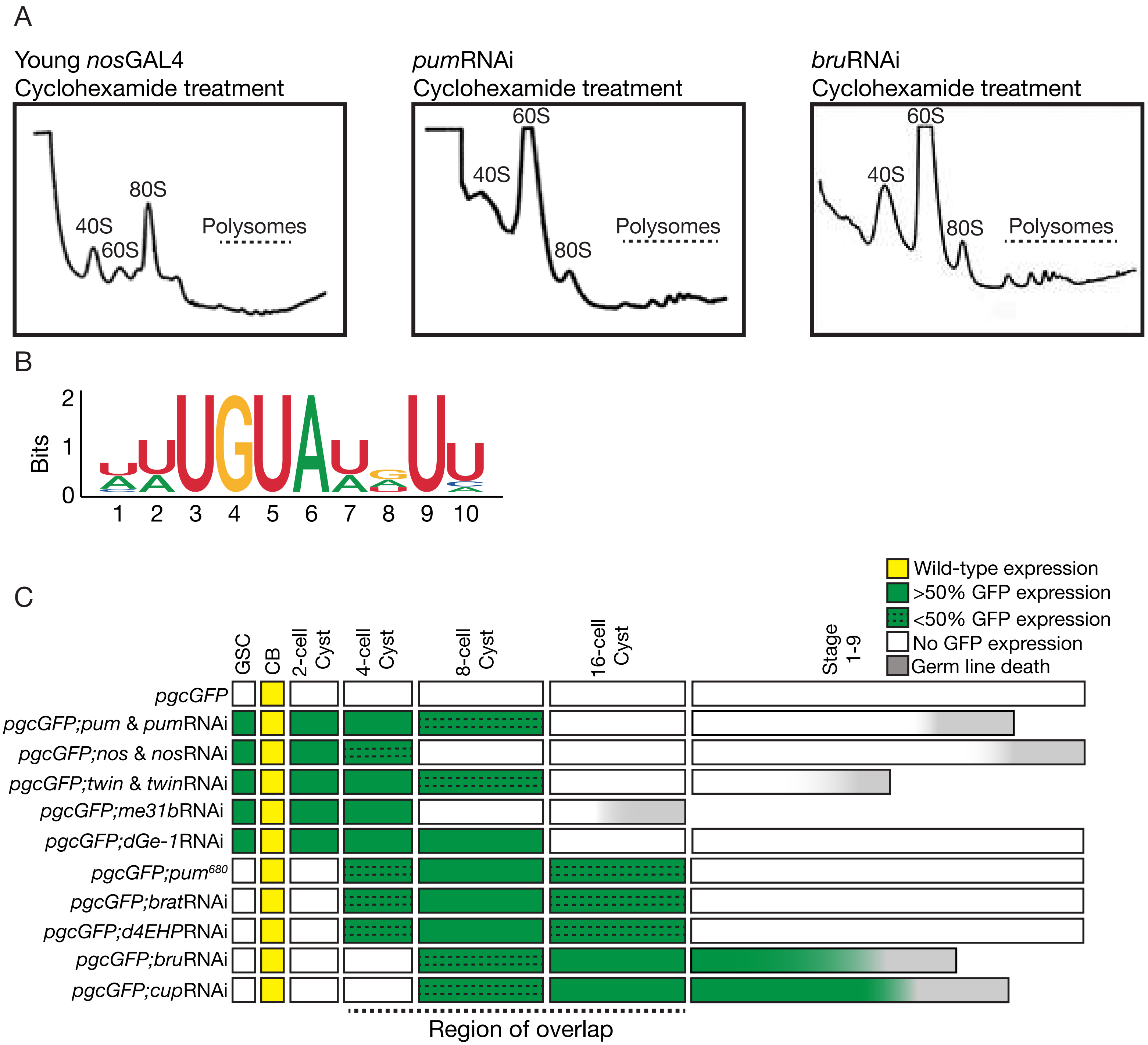

Supplemental Figure 7. A class of germline RNAs are similarly regulated by both Pum and Bru (Related to Figure 7) (A) Polysome profile traces of young wild-type, *pgcGFP; nosGAL4>pum*RNAi, and *pgcGFP; nosGAL4>bru*RNAi ovaries treated with cyclohexamide. (B) The logo of the sequences used to identify shared targets of Pum and Bru mediated regulation that contain a sequence similar to the PRE/BRE sequence identified in the *pgc* 3'UTR. (C) A developmental profile of GFP expression in *pgcGFP, pgcGFP;* $pum^{ET1/FC8}$ and germline knockdown of *pum, pgcGFP;* $nos^{RC/BN}$ and germline knockdown of *nos* and *pgcGFP;* $twin^{ry3/ry5}$ and germline knockdown of *twin, pgcGFP; me31B*RNAi, *pgcGFP; dGe-1*RNAi, *pgcGFP;* pum^{680}, *pgcGFP; nosGAL4>brat*RNAi, *pgcGFP; nosGAL4>d4EHP*RNAi, *pgcGFP; nosGAL4>bru*RNAi, and *pgcGFP; nosGAL4>cup*RNAi ovarioles show temporal and sequential loss of GFP regulation in different stages of oogenesis where these trans-acting factors mediate *pgc* regulation.
